# Supplementary material for: Rational design of a sensitivity-enhanced tracer for discovering efficient APC–Asef inhibitors
Source: Nat Commun. 2022 Aug 24;13:4961. doi: 10.1038/s41467-022-32612-6 (PMC9402538; doi:10.1038/s41467-022-32612-6)
Supplement: Supplementary file 1 — Supplementary information [file 41467_2022_32612_MOESM1_ESM.pdf]

Supplementary Information

Rational Design of a Sensitivity-Enhanced Tracer for Discovering Efficient APC–Asef Inhibitors

J. Zhang et al.

Table of Contents

Supplementary Methods 3

1. Peptides Synthesis and Purification. 3
2. Molecular modeling and molecular dynamics 17
3. Fluorescence Polarization Competition Assays of p53-MDM2 17

Supplementary Fig. 1. Interaction analysis of tracer1, tracer 4 and tracer 5 complexed with APC 18
Supplementary Fig. 2. Binding curves for tracers to APC (303-739) determined by FP assay 18
Supplementary Fig. 3. Electron density maps of tracer 7 and MAI-516 19
Supplementary Fig. 4. Binding modes of MAI-400 complexed with APC. 19
Supplementary Fig. 5. Original plot of FP against concentration for Fig. 3b from three individual experiments. 20
Supplementary Fig. 6. Binding modes of MAI-516 complexed with APC 20
Supplementary Fig. 7. Overlay cocrystal structure of MAI-516 complexed with APC and MAI-400 complexed with APC (PDB code 5Z8H) 21
Supplementary Fig. 8. Representative isothermal titration calorimetry (ITC) data 22
Supplementary Fig. 9. Effect of MAI-516 and MAIT-516 on APC-Asef interaction 23
Supplementary Fig. 10. MAI-516 dose not inhibit the migration of SW480 cells 24
Supplementary Fig. 11. Effect of MAIT-516 on cell migration of normal intestinal epithelial cell HIEC-6 evaluated by wound healing and transwell migration assay 25
Supplementary Fig. 12. Effect of MAIT-516 on cell migration of colorectal cancer cells expressed full-length APC evaluated by wound healing and transwell migration assay 26
Supplementary Fig. 13. Effect of MAIT-516 on cell migration of colorectal cancer cells expressed truncated APC evaluated by wound healing and transwell migration assay 27
Supplementary Fig. 14. Effect of MAIT-516 on transwell migration of SW480 stable cells expressing FLAG-tagged APC 1-1338 and W593A and N641 mutants 28
Supplementary Fig. 15. MAIT-516 does not affect the degradation, localization, nuclear translocation and transcriptional activity of β-catenin in SW480 cells 29
Supplementary Fig. 16. MAIT-516 does not affect cell viability 30
Supplementary Fig. 17. Effect of MAIT-516 on apoptosis and cell differentiation 31
Supplementary Fig. 18. The binding curves of five tracers to GST-MDM2 3-150. 32
Supplementary Fig. 19. The significant conformation of PS-P53-9I to MDM2 by molecular dynamics simulations 33
Supplementary Fig. 20. Tracer PS-P53-9I is more sensitive in differentiating peptides P53 and PS5 in FP assay 34
Supplementary Table 1. The binding affinity and dynamic range for tracer 8–14 35
Supplementary Table 2. Thermodynamic data for the binding of tracer 1 and tracer 7 to APC. 35
Supplementary Table 3. Data collection and refinement statistics for co-crystals of APC–tracer 7 and APC–MAI-516. 36
Supplementary Table 4. The IC_{50} values of peptides with different tracers in FP assay. 37
Supplementary Table 5. The structure, HPLC, and MS data of compounds MAI-451–MAI-473. 38
Supplementary Table 6. The structure and binding affinity for tracers of MDM2. 42
Supplementary Table 7. The IC_{50} values of peptides to MDM2 with different tracers in FP assay. 43
Supplementary Table 8. Sequence of real-time PCR primers used in the study. 43

Supplementary References 44

Supplementary Methods

1. Peptides Synthesis and Purification.

Reagents and solvents used for synthesis were obtained from commercial suppliers and used without further purification. Proton NMRs were obtained using 600 MHz instruments. Carbon NMRs were obtained using a Bruker 151 MHz NMR spectrometer. Chemical shifts are reported in ppm, and coupling constants are reported in Hz. Mass spectrometry was performed on high-resolution electron impact (EI) or electrospray ionization (ESI) mass spectrometry instruments available at the site of synthesis.

General procedure A for the synthesis of peptide compounds. The desired peptides (MAI-501–MAI-522, p53) were conducted in the solid phase using Fmoc-Amide AM Resin (0.35 mmol/g, 572mg) as the solid support. The resin was first swelled in DMF (5 mL) for 5 min, then treated with 20% piperidine in DMF (5 mL) for 15 min. The procedure was repeated one time to completely remove Fmoc protecting group. The beads were washed with DCM (5 mL×3) and DMF (5 mL×3). By coupling of the 0.4 mmol N-Fluorenylmethyloxycarbonyl (Fmoc) protected amino acids using 0.6 mmol O-Benzotriazole-N,N,N',N'-tetramethyl-uronium-hexafluorophosphate (HBTU) or 0.6 mmol Benzotriazol-1-yl-oxytripyrrolidinophosphonium hexaflu (PyBOP) as the coupling reagent and 0.8 mmol N,N-Diisopropylethylamine (DIPEA) as the base. Fmoc-protecting groups were removed using 20% piperidine in dimethylformamide (DMF) after each coupling step. After the peptides were assembled, the N-terminal amines were acylated with benzoyl chloride in DIPEA. The peptides were then cleaved from the resin and deprotected using a mixture of trifluoroacetic acid (TFA), water, and triisopropylsilane (TIS) (95:2.5:2.5, v/v/v) for 2.5−3 h. The crude peptides were purified by reversed-phase high-performance liquid chromatography (RP-HPLC) and lyophilized to give a white powder. Purified peptides were analyzed by analytical RP-HPLC, and the integrity of the peptides was checked by negative ion electrospray ionization mass spectrometry (ESI-MS). All peptides were purified to ≥98% purity using RP-HPLC and were verified using H-NMR, C-NMR, ESI-MS and HRMS. The use of HBTU poses a skin irritation risk. The use of DIPEA and TFA pose a risk of inhalation toxicity.

General procedure B for the synthesis of tracer compounds. The general synthesis of tracer 1-14, PS-P53-7G, PS-P53-8H, PS-P53-9I, PS-P53-10J, PS-P53-11K were conducted in the solid phase using Rink Amide-MBHA resin (0.646 mmol/g, 100mg) as the solid support. The resin was first swelled in DMF (5 mL) for 5 min, then treated with 20% piperidine in DMF (5 mL) for 15 min. The procedure was repeated one time to completely remove Fmoc protecting group. The beads were washed with DCM (5 mL×3) and DMF (5 mL×3). The Fmoc protected regular amino acid (0.13 mmol), DIC (0.26 mmol) and HOBt (0.26 mmol) were dissolved in 2 mL DMF for 5 min and the solution was added to the resin. The reaction vessel was shaken for 3 hours until the reaction was complete. The reaction cycles were repeated to assemble the desired peptides. After installation of the last Fmoc protected amino acid, the Fmoc group was removed, and FITC (0.13 mmol) in 5 mL DMF and DIPEA (0.39 mmol.) was added to the resin to react overnight. After washing with DMF (5 mL×3) and DCM (5 mL×3), the resin was cleaved using TFA/DCM/TIS (6 mL, 50:48:2, v/v/v) for 3.5 h. The solution was collected, and the resin was washed with DCM (5 mL×2). The solution was combined and evaporated under air flow, and the crude product was subsequently analyzed (1 mL/min flow rate) and purified (16 mL/min flow rate) by Water HPLC system equipped with both analytic and preparative modules. The gradient eluting method was as follows: 5% of solvent B (0.1% TFA in acetonitrile) in A (0.1% TFA in water) was increased to 100% over 50 min was performed. The desired peptides were collected and lyophilized on a Labconco lyophilizer, with the purity determined to be >95% by analytical HPLC. The use of DIC poses acute inhalation toxicity.

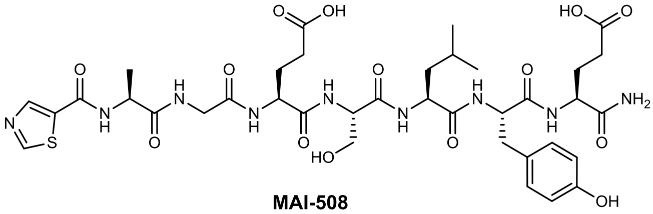

MAI-508

(4S,7S,10S,13S,16S)-4-carbamoyl-7-(4-hydroxybenzyl)-13-(hydroxymethyl)-10-isobutyl-6,9,12,15-tetraoxo-16-(2-((S)-2-(thiaz-ole-5-carboxamido)propanamido)acetamido)-5,8,11,14-tetraazanonadecanedioic acid (MAI-508). General procedure A. White powder, 26.5 mg, 15.1% yield. ^{1}H NMR (600 MHz, DMSO-d_6) δ ppm 9.21 (s, 1H), 9.18 (s, 1H), 8.89 (d, J = 6.9 Hz, 1H), 8.32 (t, J = 5.8 Hz, 1H), 8.06 (d, J = 7.6 Hz, 1H), 8.00 (d, J = 7.4 Hz, 1H), 7.88 (d, J = 7.9 Hz, 1H), 7.83 (d, J = 7.9 H

z, 1H), 7.80 (d, *J* = 8.0 Hz, 1H), 7.08 (d, *J* = 5.9 Hz, 2H), 7.00 (d, *J* = 8.5 Hz, 2H), 6.63 (d, *J* = 8.5 Hz, 2H), 5.13 (t, *J* = 5.5 Hz, 1H), 4.45 - 4.40 (m, 1H), 4.37 - 4.30 (m, 3H), 4.19 - 4.12 (m, 2H), 3.73 (t, *J* = 5.7 Hz, 2H), 3.60 - 3.56 (m, 3H), 2.92 (dd, *J* = 14.1, 4.9 Hz, 1H), 2.65 (dd, *J* = 14.1, 4.9 Hz, 1H), 2.25 - 2.19 (m, 4H), 1.96 - 1.86 (m, 2H), 1.78 - 1.70 (m, 2H), 1.58 - 1.50 (m, 1H), 1.36 - 1.34 (m, 2H), 1.35 (d, *J* = 7.2 Hz, 3H), 0.82 (d, *J* = 6.5 Hz, 3H), 0.77 (d, *J* = 6.5 Hz, 3 H). ^{13}C NMR (151 MHz, DMSO-d_6) δ ppm 174.03, 172.98, 172.60, 172.04, 171.20, 171.00, 170.25, 168.85, 160.02, 158.15, 155.82, 144.26, 135.13, 130.10, 127.70, 114.96, 109.60, 61.56, 54.87, 54.43, 51.92, 51.76, 51.61, 49.29, 42.17, 40.35, 36.3 4, 30.16, 30.02, 27.61, 27.30, 24.11, 23.03, 21.47, 17.65. MS *m/z*: 876.2 [M-H]$^-$; HRMS m/z [M+H]$^+$: calcd. for $C_{37}H_{52}O_{14}N_9S$, 878.3349; found, 878.3347; HPLC Relative purity: 98.7%.

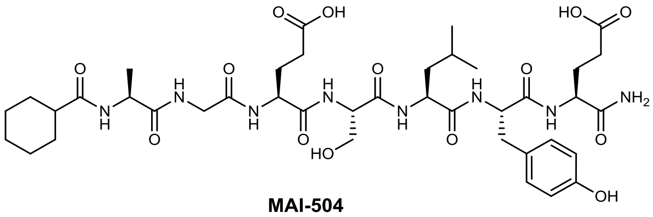

MAI-504

(4S,7S,10S,13S,16S)-4-carbamoyl-16-(2-((S)-2-(cyclohexanecarboxamido)propanamido)acetamido)-7-(4-hydroxybenzyl)-13-(hydroxymethyl)-10-isobutyl-6,9,12,15-tetraoxo-5,8,11,14-tetraazanonadecanedioic acid (MAI-504). General procedure A. White powder, 28 mg, 16% yield. ^{1}H NMR (600 MHz, DMSO-d_6) δ ppm 9.18 (s, 1H), 8.13 (t, *J* = 5.8 Hz, 1H), 8.05 (d, *J* = 7.6 Hz, 1H), 7.97 (dd, *J* = 7.0, 5.5 Hz, 2H), 7.89 (d, *J* = 7.9 Hz, 1H), 7.83 (d, *J* = 7.9 Hz, 1H), 7.80 (d, *J* = 8.0 Hz, 1H), 7.08 (q, *J* = 2.1 Hz, 2H), 7.01 (d, *J* = 8.5 Hz, 2H), 6.63 (d, *J* = 8.5 Hz, 2H), 5.13 (s, 1H), 4.41 – 4.27 (m, 3H), 4.25 – 4.05 (m, 3H), 3.70 (t, *J* = 5.4 Hz, 2H), 3.62 – 3.56 (m, 2H), 2.93 (dd, *J* = 14.1, 4.9 Hz, 1H), 2.66 (dd, *J* = 14.1, 4.9 Hz, 1H), 2. 26 – 2.12 (m, 5H), 1.98 – 1.86 (m, 2H), 1.78 – 1.66 (m, 6H), 1.61 – 1.51 (m, 2H), 1.38 – 1.25 (m, 4H), 1.21 – 1.10 (m, 6 H), 0.83 (d, *J* = 6.5 Hz, 3H), 0.78 (d, *J* = 6.5 Hz, 3H). ^{13}C NMR (151 MHz, DMSO-d_6) δ ppm 175.61, 174.00, 173.11, 172. 92, 171.98, 171.18, 170.93, 170.21, 168.92, 130.05, 127.66, 114.91, 61.52, 54.88, 54.41, 51.90, 51.59, 48.52, 43.56, 42.13, 40.29, 36.30, 30.16, 30.08, 29.15, 28.90, 27.41, 27.28, 25.43, 25.30, 25.23, 24.07, 22.98, 21.43, 17.70. MS *m/z*: 875.55 [M-H]$^-$; HRMS m/z [M+H]$^+$: calcd. for $C_{40}H_{61}O_{14}N_8$, 877.4302; found, 877.4299. HPLC Relative purity: 98.8%.

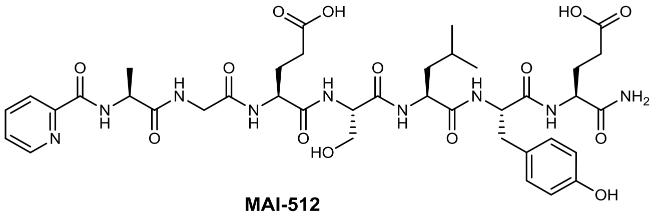

MAI-512

(4S,7S,10S,13S,16S)-4-carbamoyl-7-(4-hydroxybenzyl)-13-(hydroxymethyl)-10-isobutyl-6,9,12,15-tetraoxo-16-(2-((S)-2-(picol-inamido)propanamido)acetamido)-5,8,11,14-tetraazanonadecanedioic acid (MAI-512). General procedure A. White powder, 24 mg, 14% yield. ^{1}H NMR (500 MHz, DMSO-d6) δ 8.79 (d, *J* = 7.4 Hz, 1H), 8.69 – 8.61 (m, 1H), 8.43 (t, *J* = 5.8 Hz, 1H), 8.13 – 7.93 (m, 5H), 7.80 (dd, *J* = 12.7, 7.9 Hz, 2H), 7.66 – 7.56 (m, 1H), 7.08 (s, 2H), 7.00 (d, *J* = 8.5 Hz, 2H), 6.63 (d, *J* = 8.5 Hz, 2H), 4.62 – 4.45 (m, 1H), 4.41 – 4.24 (m, 3H), 4.21 – 4.09 (m, 2H), 3.88 – 3.69 (m, 2H), 3.61 (m, 2H), 2.93 (dd, *J* = 14.1, 4.9 Hz, 1H), 2.66 (dd, *J* = 14.1, 4.9 Hz, 1H), 2.30 – 2.12 (m, 4H), 1.98 – 1.84 (m, 2H), 1.85 – 1.68 (m, 2H), 1.53 (dp, *J* = 13.3, 6.6 Hz, 1H), 1.36 (dd, *J* = 19.4, 7.1 Hz, 5H), 0.81 (d, *J* = 6.6 Hz, 3H), 0.75 (d, *J* = 6.5 Hz, 3H). ^{13}C NMR (126 MHz, DMSO-d_6) δ ppm 174.06, 174.03, 173.00, 172.44, 172.05, 171.34, 171.00, 170.30, 168.87, 163.30, 155.84, 149.35, 148.53, 137.99, 130.11, 127.72, 126.85, 121.94, 114.98, 61.53, 55.04, 54.48, 51.95, 51.67, 48.56, 42.22, 40.33, 36.35, 30.17, 30.08, 27.58, 27.32, 24.12, 23.00, 21.45, 18.74. MS *m/z*: 870.4 [M-H]$^-$; HRMS m/z [M+H]$^+$: calcd. for $C_{39}H_{54}O_{14}N_9$, 872.3785; found, 872.3787. HPLC Relative purity: 98.5%.

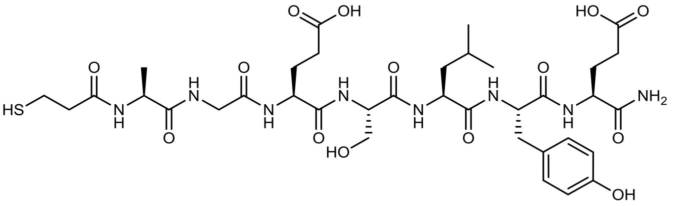

MAI-502

(4S,7S,10S,13S,16S)-4-carbamoyl-7-(4-hydroxybenzyl)-13-(hydroxymethyl)-10-isobutyl-16-(2-((S)-2-(3-mercaptopropanamido)propanamido)acetamido)-6,9,12,15-tetraoxo-5,8,11,14-tetraazanonadecanedioic acid (MAI-502). General procedure A. White powder, 20.5 mg, 12% yield. ^{1}H NMR (600 MHz, DMSO-d_6) δ 8.27 – 8.18 (m, 2H), 8.06 (d, *J* = 7.6 Hz, 1H), 7.99 (d, *J* = 7.2 Hz, 1H), 7.86 (d, *J* = 7.9 Hz, 1H), 7.84 (d, *J* = 7.9 Hz, 1H), 7.81 (d, *J* = 8.0 Hz, 1H), 7.09 (d, *J* = 5.9 Hz, 2H), 7.01 (d, *J* = 8.5 Hz, 2H), 6.63 (d, *J* = 8.5 Hz, 2H), 4.38 – 4.31 (m, 3H), 4.26 – 4.12 (m, 3H), 3.71 (d, *J* = 5.8 Hz, 2H), 3.60 – 3.56 (m, 2H), 2.93 (dd, *J* = 14.0, 4.9 Hz, 1H), 2.89 - 2.84 (m, 1H), 2.68 – 2.62 (m, 2H), 2.58 – 2.49 (m, 3H), 2.47 -2.41 (m, 1H), 2.28 (t, *J* = 8.0 Hz, 1H), 2.24 – 2.20 (m, 4H), 1.98 – 1.86 (m, 2H), 1.78 - 1.72 (m, 2H), 1.59 – 1.50 (m, 1H), 1.36 (t, *J* = 7.3Hz, 2H), 1.22 (d, *J* = 7.1 Hz, 3H), 0.83 (d, *J* = 6.6 Hz, 3H), 0.78 (d, *J* = 6.6 Hz, 3H). ^{13}C NMR (151 MHz, DMSO) δ 174.04, 174.00, 172.95, 172.01, 171.19, 170.97, 170.63, 170.24, 168.87, 155.81, 130.08, 127.68, 114.94, 61.54, 54.85, 54.42, 51.91, 51.78, 51.60, 48.64, 42.12, 40.33, 36.33, 30.14, 30.05, 27.52, 27.29, 24.09, 23.01, 21.44, 19.88, 17.91, 17.82. MS *m/z*: 853.55 [M-H]$^-$; HRMS m/z [M+H]$^+$: calcd. for $C_{36}H_{55}O_{14}N_8S$, 855.3553; found, 855.3550. HPLC Relative purity: 98.6%.

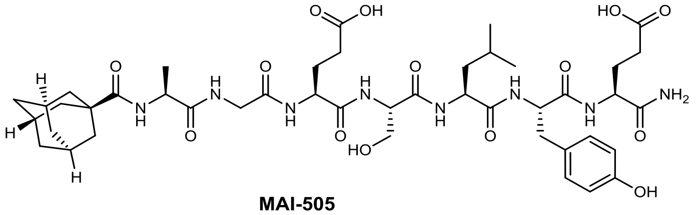

MAI-505

(4S,7S,10S,13S,16S)-16-(2-((S)-2-((3S,5S,7S)-adamantane-1-carboxamido)propanamido)acetamido)-4-carbamoyl-7-(4-hydroxybenzyl)-13-(hydroxymethyl)-10-isobutyl-6,9,12,15-tetraoxo-5,8,11,14-tetraazanonadecanedioic acid (MAI-505). General procedure A. White powder, 28 mg, 15% yield. ^{1}H NMR (600 MHz, DMSO-d_6) δ 8.07 – 8.02 (m, 2H), 7.96 (d, *J* = 7.4 Hz, 1H), 7.90 (d, *J* = 7.9 Hz, 1H), 7.83 (d, *J* = 7.9 Hz, 1H), 7.80 (d, *J* = 8.0 Hz, 1H), 7.40 (d, *J* = 6.6 Hz, 1H), 7.08 (d, *J* = 3.0 Hz, 2H), 7.01 (d, *J* = 8.5 Hz, 2H), 6.63 (d, *J* = 8.5 Hz, 2H), 4.38 – 4.31 (m, 3H), 4.23 – 4.11 (m, 3H), 3.76 – 3.67 (m, 4H), 3.63 – 3.55 (m, 2H), 2.93 (dd, *J* = 14.0, 4.9 Hz, 1H), 2.66 (dd, *J* = 14.0, 4.9 Hz, 1H), 2.27 – 2.15 (m, 4H), 1.99 – 1.87 (m, 5H), 1.80 – 1.72 (m, 8H), 1.69 – 1.61 (m, 6H), 1.55 (m, 1H), 1.36 (t, *J* = 7.5 Hz, 2H), 1.23 (d, *J* = 7.1 Hz, 3H), 0.83 (d, *J* = 6.6 Hz, 3H), 0.78 (d, *J* = 6.6 Hz, 3H). ^{13}C NMR (151 MHz, DMSO) δ 177.19, 173.97, 173.13, 172.91, 171.98, 171.17, 170.94, 170.20, 168.89, 155.79, 130.06, 127.67, 114.92, 61.54, 54.87, 54.40, 51.88, 51.83, 51.58, 48.68, 42.17, 40.31, 38.42, 36.11, 30.12, 30.07, 27.64, 27.44, 27.27, 24.08, 22.99. MS *m/z*: 929.5 [M+H]$^+$; HRMS m/z [M+H]$^+$: calcd. for $C_{44}H_{65}O_{14}N_8$, 929.4615; found, 929.4611. HPLC Relative purity: 98.7%.

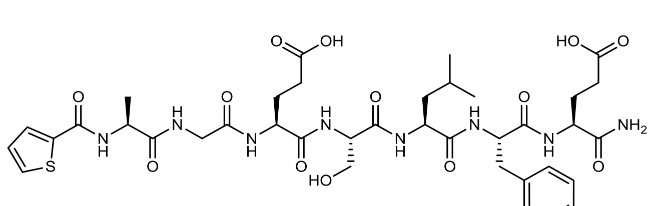

MAI-507

(4S,7S,10S,13S,16S)-4-carbamoyl-7-(4-hydroxybenzyl)-13-(hydroxymethyl)-10-isobutyl-6,9,12,15-tetraoxo-16-(2-((S)-2-(thiophene-2-carboxamido)propanamido)acetamido)-5,8,11,14-tetraazanonadecanedioic acid (MAI-507). General procedure A. White powder, 22 mg, 12.5% yield. ^{1}H NMR (600 MHz, DMSO-d_6) δ 8.63 (d, *J* = 6.9 Hz, 1H), 8.27 (t, *J* = 5.8 Hz, 1H), 8.05 (d, *J* = 7.6 Hz, 1H), 7.98 (d, *J* = 7.5 Hz, 1H), 7.91 – 7.88 (m, 2H), 7.83 (d, *J* = 7.9 Hz, 1H), 7.80 (d, *J* = 8.0 Hz, 1H), 7.75 (dd, *J* = 5.1, 1.1 Hz, 1H), 7.15 (dd, *J* = 5.0, 3.7 Hz, 1H), 7.08 (d, *J* = 4.2 Hz, 2H), 7.00 (d, *J* = 8.5 Hz, 2H), 6.63 (d, *J* = 8.5 Hz, 2H), 4.43 – 4.31 (m, 1H), 4.38 – 4.29 (m, 3H), 4.19 – 4.12 (m, 2H), 3.75 – 3.70 (m, 2H), 3.60 – 3.55 (m, 2H), 2.94 - 2.91 (dd, *J* = 14.1, 4.9 Hz, 1H), 2.66 (dd, *J* = 14.1, 4.9 Hz, 1H), 2.28 – 2.14 (m, 4H), 1.95 – 1.91 (dm, 2H), 1.81 – 1.69 (m, 2H), 1.58 – 1.48 (m, 1H), 1.39 – 1.29 (m, 5H), 0.82 (d, *J* = 6.6 Hz, 3H), 0.77 (d, *J* = 6.6 Hz, 3H). ^{13}C NMR (151 MHz, DMSO) δ 174.01, 172.94, 172.84, 172.01, 171.19, 170.97, 170.23, 168.87, 161.28, 155.80,

139.38, 131.13, 130.08, 128.90, 127.94, 127.69, 114.94, 61.55, 54.86, 54.41, 51.90, 51.77, 51.59, 49.19, 42.17, 40.33, 36.33, 30.13, 30.00, 27.53, 27.28, 24.09, 23.01, 21.44, 17.68. MS *m/z*: 875.25 [M-H]$^-$; HRMS m/z [M+H]$^+$: calcd. for $C_{38}H_{53}O_{14}N_8S$, 877.3396; found, 877.3396. HPLC Relative purity: 98.4%.

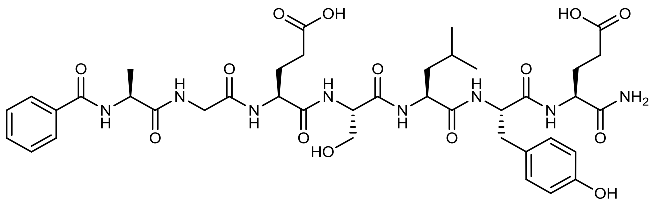

MAI-511

(4S,7S,10S,13S,16S)-16-(2-((S)-2-benzamidopropanamido)acetamido)-4-carbamoyl-7-(4-hydroxybenzyl)-13-(hydroxymethyl)-10-isobutyl-6,9,12,15-tetraoxo-5,8,11,14-tetraazanonadecanedioic acid (MAI-511). General procedure A. White powder, 22 mg, 12.6% yield. ^{1}H NMR (600 MHz, DMSO-d_6) δ 9.17 (s, 1H), 8.63 (d, *J* = 6.7 Hz, 1H), 8.28 (t, *J* = 5.8 Hz, 1H), 8.06 (d, *J* = 7.6 Hz, 1H), 7.99 (d, *J* = 7.4 Hz, 1H), 7.93 – 7.88 (m, 3H), 7.84 (d, *J* = 7.9 Hz, 1H), 7.81 (d, *J* = 8.0 Hz, 1H), 7.56-7.52 (m, 1H), 7.46 (dd, *J* = 8.3, 7.0 Hz, 2H), 7.09 (m, 2H), 7.01 (d, *J* = 8.5 Hz, 2H), 6.64 (d, *J* = 8.5 Hz, 2H), 5.13 (s, 1H), 4.46 – 4.42 (m, 1H), 4.38 – 4.30 (m, 3H), 4.20 – 4.14 (m, 2H), 3.73 (d, *J* = 5.8 Hz, 2H), 3.59 (s, 2H), 2.93 (dd, *J* = 14.1, 4.9 Hz, 1H), 2.67 (dd, *J* = 14.1, 4.9 Hz, 1H), 2.30 – 2.15 (m, 4H), 1.97 – 1.87 (m, 2H), 1.82 – 1.72 (m, 2H), 1.60 – 1.52 (m, 1H), 1.40 – 1.34 (m, 5H), 0.83 (d, *J* = 6.6 Hz, 3H), 0.78 (d, *J* = 6.6 Hz, 3H). ^{13}C NMR (151 MHz, DMSO) δ 174.00, 173.98, 173.00, 172.92, 171.99, 171.18, 170.95, 170.22, 168.93, 166.50, 155.79, 133.80, 131.42, 130.07, 128.19, 127.67, 127.62, 114.92, 61.54, 54.86, 54.40, 51.88, 51.79, 51.58, 49.46, 42.22, 40.32, 36.32, 30.13, 30.02, 27.50, 27.28, 24.08, 22.99, 21.43, 17.56. MS *m/z*: 869.30 [M-H]$^-$; HRMS m/z [M+H]$^+$: calcd. for $C_{40}H_{55}O_{14}N_8$, 871.3832; found, 871.3831. HPLC Relative purity: 98.8%.

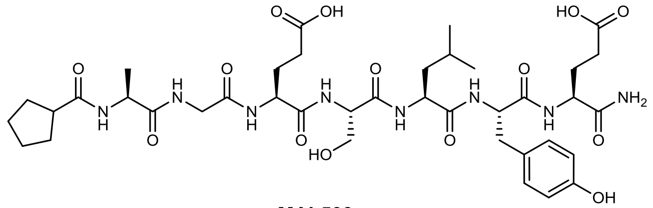

MAI-503

(4S,7S,10S,13S,16S)-4-carbamoyl-16-(2-((S)-2-(cyclopentanecarboxamido)propanamido)acetamido)-7-(4-hydroxybenzyl)-13-(hydroxymethyl)-10-isobutyl-6,9,12,15-tetraoxo-5,8,11,14-tetraazanonadecanedioic acid (MAI-503). General procedure A. White powder, 21 mg, 12% yield. ^{1}H NMR (500 MHz, DMSO-d_6) δ 8.03 (t, *J* = 5.7 Hz, 1H), 7.92 (d, *J* = 7.6 Hz, 1H), 7.89 (d, *J* = 6.7 Hz, 1H), 7.83 (dd, *J* = 9.6, 7.7 Hz, 2H), 7.74 (d, *J* = 7.9 Hz, 1H), 7.69 (d, *J* = 8.1 Hz, 1H), 7.06 – 6.94 (m, 4H), 6.63 (d, *J* = 8.5 Hz, 2H), 4.44 – 4.26 (m, 3H), 4.26 – 4.08 (m, 3H), 3.72 (d, *J* = 5.7 Hz, 2H), 3.67 – 3.52 (m, 2H), 2.94 (dd, *J* = 14.1, 5.1 Hz, 1H), 2.78 – 2.56 (m, 2H), 2.31 – 2.12 (m, 4H), 2.03 – 1.86 (m, 2H), 1.83 – 1.69 (m, 3H), 1.67 – 1.52 (m, 4H), 1.52 – 1.43 (m, 2H), 1.38 (t, *J* = 7.3 Hz, 2H), 1.22 (d, *J* = 7.1 Hz, 3H), 0.84 (d, *J* = 6.6 Hz, 3H), 0.79 (d, *J* = 6.6 Hz, 3H). ^{13}C NMR (126 MHz, DMSO) δ 175.52, 173.69, 172.69, 171.76, 171.03, 170.73, 170.03, 168.80, 155.66, 129.84, 127.54, 114.83, 61.42, 54.81, 54.31, 51.82, 51.58, 48.54, 43.82, 42.12, 40.20, 30.01, 29.92, 29.86, 29.62, 27.24, 27.16, 25.49, 23.97, 22.75, 21.36, 17.59. MS *m/z*: 861.5 [M-H]$^-$; HRMS m/z [M+H]$^+$: calcd. for $C_{39}H_{59}O_{14}N_8$, 863.4145; found, 863.4149. HPLC Relative purity: 98.2%.

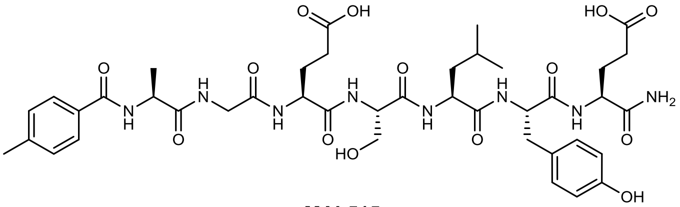

MAI-515

(4S,7S,10S,13S,16S)-4-carbamoyl-7-(4-hydroxybenzyl)-13-(hydroxymethyl)-10-isobutyl-16-(2-((S)-2-(4-methylbenzamido)propanamido)acetamido)-6,9,12,15-tetraoxo-5,8,11,14-tetraazanonadecanedioic acid (MAI-515). General procedure A. White powder, 21 mg, 11.8% yield. ^{1}H NMR (600 MHz, DMSO-d_6) δ 9.20 (s, 1H), 8.54 (d, *J* = 6.6 Hz, 1H), 8.28 (t, *J* = 5.8 Hz, 1H), 8.05 (d, *J*

= 7.6 Hz, 1H), 7.98 (d, *J* = 7.4 Hz, 1H), 7.90 (d, *J* = 7.8 Hz, 1H), 7.86 – 7.78 (m, 4H), 7.26 (d, *J* = 8.0 Hz, 2H), 7.08 (d, *J* = 5.8 Hz, 2H), 7.00 (d, *J* = 8.4 Hz, 2H), 6.63 (d, *J* = 8.4 Hz, 2H), 5.15 (s, 1H), 4.42 – 4.39 (m, 1H), 4.37 – 4.29 (m, 3H), 4.18 - 4.12 (m, 2H), 3.81 – 3.62 (m, 2H), 3.62 – 3.57 (m, 2H), 2.92 (dd, *J* = 14.1, 4.9 Hz, 1H), 2.66 (dd, *J* = 14.1, 4.9 Hz, 1H), 2.35 (s, 3H), 2.23 – 2.16 (m, 4H), 1.95 – 1.87 (m, 2H), 1.82 – 1.70 (m, 2H), 1.57 – 1.50 (m, 1H), 1.39 – 1.29 (m, 5H), 0.82 (d, *J* = 6.6 Hz, 3H), 0.77 (d, *J* = 6.6 Hz, 3H). ^{13}C NMR (151 MHz, DMSO) δ 174.06, 173.18, 173.01, 172.06, 171.27, 171.01, 170.30, 169.05, 166.48, 155.82, 141.38, 131.00, 130.11, 128.77, 127.71, 127.69, 114.97, 61.55, 54.94, 54.48, 51.91, 51.67, 49.51, 42.27, 36.34, 30.19, 30.06, 27.46, 27.29, 24.12, 23.02. MS *m/z*: 883.7 [M-H]$^-$; HRMS m/z [M+H]$^+$: calcd. for $C_{41}H_{57}O_{14}N_8$, 885.3989; found, 885.3987. HPLC Relative purity: 98.9%. HPLC purity yield: 21 mg, 13.9%.

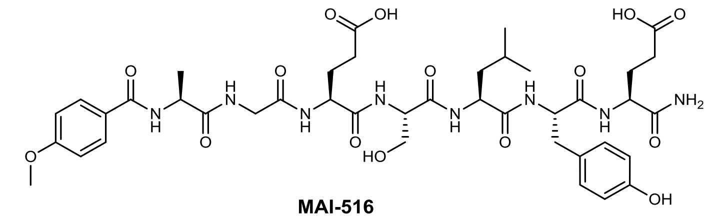

MAI-516

(4S,7S,10S,13S,16S)-4-carbamoyl-7-(4-hydroxybenzyl)-13-(hydroxymethyl)-10-isobutyl-16-(2-((S)-2-(4-methoxybenzamido) propanamido)acetamido)-6,9,12,15-tetraoxo-5,8,11,14-tetraazanonadecanedioic acid (MAI-516). General procedure A. White powder, 23 mg, 12.7% yield. ^{1}H NMR (600 MHz, DMSO-d_6) δ 9.19 (s, 1H), 8.49 (d, *J* = 7.5 Hz, 1H), 8.29 (t, *J* = 5.8 Hz, 1H), 8.05 (d, *J* = 7.5 Hz, 1H), 7.98 (d, *J* = 7.3 Hz, 1H), 7.92 – 7.87 (m, 3H), 7.83 (d, *J* = 7.9 Hz, 1H), 7.79 (d, *J* = 8.0 Hz, 1H), 7.08 (s, 2H), 7.02 – 6.96 (m, 4H), 6.63 (dd, *J* = 9.0, 2.5 Hz, 2H), 5.15 (s, 1H), 4.49 – 4.27 (m, 4H), 4.18 – 4.14 (m, 2H), 3.80 (s, 3H), 3.77 – 3.68 (m, 2H), 3.62 – 3.56 (m, 2H), 2.93 (dd, *J* = 14.0, 4.9 Hz, 1H), 2.66 (dd, *J* = 14.0, 4.9 Hz, 1H), 2.27 – 2.17 (m, 4H), 1.95 – 1.90 (m, 2H), 1.82 – 1.71 (m, 2H), 1.58 – 1.52 (m, 1H), 1.38 – 1.34 (m, 5H), 0.82 (d, *J* = 6.6 Hz, 3H), 0.77 (d, *J* = 6.6 Hz, 3H). ^{13}C NMR (151 MHz, DMSO) δ 174.05, 173.27, 172.99, 172.05, 171.27, 170.99, 170.30, 169.07, 166.11, 161.78, 155.82, 130.10, 129.56, 127.71, 125.99, 114.96, 113.41, 61.55, 55.41, 54.95, 54.48, 51.94, 51.67, 49.55, 42.29, 36.32, 30.19, 30.07, 27.43, 27.29, 24.11, 23.01, 21.46, 17.55. MS *m/z*: 899.4 [M-H]$^-$; HRMS m/z [M+H]$^+$: calcd. for $C_{41}H_{57}O_{15}N_8$, 901.3938; found, 901.3936. HPLC Relative purity: 98.6%.

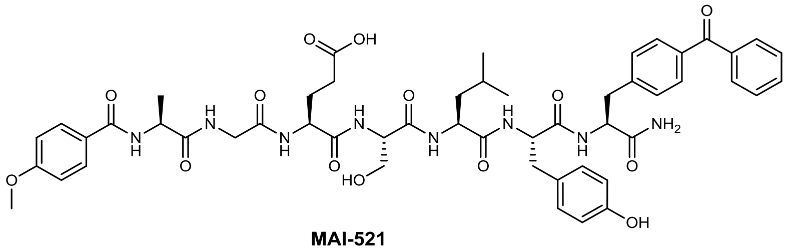

MAI-521

(2S,5S,8S,11S,14S)-1-amino-2-(4-benzoylbenzyl)-5-(4-hydroxybenzyl)-11-(hydroxymethyl)-8-isobutyl-14-(2-((S)-2-(4-methoxy-benzamido)propanamido)acetamido)-1,4,7,10,13-pentaoxo-3,6,9,12-tetraazaheptadecan-17-oic acid (MAI-521). General procedure A. White powder, 20 mg, 9.8% yield. ^{1}H NMR (600 MHz, DMSO-d_6) δ 9.19 (s, 1H), 8.49 (d, *J* = 6.4 Hz, 1H), 8.29 (t, *J* = 5.9 Hz, 1H), 8.06 (d, *J* = 7.5 Hz, 1H), 8.00 (d, *J* = 7.2 Hz, 1H), 7.94 – 7.86 (m, 4H), 7.78 (d, *J* = 7.8 Hz, 1H), 7.71 – 7.67 (m, 2H), 7.65 (d, *J* = 7.5 Hz, 3H), 7.52 (t, *J* = 7.8 Hz, 2H), 7.41 (d, *J* = 7.5 Hz, 2H), 7.29 – 7.23 (m, 2H), 7.9 – 7.16 (m, 2H), 6.98 – 6.93 (m, 4H), 6.62 – 6.59 (d, *J* = 7.5 Hz, 2H), 5.21 (t, *J* = 4.7 Hz, 1H), 4.48 – 4.44 (m, 1H), 4.39 – 4.37 (m, 1H), 4.36 – 4.37 (m, 4H), 4.17 – 4.12 (m, 1H), 3.80 (s, 3H), 3.72 (t, *J* = 6.0 Hz, 2H), 3.64 – 3.56 (m, 4H), 3.14 (dd, *J* = 13.9, 5.1 Hz, 1H), 2.94 (dd, *J* = 13.9, 5.1 Hz, 1H), 2.81(dd, *J* = 13.9, 9.3 Hz, 1H), 2.60 (dd, *J* = 13.9, 9.3 Hz, 1H), 2.22 (t, *J* = 8.9, Hz, 2H), 1.96 – 1.89 (m, 1H), 1.83 – 1.74 (m, 1H), 1.56 – 1.52 (m, 1H), 1.36 – 1.30 (m, 5H), 0.78 (d, *J* = 6.7 Hz, 3H), 0.73 (d, *J* = 6.7 Hz, 3H). ^{13}C NMR (151 MHz, DMSO) δ 195.59, 173.98, 173.96, 173.28, 172.37, 172.00, 171.34, 170.86, 170.43, 169.13, 166.12, 161.77, 158.33, 158.12, 157.92, 157.71, 155.80, 143.24, 137.26, 135.09, 132.56, 130.03, 129.68, 129.54, 129.46, 129.18, 128.83, 128.55, 127.99, 127.73, 127.69, 127.64, 127.55, 127.13, 125.96, 114.92, 113.39, 61.49, 55.39, 55.06, 54.70, 53.58, 51.98, 51.75, 49.57, 42.30, 37.48, 36.41, 34.32, 30.05, 27.35, 24.11, 24.08, 23.06, 22.97, 21.42, 21.35, 17.52. MS *m/z*: 1021.35 [M-H]$^-$; HRMS m/z [M+H]$^+$: calcd. for $C_{52}H_{63}O_{14}N_8$, 1023.4458; found, 1023.4458. HPLC Relative purity: 98.1%.

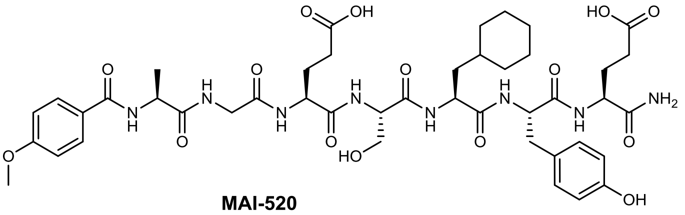

MAI-520

(4S,7S,10S,13S,16S)-4-carbamoyl-10-(cyclohexylmethyl)-7-(4-hydroxybenzyl)-13-(hydroxymethyl)-16-(2-((S)-2-(4-methoxybenzamido)propanamido)acetamido)-6,9,12,15-tetraoxo-5,8,11,14-tetraazanonadecanedioic acid (MAI-520). General procedure A. White powder, 25 mg, 13.2% yield. ^{1}H NMR (600 MHz, DMSO-d_6) δ 9.16 (s, 1H), 8.48 (d, *J* = 6.5 Hz, 1H), 8.27 (t, *J* = 5.8 Hz, 1H), 8.04 (d, *J* = 7.6 Hz, 1H), 7.96 (d, *J* = 7.5 Hz, 1H), 7.91 – 7.86 (m, 3H), 7.80 (dd, *J* = 8.0, 5.9 Hz, 2H), 7.08 (s, 2H), 7.02 – 6.96 (m, 4H), 6.62 (d, *J* = 8.5 Hz, 2H), 5.11 (s, 1H), 4.41 – 4.30 (m, 4H), 4.22 – 4.18 (m, 1H), 4.16 – 4.12 (m, 1H), 3.80 (s, 3H), 3.72 (dd, *J* = 5.7, 1.8 Hz, 2H), 3.63 – 3.54 (m, 2H), 2.92 (dd, *J* = 14.1, 4.9 Hz, 1H), 2.65 (dd, *J* = 14.1, 4.9 Hz, 1H), 2.26 – 2.14 (m, 4H), 1.93 – 1.90 (m, 2H), 1.82 – 1.71 (m, 2H), 1.62 – 1.59 (m, 5H), 1.40 – 1.30 (m, 5H), 1.27 – 1.19 (m, 1H), 1.19 – 1.02 (m, 3H), 0.88 – 0.69 (m, 2H). ^{13}C NMR (151 MHz, DMSO) δ 174.00, 173.24, 172.93, 172.09, 171.17, 170.96, 170.21, 168.99, 166.08, 161.76, 155.80, 130.07, 129.53, 127.68, 125.97, 114.92, 113.38, 61.62, 55.38, 54.87, 54.42, 51.90, 50.92, 49.52, 42.27, 36.33, 33.27, 33.16, 31.71, 30.16, 30.05, 27.46, 27.28, 26.11, 25.76, 25.51, 17.53. MS *m/z*: 939.4 [M-H]$^-$; HRMS m/z [M+H]$^+$: calcd. for $C_{44}H_{61}O_{15}N_8$, 941.4251; found, 941.4249. HPLC Relative purity: 98.4%.

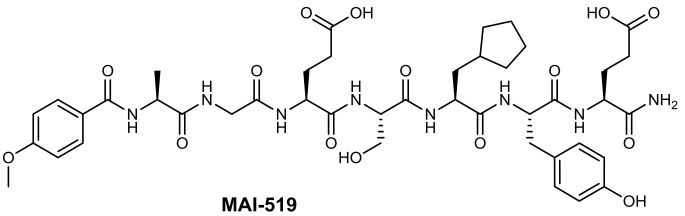

MAI-519

(4S,7S,10S,13S,16S)-4-carbamoyl-10-(cyclopentylmethyl)-7-(4-hydroxybenzyl)-13-(hydroxymethyl)-16-(2-((S)-2-(4-methoxybenzamido)propanamido)acetamido)-6,9,12,15-tetraoxo-5,8,11,14-tetraazanonadecanedioic acid (MAI-519). General procedure A. White powder, 25 mg, 13.5% yield. ^{1}H NMR (600 MHz, DMSO-d_6) δ 9.16 (s, 1H), 8.47 (d, *J* = 6.5 Hz, 1H), 8.26 (t, *J* = 5.8 Hz, 1H), 8.04 (d, *J* = 7.6 Hz, 1H), 7.96 (d, *J* = 7.5 Hz, 1H), 7.92 – 7.82 (m, 4H), 7.79 (d, *J* = 8.0 Hz, 1H), 7.08 (s, 2H), 7.03 – 6.94 (m, 4H), 6.63 (d, *J* = 8.5 Hz, 2H), 5.10 (s, 1H), 4.42 – 4.29 (m, 4H), 4.16 – 4.12 (m, 2H), 3.80 (s, 3H), 3.72 (d, *J* = 5.9 Hz, 2H), 3.62 – 3.56 (m, 2H), 2.92 (dd, *J* = 14.1, 4.9 Hz, 1H), 2.66 (dd, *J* = 14.1, 4.9 Hz, 1H), 2.26 – 2.17 (m, 4H), 1.97 – 1.87 (m, 2H), 1.82 – 1.69 (m, 3H), 1.68 – 1.60 (m, 2H), 1.55 – 1.47 (m, 4H), 1.44 – 1.37 (m, 2H), 1.34 (d, *J* = 7.2 Hz, 3H), 1.06 – 0.96 (m, 2H). ^{13}C NMR (151 MHz, DMSO) δ 174.00, 173.98, 173.22, 172.92, 171.84, 171.18, 170.96, 170.18, 168.96, 166.06, 161.75, 155.80, 130.06, 129.52, 127.69, 125.98, 114.92, 113.38, 61.57, 55.38, 54.85, 54.45, 52.79, 51.89, 51.83, 49.50, 42.24, 37.69, 36.32, 35.99, 32.34, 31.65, 30.13, 30.04, 27.46, 27.28, 24.80, 24.61, 17.53. MS *m/z*: 925.3 [M-H]$^-$; HRMS m/z [M+H]$^+$: calcd. for $C_{43}H_{59}O_{15}N_8$, 927.4094; found, 927.4091. HPLC Relative purity: 98.1%.

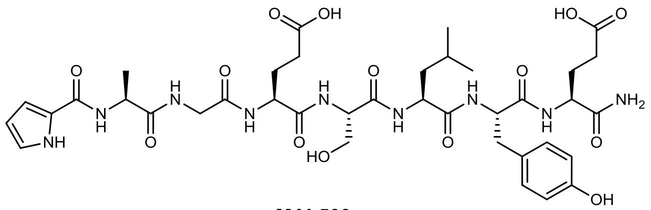

MAI-509

(4S,7S,10S,13S,16S)-16-(2-((S)-2-(1H-pyrrole-2-carboxamido)propanamido)acetamido)-4-carbamoyl-7-(4-hydroxybenzyl)-13-(hydroxymethyl)-10-isobutyl-6,9,12,15-tetraoxo-5,8,11,14-tetraazanonadecanedioic acid (MAI-509). General procedure A. White powder, 20 mg, 11.6% yield. ^{1}H NMR (500 MHz, DMSO-d_6) δ 11.3 (s, 1H), 8.17 (t, *J* = 5.7 Hz, 1H), 8.10 (d, *J* = 7.1 Hz, 1H), 8.05 (d, *J* = 7.7 Hz, 1H), 8.00 (d, *J* = 7.4 Hz, 1H), 7.92 (d, *J* = 7.8 Hz, 1H), 7.82 (dd, *J* = 15.9, 7.9 Hz, 2H), 7.08 (d, *J* = 5.1 Hz, 2H), 7.00 (d, *J* = 8.4 Hz, 2H), 6.87 (m, 2H), 6.63 (d, *J* = 8.4 Hz, 2H), 6.09 (dt, *J* = 3.6, 2.4 Hz, 1H), 4.41 (t, *J* = 7.1 Hz, 1H), 4.39 – 4.29 (m, 3H), 4.21 – 4.12 (m, 2H), 3.77 – 3.68 (m, 2H), 3.62 – 3.55 (m, 2H), 2.92 (dd, *J* = 14.1, 4.9 Hz, 1H), 2.66 (dd, *J* = 14.1, 4.9 Hz, 1H), 2.27 – 2.17 (m, 4H), 1.95 – 1.86 (m, 2H), 1.77 – 1.73 (m, 2H), 1.57 -1.51 (m, 2H), 1.35 (t, *J* = 7.4 Hz, 2H), 1.31 (d, *J* = 7.2 Hz, 3H), 0.82 (d, *J* = 6.6 Hz,

3H), 0.77 (d, *J* = 6.6 Hz, 3H). ^{13}C NMR (126 MHz, DMSO) δ 173.95, 173.13, 172.89, 171.96, 171.15, 170.93, 170.18, 168.83, 160.53, 155.78, 130.05, 127.66, 125.76, 121.63, 117.77, 114.91, 111.05, 108.68, 61.52, 54.87, 54.37, 51.87, 51.76, 51.55, 48.54, 42.14, 40.33, 36.51, 36.31, 30.11, 29.96, 27.58, 27.27, 24.06, 22.98, 21.42, 17.85, 16.84. MS *m/z*: 860.35 $[M+H]^+$; HRMS m/z $[M+H]^+$: calcd. for $C_{38}H_{54}O_{14}N_9$, 860.3785; found, 860.3782. HPLC Relative purity: 98.5%.

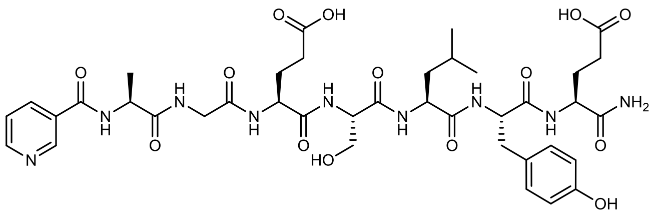

MAI-513

(4S,7S,10S,13S,16S)-4-carbamoyl-7-(4-hydroxybenzyl)-13-(hydroxymethyl)-10-isobutyl-16-(2-((S)-2-(nicotinamido)propanamido)acetamido)-6,9,12,15-tetraoxo-5,8,11,14-tetraazanonadecanedioic acid (MAI-513). General procedure A. White powder, 22 mg, 12.6% yield. ^{1}H NMR (500 MHz, DMSO-d_6) δ 9.12 (d, *J* = 2.1 Hz, 1H), 8.96 (d, *J* = 6.8 Hz, 1H), 8.79 (d, *J* = 4.5 Hz, 1H), 8.40 (d, *J* = 8.0 Hz, 1H), 8.35 (t, *J* = 5.8 Hz, 1H), 8.07 (d, *J* = 7.6 Hz, 1H), 8.01 (d, *J* = 7.5 Hz, 1H), 7.88 (d, *J* = 7.9 Hz, 1H), 7.83 (dd, *J* = 16.6, 7.9 Hz, 2H), 7.65 (dd, *J* = 8.0, 5.0 Hz, 1H), 7.09 (d, *J* = 6.9 Hz, 2H), 7.01 (d, *J* = 8.4 Hz, 2H), 6.63 (d, *J* = 8.4 Hz, 2H), 4.50 – 4.44 (m, 2H), 4.39 – 4.31 (m, 5H), 4.21 – 4.13 (m, 2H), 3.74 (d, *J* = 5.7 Hz, 3H), 3.59 (m, 3H), 2.93 (dd, *J* = 14.2, 4.9 Hz, 1H), 2.66 (dd, *J* = 14.2, 2.9 Hz, 1H), 2.27 – 2.16 (m, 4H), 1.96 – 1.79 (m, 2H), 1.78 – 1.74 (m, 2H), 1.58 – 1.51 (m, 1H), 1.38 – 1.35 (m, 5H), 0.83 (d, *J* = 6.6 Hz, 3H), 0.78 (d, *J* = 6.6 Hz, 3H). ^{13}C NMR (126 MHz, DMSO) δ 173.98, 172.91, 172.61, 171.97, 171.15, 170.94, 170.20, 168.83, 164.39, 158.45, 158.16, 157.87, 155.79, 150.28, 147.25, 137.33, 130.05, 127.66, 124.12, 116.83, 114.92, 114.51, 61.53, 54.84, 54.39, 51.88, 51.73, 51.57, 49.45, 42.17, 40.33, 30.12, 29.99, 27.59, 27.27, 24.07, 22.98, 21.43, 17.55. MS *m/z*: 870.45 $[M-H]^-$; HRMS m/z $[M+H]^+$: calcd. for $C_{39}H_{54}O_{14}N_9$, 872.3785; found, 872.3782. HPLC Relative purity: 98.1%.

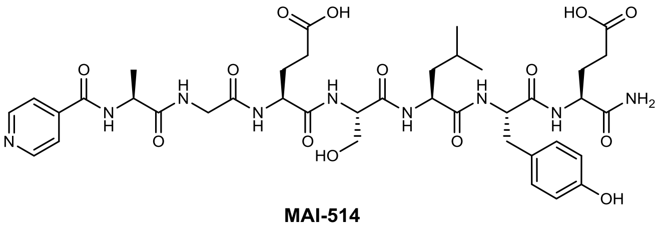

MAI-514

(4S,7S,10S,13S,16S)-4-carbamoyl-7-(4-hydroxybenzyl)-13-(hydroxymethyl)-10-isobutyl-16-(2-((S)-2-(isonicotinamido)propanamido)acetamido)-6,9,12,15-tetraoxo-5,8,11,14-tetraazanonadecanedioic acid (MAI-514). General procedure A. White powder, 25 mg, 14.3% yield. ^{1}H NMR (600 MHz, DMSO-d_6) δ 9.07 (d, *J* = 6.8 Hz, 1H), 8.86 – 8.80 (m, 2H), 8.36 (t, *J* = 5.8 Hz, 1H), 8.07 (d, *J* = 7.7 Hz, 1H), 8.02 (d, *J* = 7.7 Hz, 1H), 8.00 – 7.96 (m, 2H), 7.89 – 7.80 (m, 3H), 7.12 – 7.07 (m, 2H), 7.03 – 6.99 (m, 2H), 6.66 – 6.61 (m, 2H), 4.50 – 4.44 (m, 1H), 4.39 – 4.31 (m, 3H), 4.20 – 4.14 (m, 2H), 3.74 (d, *J* = 5.8 Hz, 2H), 3.61 – 3.56 (m, 2H), 2.93 (dd, *J* = 14.1, 4.9 Hz, 1H), 2.67 (dd, *J* = 14.1, 4.9 Hz, 1H), 2.28 – 2.16 (m, 4H), 1.97 – 1.87 (m, 2H), 1.80 – 1.71 (m, 2H), 1.59 – 1.52 (m, 1H), 1.38 – 1.35 (m, 5H), 0.83 (d, *J* = 6.6 Hz, 3H), 0.78 (d, *J* = 6.6 Hz, 3H). ^{13}C NMR (151 MHz, DMSO-d_6) δ 174.02, 173.98, 172.93, 172.44, 172.00, 171.15, 170.96, 170.21, 168.79, 164.26, 158.49, 158.25, 155.80, 148.23, 142.92, 130.07, 127.68, 122.57, 114.93, 61.56, 54.83, 54.39, 51.89, 51.72, 51.57, 49.55, 42.17, 40.35, 36.33, 30.13, 30.02, 27.64, 27.29, 24.09, 23.00, 21.44, 17.51. MS *m/z*: 872.35 $[M+H]^+$; HRMS m/z $[M+H]^+$: calcd. for $C_{39}H_{54}O_{14}N_9$, 872.3785; found, 872.3783. HPLC Relative purity: 98.2%.

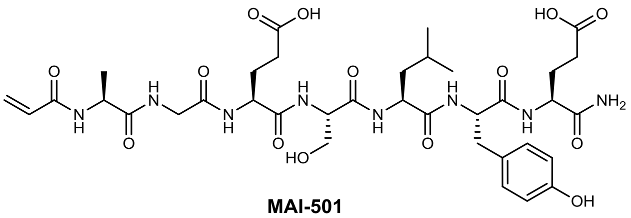

MAI-501

(4S,7S,10S,13S,16S)-16-(2-((S)-2-acrylamidopropanamido)acetamido)-4-carbamoyl-7-(4-hydroxybenzyl)-13-(hydroxymethyl)-10-isobutyl-6,9,12,15-tetraoxo-5,8,11,14-tetraazanonadecanedioic acid (MAI-501). General procedure A. White powder, 20 mg, 12% yield. ^{1}H NMR (500 MHz, DMSO-d$_6$) δ 9.19 (s, 2H), 8.41 (d, *J* = 6.7 Hz, 1H), 8.31 (t, *J* = 5.8 Hz, 1H), 8.06 (d, *J* = 7.6 Hz, 1H), 7.98 (d, *J* = 7.4 Hz, 1H), 7.90 (d, *J* = 7.8 Hz, 1H), 7.82 (dd, *J* = 17.9, 7.9 Hz, 2H), 7.13 – 7.06 (m, 2H), 7.04 – 6.98 (m, 2H), 6.68 – 6.61 (m, 2H), 6.32 (dd, *J* = 17.1, 10.2 Hz, 1H), 6.11 (dd, *J* = 17.1, 2.1 Hz, 1H), 5.60 (dd, *J* = 10.2, 2.1 Hz, 1H), 4.41 – 4.27 (m, 4H), 4.23 – 4.11 (m, 2H), 3.77 – 3.69 (m, 2H), 3.67 – 3.55 (m, 2H), 2.94 (dd, *J* = 14.1, 4.8 Hz, 1H), 2.67 (dd, *J* = 14.0, 9.3 Hz, 1H), 2.28 – 2.18 (m, 4H), 1.98 – 1.87 (m, 2H), 1.83 – 1.71 (m, 2H), 1.60 – 1.50 (m, 1H), 1.41 – 1.32 (m, 2H), 1.26 (d, *J* = 7.1 Hz, 3H), 0.84 (d, *J* = 6.6 Hz, 3H), 0.79 (d, *J* = 6.5 Hz, 3H). ^{13}C NMR (126 MHz, DMSO) δ 174.08, 174.05, 173.03, 172.88, 172.08, 171.28, 171.03, 170.32, 168.98, 164.76, 155.85, 131.31, 130.13, 127.73, 125.86, 115.00, 61.59, 54.93, 54.49, 51.97, 51.92, 51.69, 48.80, 42.21, 40.35, 36.36, 30.19, 30.06, 27.45, 27.32, 24.14, 23.04, 21.49, 17.87. MS *m/z*: 819.4 [M-H]$^-$; HRMS m/z [M+H]$^+$: calcd. for $C_{36}H_{53}O_{14}N_8$, 821.3676; found,821.3679. HPLC Relative purity: 98.1%.

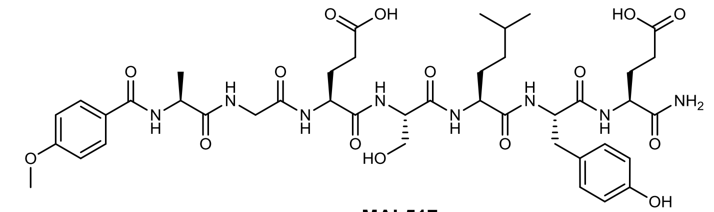

MAI-517

(4S,7S,10S,13S,16S)-4-carbamoyl-7-(4-hydroxybenzyl)-13-(hydroxymethyl)-10-isopentyl-16-(2-((S)-2-(4-methoxybenzamido)propanamido)acetamido)-6,9,12,15-tetraoxo-5,8,11,14-tetraazanonadecanedioic acid (MAI-517). General procedure A. White powder, 24 mg, 13% yield. ^{1}H NMR (600 MHz, DMSO-*d*$_6$) δ 9.16 (s, 1H), 8.47 (d, *J* = 6.5 Hz, 1H), 8.27 (t, *J* = 5.8 Hz, 1H), 8.05 (d, *J* = 7.5 Hz, 1H), 7.95 (d, *J* = 7.5 Hz, 1H), 7.91 – 7.83 (m, 4H), 7.79 (d, *J* = 8.0 Hz, 1H), 7.07 (q, *J* = 2.2 Hz, 2H), 7.02 – 6.97 (m, 4H), 6.65 – 6.61 (m, 2H), 5.14 (t, *J* = 5.5 Hz, 1H), 4.42 – 4.31 (m, 4H), 4.20 (td, *J* = 8.4, 5.2 Hz, 1H), 4.09 (td, *J* = 8.4, 5.2 Hz, 1H), 3.81 (s, 3H), 3.72 (d, *J* = 5.8 Hz, 2H), 3.62 – 3.54 (m, 2H), 2.93 (dd, *J* = 14.1, 4.8 Hz, 1H), 2.65 (dd, *J* = 14.1, 4.8 Hz, 1H), 2.29 – 2.15 (m, 4H), 1.97 – 1.88 (m, 2H), 1.81 – 1.72 (m, 2H), 1.58 – 1.52 (m, 1H), 1.47 - 1.42 (m, 2H), 1.34 (d, *J* = 7.2 Hz, 3H), 1.09 – 1.00 (m, 2H), 0.79 (dd, *J* = 6.6, 2.4 Hz, 6H). ^{13}C NMR (151 MHz, DMSO) δ 173.99, 173.22, 172.92, 171.63, 171.17, 170.98, 170.33, 168.95, 166.06, 161.75, 155.80, 130.03, 129.52, 127.67, 125.97, 114.93, 113.38, 61.64, 55.38, 54.74, 54.46, 53.40, 51.90, 51.82, 49.51, 42.24, 36.32, 33.96, 30.16, 30.06, 29.47, 27.46, 27.36, 27.30, 22.53, 22.27, 17.52. MS *m/z*: 913.4 [M-H]$^-$; HRMS m/z [M+H]$^+$: calcd. for $C_{42}H_{59}O_{15}N_8$, 915.4094; found, 915.4092. HPLC Relative purity: 98.6%.

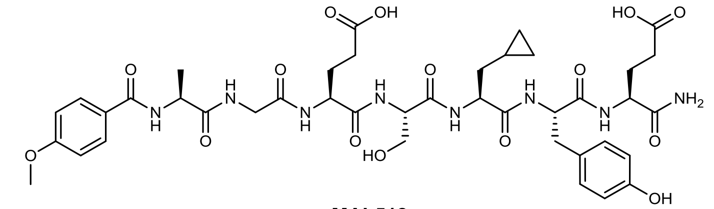

MAI-518

(4S,7S,10S,13S,16S)-4-carbamoyl-10-(cyclopropylmethyl)-7-(4-hydroxybenzyl)-13-(hydroxymethyl)-16-(2-((S)-2-(4-methoxybenzamido)propanamido)acetamido)-6,9,12,15-tetraoxo-5,8,11,14-tetraazanonadecanedioic acid (MAI-518). General procedure A. White powder, 23 mg, 12.8% yield. ^{1}H NMR (600 MHz, DMSO-*d*$_6$) δ 9.17 (s, 1H), 8.47 (d, *J* = 6.5 Hz, 1H), 8.27 (q, *J* = 5.9, 4.9 Hz, 1H), 8.06 (d, *J* = 7.4 Hz, 1H), 7.96 (d, *J* = 7.4 Hz, 1H), 7.90 – 7.87 (m, 4H), 7.80 (d, *J* = 8.0 Hz, 1H), 7.11 – 7.05 (m, 2H), 7.04 – 6.96 (m, 4H), 6.62 (d, *J* = 8.5 Hz, 2H), 5.13 (s, 1H), 4.42 – 4.31 (m, 4H), 4.23 – 4.21 (m, 1H), 4.16 – 4.12 (m, 1H), 3.80 (s, 3H), 3.72 (d, *J* = 5.8 Hz, 2H), 3.64 – 3.55 (m, 2H), 2.91 (dd, *J* = 14.0, 4.9 Hz, 1H), 2.66 (dd, *J* = 14.0, 4.9 Hz, 1H), 2.26 – 2.15 (m, 4H), 1.98 – 1.87 (m, 2H), 1.81 – 1.70 (m, 2H), 1.52 – 1.47 (m, 1H), 1.37 – 1.30 (m, 4H), 0.65 – 0.60 (m, 1H), 0.30 – 0.23 (m, 2H), 0.02 – -0.08 (m, 2H). ^{13}C NMR (151 MHz, DMSO-*d*$_6$) δ 174.01, 173.99, 173.22, 172.94, 171.46, 171.20, 170.95, 170.20, 168.98, 166.06, 161.75, 155.80, 130.08, 129.52, 127.71, 125.97, 114.94, 113.39, 61.60, 55.39, 54.84, 54.58, 53.71, 51.88, 49.49, 42.24, 36.32, 30.12, 30.03, 27.43, 27.26, 17.53, 7.50, 4.43, 3.98. MS *m/z*: 899.4 [M+H]$^+$; HRMS m/z [M+H]$^+$: calcd. for $C_{41}H_{55}O_{15}N_8$, 899.3781; found, 899.3778. HPLC Relative purity: 98.4%.

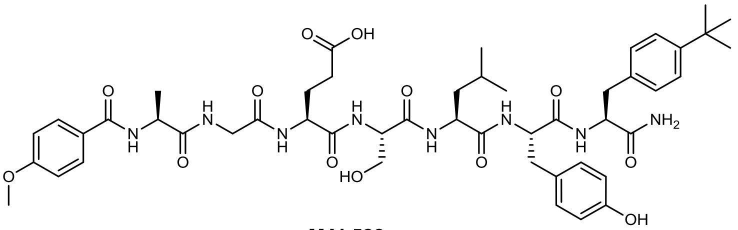

MAI-522

(2S,5S,8S,11S,14S)-1-amino-2-(4-(tert-butyl)benzyl)-5-(4-hydroxybenzyl)-11-(hydroxymethyl)-8-isobutyl-14-(2-((S)-2-(4-methoxybenzamido)propanamido)acetamido)-1,4,7,10,13-pentaoxo-3,6,9,12-tetraazaheptadecan-17-oic acid (MAI-522). General procedure A. White powder, 26 mg, 13.3% yield. ^{1}H NMR (600 MHz, DMSO-d_6) δ 9.15 (s, 1H), 8.48 (d, *J* = 6.5 Hz, 1H), 8.27 (t, *J* = 5.8 Hz, 1H), 8.04 (d, *J* = 7.6 Hz, 1H), 7.98 (d, *J* = 7.6 Hz, 1H), 7.92 – 7.87 (m, 3H), 7.78 (dd, *J* = 26.6, 8.0 Hz, 2H), 7.30 – 7.24 (m, 2H), 7.19 – 7.06 (m, 4H), 7.01 – 6.97 (m, 2H), 6.95 – 6.90 (m, 2H), 6.64 – 6.55 (m, 2H), 5.15 (s, 1H), 4.44 – 4.27 (m, 4H), 4.23 – 4.14 (m, 1H), 3.80 (s, 3H), 3.76 – 3.68 (m, 2H), 3.61 – 3.58 (m, 1H), 2.98 (dd, *J* = 14.0, 5.2 Hz, 1H), 2.84 – 2.74 (m, 2H), 2.64 – 2.54 (m, 1H), 2.29 – 2.17 (m, 2H), 1.98 – 1.88 (m, 1H), 1.84 – 1.74 (m, 1H), 1.59 – 1.50 (m, 1H), 1.39 – 1.31 (m, 4H), 1.24 (s, 9H), 0.82 (d, *J* = 6.6 Hz, 3H), 0.77 (d, *J* = 6.6 Hz, 3H). ^{13}C NMR (151 MHz, DMSO) δ 173.97, 173.25, 172.66, 171.91, 171.30, 170.78, 170.33, 169.07, 166.07, 161.75, 155.73, 148.45, 130.04, 129.52, 128.85, 127.64, 125.95, 124.85, 114.86, 113.37, 55.37, 49.52, 34.09, 31.22, 30.03, 27.37, 24.08, 21.38, 17.51. MS *m/z*: 973.4 [M-H]$^-$; HRMS m/z [M+H]$^+$: calcd. for $C_{49}H_{67}O_{13}N_8$, 975.4822; found, 975.4819. HPLC Relative purity: 98.3%.

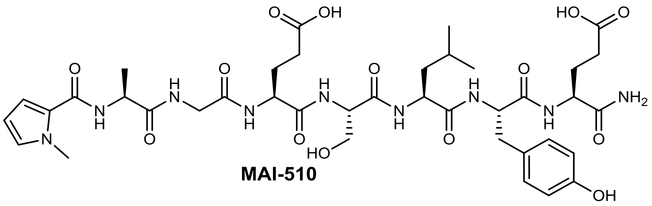

MAI-510

(4S,7S,10S,13S,16S)-4-carbamoyl-7-(4-hydroxybenzyl)-13-(hydroxymethyl)-10-isobutyl-16-(2-((S)-2-(1-methyl-1H-pyrrole-2-carboxamido)propanamido)acetamido)-6,9,12,15-tetraoxo-5,8,11,14-tetraazanonadecanedioic acid (MAI-510). General procedure A. White powder, 21 mg, 12% yield. ^{1}H NMR (500 MHz, DMSO-d_6) δ 8.11 (t, *J* = 5.8 Hz, 1H), 8.01 (t, *J* = 7.4 Hz, 2H), 7.94 (d, *J* = 7.5 Hz, 1H), 7.87 (d, *J* = 7.9 Hz, 1H), 7.80 (d, *J* = 7.9 Hz, 1H), 7.76 (d, *J* = 8.0 Hz, 1H), 7.04 (d, *J* = 6.1 Hz, 2H), 7.00 (d, *J* = 8.2 Hz, 2H), 6.89 (d, *J* = 3.2 Hz, 2H), 6.63 (d, *J* = 8.2 Hz, 2H), 6.01 (t, *J* = 3.2 Hz, 1H), 4.39 – 4.28 (m, 4H), 4.21 – 4.11 (m, 2H), 3.81 (s, 3H), 3.73 (dd, *J* = 5.8, 2.2 Hz, 2H), 3.63 – 3.56 (m, 2H), 2.93 (dd, *J* = 14.2, 5.0 Hz, 1H), 2.67 (dd, *J* = 14.2, 5.0 Hz, 1H), 2.25 – 2.17 (m, 4H), 1.95 – 1.91 (m, 4H), 1.78 – 1.71 (m, 2H), 1.60 – 1.49 (m, 1H), 1.38 - 1.31 (m, 5H), 0.83 (d, *J* = 6.6 Hz, 3H), 0.78 (d, *J* = 6.6 Hz, 3H). ^{13}C NMR (126 MHz, DMSO) δ 173.98, 173.96, 173.15, 172.90, 172.06, 171.97, 171.14, 170.94, 170.19, 168.91, 161.34, 155.78, 130.06, 128.02, 127.66, 125.01, 114.92, 113.14, 106.67, 61.52, 54.86, 54.38, 51.87, 51.74, 51.56, 48.57, 42.23, 40.32, 36.31, 36.27, 30.12, 29.99, 27.56, 27.27, 24.07, 22.99, 21.42, 21.08, 17.68. MS *m/z*: 874.3 [M+H]$^+$; HRMS m/z [M+H]$^+$: calcd. for $C_{39}H_{56}O_{14}N_9$, 874.3941; found, 874.3938. HPLC Relative purity: 98.3%.

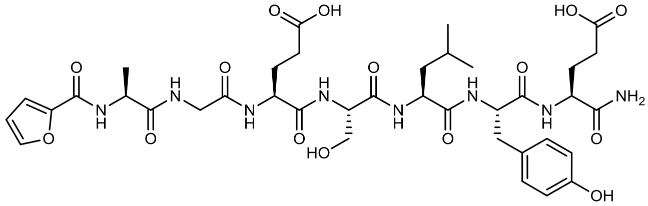

MAI-506

(4S,7S,10S,13S,16S)-4-carbamoyl-16-(2-((S)-2-(furan-2-carboxamido)propanamido)acetamido)-7-(4-hydroxybenzyl)-13-(hydroxymethyl)-10-isobutyl-6,9,12,15-tetraoxo-5,8,11,14-tetraazanonadecanedioic acid (MAI-506). General procedure A. White powder, 20 mg, 11.6% yield. ^{1}H NMR (500 MHz, DMSO-d_6) δ 8.40 (d, *J* = 7.0 Hz, 1H), 8.29 (t, *J* = 5.8 Hz, 1H), 8.05 (d, *J* = 7.7 Hz, 1H), 7.99 (d, *J* = 7.4 Hz, 1H), 7.91 (d, *J* = 7.8 Hz, 1H), 7.85 – 7.77 (m, 3H), 7.17 (d, *J* = 3.4 Hz, 1H), 7.08 (d, *J* = 4.0 Hz, 2H), 7.02 – 6.97 (m, 2H), 6.65 – 6.61 (m, 3H), 4.44 – 4.28 (m, 4H), 4.21 – 4.11 (m, 2H), 3.72 (d, *J* = 5.8 Hz, 2H), 3.63 – 3.54 (m, 2H), 2.92 (dd, *J* = 14.1, 4.9 Hz, 1H), 2.66 (dd, *J* = 14.1, 4.9 Hz, 1H), 2.25 – 2.22 (m, 4H), 1.95 – 1.86 (m, 2H), 1.79 – 1.72 (m, 2H), 1.57 – 1.51 (m, 1H), 1.38 – 1.32 (m, 4H), 0.82 (d, *J* = 6.6 Hz, 3H), 0.77 (d, *J* = 6.6 Hz, 3H). ^{13}C NMR (126 MHz, DMSO) δ 173.97, 173.92, 172.86, 172.59, 171.94,

171.15, 170.90, 170.17, 168.79, 157.64, 155.76, 147.42, 145.16, 130.02, 127.64, 114.88, 113.94, 111.87, 61.49, 54.84, 54.36, 51.85, 51.77, 51.54, 48.53, 42.14, 40.29, 36.29, 30.09, 29.97, 27.46, 27.25, 24.04, 22.95, 21.40, 17.78. MS *m/z*: 859.4 $[M-H]^-$; HRMS m/z $[M+H]^+$: calcd. for $C_{38}H_{53}O_{15}N_8$, 861.3625; found, 861.3621. HPLC Relative purity: 98.5%.

(3S,6S,9S,12S,15S,18S,21S,24S,27S,30S,33S)-18-(2-amino-2-oxoethyl)-30-(3-amino-3-oxopropyl)-3-((2-(((S)-1-amino-6-(3-(3',6'-dihydroxy-3-oxo-3H-spiro[isobenzofuran-1,9'-xanthen]-5-yl)thioureido)-1-oxohexan-2-yl)amino)-2-oxoethyl)carbamoyl)-9,21-di((S)-sec-butyl)-15-(2-carboxyethyl)-6-(hydroxymethyl)-12,27-diisobutyl-24-methyl-5,8,11,14,17,20,23,26,29,32-decaoxo-33-(4,7,10,13-tetraoxo-3,6,9,12-tetraazatetradecanamido)-4,7,10,13,16,19,22,25,28,31-decaazahexatriacontanedioic acid (tracer 1). General procedure B. Yellow powder, 21 mg, 15.5% yield. MS m/z: calcd. for $C_{92}H_{130}N_{22}O_{32}S$, 1043.5$[M-2H]^{2-}$; HPLC Relative purity: 98.2%.

(3S,6S,9S,12S,15S,18S,21S,24S,27S,30S,33S)-18-(2-amino-2-oxoethyl)-3-((2-((1-amino-6-(3-(3',6'-dihydroxy-3-oxo-3H-spiro[isobenzofuran-1,9'-xanthen]-5-yl)thioureido)-1-oxohexan-2-yl)amino)-2-oxoethyl)carbamoyl)-9,21-di((S)-sec-butyl)-15-(2-carboxyethyl)-6-(hydroxymethyl)-12,27-diisobutyl-24,30-dimethyl-33-((S)-5-methyl-4,7,10,13-tetraoxo-3,6,9,12-tetraaza-tetradecanamido)-5,8,11,14,17,20,23,26,29,32-decaoxo-4,7,10,13,16,19,22,25,28,31-decaazahexatriacontanedioic acid (tracer 2). General procedure B. Yellow powder, 18 mg, 13.6% yield. MS m/z: calcd. for $C_{91}H_{129}N_{21}O_{31}S$, 1024.54$[M+2H]^{2+}$; HPLC Relative purity: 98.2%.

(3S,6S,9S,12S,15S,18S,21S,24S,27S,30S,33S)-18-(2-amino-2-oxoethyl)-3-((2-((1-amino-6-(3-(3',6'-dihydroxy-3-oxo-3H-spiro[isobenzofuran-1,9'-xanthen]-5-yl)thioureido)-1-oxohexan-2-yl)amino)-2-oxoethyl)carbamoyl)-9-((S)-sec-butyl)-15,21-bis(2-carboxyethyl)-24-(4-hydroxybenzyl)-6,30-bis(hydroxymethyl)-12,27-diisobutyl-33-((S)-5-methyl-4,7,10,13-tetraoxo-3,6,9,12-tetraazatetradecanamido)-5,8,11,14,17,20,23,26,29,32-decaoxo-4,7,10,13,16,19,22,25,28,31-decaazahexatriacontane-dioic acid (tracer 3). General procedure B. Yellow powder, 22 mg, 15.7% yield. MS m/z: calcd. for $C_{96}H_{129}N_{21}O_{35}S$, 1087.04$[M+2H]^{2+}$; HPLC Relative purity: 98.5%.

(3S,6S,9S,12S,15S,18S,21S,24S,27S,30S,33S)-18-(2-amino-2-oxoethyl)-3-((2-((1-amino-6-(3-(3',6'-dihydroxy-3-oxo-3H-spiro [isobenzofuran-1,9'-xanthen]-5-yl)thioureido)-1-oxohexan-2-yl)amino)-2-oxoethyl)carbamoyl)-9-((S)-sec-butyl)-15,21-bis(2-carboxyethyl)-24-(4-hydroxybenzyl)-33-((5S,11S)-11-(4-hydroxybenzyl)-5-methyl-4,7,10,13-tetraoxo-3,6,9,12-tetraazatetra-decanamido)-6,30-bis(hydroxymethyl)-12,27-diisobutyl-5,8,11,14,17,20,23,26,29,32-decaoxo-4,7,10,13,16,19,22,25,28,31-decaazahexatriacontanedioic acid (tracer 4). Yellow powder, 25 mg, 17% yield. General procedure B. MS m/z: calcd. for $C_{103}H_{135}N_{21}O_{36}S$, 1139.0 $[M+2H]^{2+}$; HPLC Relative purity: 98.5%.

(3S,6S,9S,12S,15S,18S,21S,24S,27S,30S,33S,39S,45S)-45-acetamido-18-(2-amino-2-oxoethyl)-3-((2-((1-amino-6-(3-(3',6'-dihydroxy-3-oxo-3H-spiro[isobenzofuran-1,9'-xanthen]-5-yl)thioureido)-1-oxohexan-2-yl)amino)-2-oxoethyl)carbamoyl)-9-((S)-sec-butyl)-15,21,33-tris(2-carboxyethyl)-24-(4-hydroxybenzyl)-6,30-bis(hydroxymethyl)-12,27-diisobutyl-39-methyl-5,8,11,14,17,20,23,26,29,32,35,38,41,44-tetradecaoxo-4,7,10,13,16,19,22,25,28,31,34,37,40,43-tetradecaazaoctatetracontane-dioic acid (tracer 5). Yellow powder, 22 mg, 15.2% yield. General procedure B. MS m/z: calcd. for $C_{99}H_{133}N_{21}O_{37}S$, 1119.7 $[M-2H]^{2-}$; HPLC Relative purity: 98.2%.

(3S,6S,9S,12S,15S,18S,21S,24S,27S,30S,33S)-18-(2-amino-2-oxoethyl)-3-((2-((1-amino-6-(3-(3',6'-dihydroxy-3-oxo-3H-spiro[isobenzofuran-1,9'-xanthen]-5-yl)thioureido)-1-oxohexan-2-yl)amino)-2-oxoethyl)carbamoyl)-33-(2-((S)-2-(((benzyloxy) carbonyl)amino)propanamido)acetamido)-9-((S)-sec-butyl)-15,21-bis(2-carboxyethyl)-24-(4-hydroxybenzyl)-6,30-bis(hydroxymethyl)-12,27-diisobutyl-5,8,11,14,17,20,23,26,29,32-decaoxo-4,7,10,13,16,19,22,25,28,31-decaazahexatri-acontanedioic acid (Tracer 6). General procedure B. Yellow powder, 20 mg, 14.4% yield. MS m/z: calcd. for $C_{98}H_{127}N_{19}O_{34}S$, 1072.67, 2 $[M-H]^-$; HPLC Relative purity: 98.0%.

(4S,7S,10S,13S,16S)-4-(((R)-1-amino-6-(3-(3',6'-dihydroxy-3-oxo-3H-spiro[isobenzofuran-1,9'-xanthen]-5-yl)thioureido)-1-oxohexan-2-yl)carbamoyl)-16-(2-((S)-2-(((benzyloxy)carbonyl)amino)propanamido)acetamido)-7-(4-hydroxybenzyl)-13-(hydroxymethyl)-10-isobutyl-6,9,12,15-tetraoxo-5,8,11,14-tetraazanonadecanedioic acid (tracer 7). General procedure B. Yellow powder, 15 mg, 16.3% yield. MS m/z: calcd. for $C_{68}H_{79}N_{11}O_{21}S$, 708.35 $[M-2H]^{2-}$; HPLC Relative purity: 98.2%.

(4S,7S,10S,13S,16S,19S,22S)-7-(2-amino-2-oxoethyl)-22-(2-((S)-2-(((benzyloxy)carbonyl)amino)propanamido)acetamido)-4-(((7S,10S,13S,16S)-13-((S)-sec-butyl)-7-carbamoyl-1-((3',6'-dihydroxy-3-oxo-3H-spiro[isobenzofuran-1,9'-xanthen]-5-

yl)amino)-10-(hydroxymethyl)-18-methyl-9,12,15-trioxo-1-thioxo-2,8,11,14-tetraazanonadecan-16-yl)carbamoyl)-10-(2-carboxyethyl)-13-(4-hydroxybenzyl)-19-(hydroxymethyl)-16-isobutyl-6,9,12,15,18,21-hexaoxo-5,8,11,14,17,20-hexaazapenta-cosanedioic acid (tracer 8). General procedure B. Yellow powder, 20 mg, 15.6% yield. MS m/z: calcd. for $C_{91}H_{129}N_{21}O_{31}S$, 986.62 $[M-2H]^{2-}$; HPLC Relative purity: 98.0%.

(4S,7S,10S,13S,16S,19S,22S)-7-(2-amino-2-oxoethyl)-4-(((S)-1-(((S)-1-amino-6-(3-(3',6'-dihydroxy-3-oxo-3H-spiro[isobenzo-furan-1,9'-xanthen]-5-yl)thioureido)-1-oxohexan-2-yl)amino)-4-methyl-1-oxopentan-2-yl)carbamoyl)-22-(2-((S)-2-(((benzyloxy)carbonyl)amino)propanamido)acetamido)-10-(2-carboxyethyl)-13-(4-hydroxybenzyl)-19-(hydroxymethyl)-16-isobutyl-6,9,12,15,18,21-hexaoxo-5,8,11,14,17,20-hexaazapentacosanedioic acid (tracer 9). General procedure B. Yellow powder, 19 mg, 16.5% yield. MS m/z: calcd. for $C_{83}H_{103}N_{15}O_{27}S$, 1774.87 $[M+H]^{+}$; HPLC Relative purity: 98.1%.

(4S,7S,10S,13S,16S)-4-(((S)-4-amino-1-(((S)-1-amino-6-(3-(3',6'-dihydroxy-3-oxo-3H-spiro[isobenzofuran-1,9'-xanthen]-5-yl)thioureido)-1-oxohexan-2-yl)amino)-1,4-dioxobutan-2-yl)carbamoyl)-16-(2-((S)-2-(((benzyloxy)carbonyl)amino) propanamido)acetamido)-7-(4-hydroxybenzyl)-13-(hydroxymethyl)-10-isobutyl-6,9,12,15-tetraoxo-5,8,11,14-tetraazanona-decanedioic acid (tracer 10). General procedure B. Yellow powder, 20 mg, 20% yield. MS m/z: calcd. for $C_{72}H_{85}N_{13}O_{23}S$, 1531.38 $[M-H]^{-}$; HPLC Relative purity: 98.0%.

(4S,7S,10S,13S,16S)-16-(2-((S)-2-(((benzyloxy)carbonyl)amino)propanamido)acetamido)-4-((7-carbamoyl-1-((3',6'-dihydroxy-3-oxo-3H-spiro[isobenzofuran-1,9'-xanthen]-5-yl)amino)-9,12,15,18,21,24,27-heptaoxo-1-thioxo-2,8,11,14,17,20,23,26-octaazaoctacosan-28-yl)carbamoyl)-7-(4-hydroxybenzyl)-13-(hydroxymethyl)-10-isobutyl-6,9,12,15-tetraoxo-5,8,11,14-tetraazanonadecanedioic acid (tracer 11). General procedure B. Yellow powder, 20 mg, 17% yield. MS m/z: calcd. for $C_{82}H_{100}N_{18}O_{28}S$, 1817.55 $[M+H]^{+}$; HPLC Relative purity: 98.1%. HPLC purity yield: 20 mg, 12%.

(4S,7S,10S,13S,16S)-16-(2-((S)-2-(((benzyloxy)carbonyl)amino)propanamido)acetamido)-4-((7-carbamoyl-1-((3',6'-dihydroxy-3-oxo-3H-spiro[isobenzofuran-1,9'-xanthen]-5-yl)amino)-9,12,15,18,21-pentaoxo-1-thioxo-2,8,11,14,17,20-hexaazadocosan-22-yl)carbamoyl)-7-(4-hydroxybenzyl)-13-(hydroxymethyl)-10-isobutyl-6,9,12,15-tetraoxo-5,8,11,14-tetraazanonadecanedioic acid (tracer 12). Yellow powder, 19 mg, 17.3% yield. General procedure B. MS m/z: calcd. for $C_{78}H_{94}N_{16}O_{26}S$, 1703.61 $[M+H]^{+}$; HPLC Relative purity: 98.0%.

(4S,7S,10S,13S,16S)-16-(2-((S)-2-(((benzyloxy)carbonyl)amino)propanamido)acetamido)-4-((7-carbamoyl-1-((3',6'-dihydroxy-3-oxo-3H-spiro[isobenzofuran-1,9'-xanthen]-5-yl)amino)-9,12,15-trioxo-1-thioxo-2,8,11,14-tetraazahexadecan-16-yl) carbamoyl)-7-(4-hydroxybenzyl)-13-(hydroxymethyl)-10-isobutyl-6,9,12,15-tetraoxo-5,8,11,14-tetraazanonadecanedioic acid (tracer 13). Yellow powder, 18 mg, 17.5% yield. General procedure B. MS m/z: calcd. for $C_{74}H_{88}N_{14}O_{24}S$, 1589.57 $[M+H]^{+}$; HPLC Relative purity: 99.4%.

(4S,7S,10S,13S,16S)-4-((2-((1-amino-6-(3-(3',6'-dihydroxy-3-oxo-3H-spiro[isobenzofuran-1,9'-xanthen]-5-yl)thioureido)-1-oxohexan-2-yl)amino)-2-oxoethyl)carbamoyl)-16-(2-((S)-2-(((benzyloxy)carbonyl)amino)propanamido)acetamido)-7-(4-hydroxybenzyl)-13-(hydroxymethyl)-10-isobutyl-6,9,12,15-tetraoxo-5,8,11,14-tetraazanonadecanedioic acid (tracer 14). Yellow powder, 15 mg, 15.7% yield. General procedure B. MS m/z: calcd. for $C_{70}H_{82}N_{12}O_{22}S$, 1475.27 $[M-H]^{-}$; HPLC Relative purity: 98.1%.

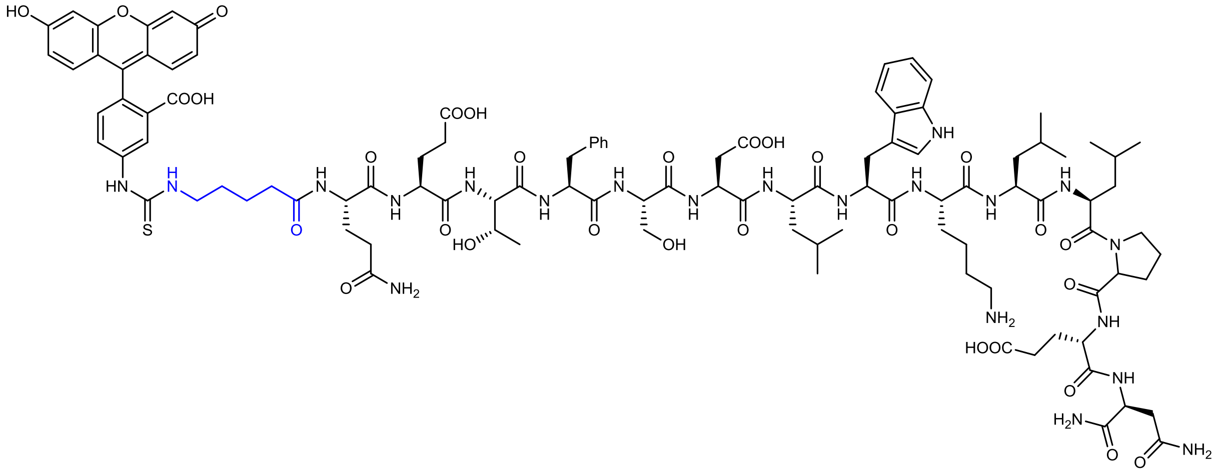

(3S,6S,9S,12S,15S)-3-(((4S,7S,10S,13S,16S)-13-((1H-indol-3-yl)methyl)-10-(4-aminobutyl)-4-(2-(((S)-4-carboxy-1-(((S)-1,4-diamino-1,4-dioxobutan-2-yl)amino)-1-oxobutan-2-yl)carbamoyl)pyrrolidine-1-carbonyl)-7-isobutyl-2,18-dimethyl-6,9,12,15-tetraoxo-5,8,11,14-tetraazanonadecan-16-yl)carbamoyl)-15-((S)-5-amino-2-(5-(3-(3-carboxy-4-(6-hydroxy-3-oxo-3H-xanthen-9-yl)phenyl)thioureido)pentanamido)-5-oxopentanamido)-9-benzyl-12-((S)-1-hydroxyethyl)-6-(hydroxymethyl)-5,8,11,14-tetraoxo-4,7,10,13-tetraazaoctadecanedioic acid (PS-P53-7G). General procedure B. Yellow powder, 41 mg, 28.7% yield. HRMS (ESI) ([M+H]$^+$) Calcd. for $C_{105}H_{139}N_{21}O_{30}S$: 2205.9717, found: 736.6657[M+3H]$^{3+}$, 1104.4933[M+2H]$^{2+}$. HPLC purity: 99.5 %; Retention time: 20.181 min.

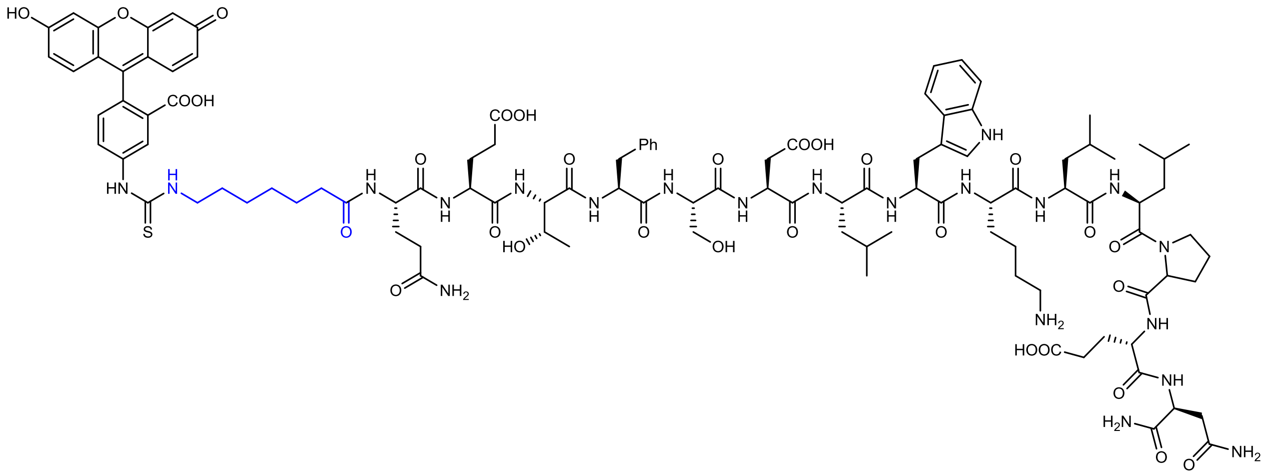

(3S,6S,9S,12S,15S)-3-(((4S,7S,10S,13S,16S)-13-((1H-indol-3-yl)methyl)-10-(4-aminobutyl)-4-(2-(((S)-4-carboxy-1-(((S)-1,4-diamino-1,4-dioxobutan-2-yl)amino)-1-oxobutan-2-yl)carbamoyl)pyrrolidine-1-carbonyl)-7-isobutyl-2,18-dimethyl-6,9,12,15-tetraoxo-5,8,11,14-tetraazanonadecan-16-yl)carbamoyl)-15-((S)-5-amino-2-(7-(3-(3-carboxy-4-(6-hydroxy-3-oxo-3H-xanthen-9-yl)phenyl)thioureido)heptanamido)-5-oxopentanamido)-9-benzyl-12-((S)-1-hydroxyethyl)-6-(hydroxymethyl)-5,8,11,14-tetraoxo-4,7,10,13-tetraazaoctadecanedioic acid (PS-P53-8H). General procedure B. Yellow powder, 39 mg, 27.1% yield. HRMS (ESI) ([M+H]$^+$) Calcd. for $C_{107}H_{143}N_{21}O_{30}S$: 2234.0030, found: 559.5114 [M+4H]$^{4+}$, 746.0092[M+3H]$^{3+}$, 1118.5086[M+2H]$^{2+}$. HPLC purity: 99.7 %; Retention time: 21.270 min.

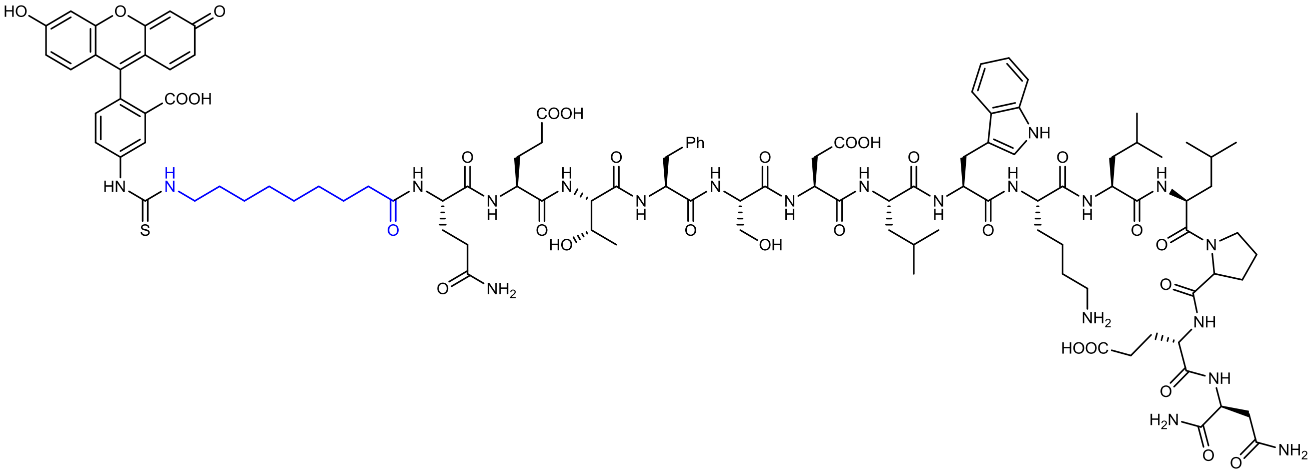

(3S,6S,9S,12S,15S)-3-(((4S,7S,10S,13S,16S)-13-((1H-indol-3-yl)methyl)-10-(4-aminobutyl)-4-(2-(((S)-4-carboxy-1-(((S)-1,4-diamino-1,4-dioxobutan-2-yl)amino)-1-oxobutan-2-yl)carbamoyl)pyrrolidine-1-carbonyl)-7-isobutyl-2,18-dimethyl-6,9,12,15-tetraoxo-5,8,11,14-tetraazanonadecan-16-yl)carbamoyl)-15-((S)-5-amino-2-(9-(3-(3-carboxy-4-(6-hydroxy-3-oxo-3H-xanthen-9-yl)phenyl)thioureido)nonanamido)-5-oxopentanamido)-9-benzyl-12-((S)-1-hydroxyethyl)-6-(hydroxymethyl)-5,8,11,14-tetraoxo-4,7,10,13-tetraazaoctadecanedioic acid (PS-P53-9I). General procedure B. Yellow powder, 36 mg, 24.7% yield. HRMS (ESI) ($[M+H]^+$) Calcd. for $C_{109}H_{147}N_{21}O_{30}S$: 2262.0343, found: 566.5154 $[M+4H]^{4+}$, 755.3528$[M+3H]^{3+}$, 1132.5240$[M+2H]^{2+}$. HPLC purity: 99.5 %; Retention time: 21.807 min.

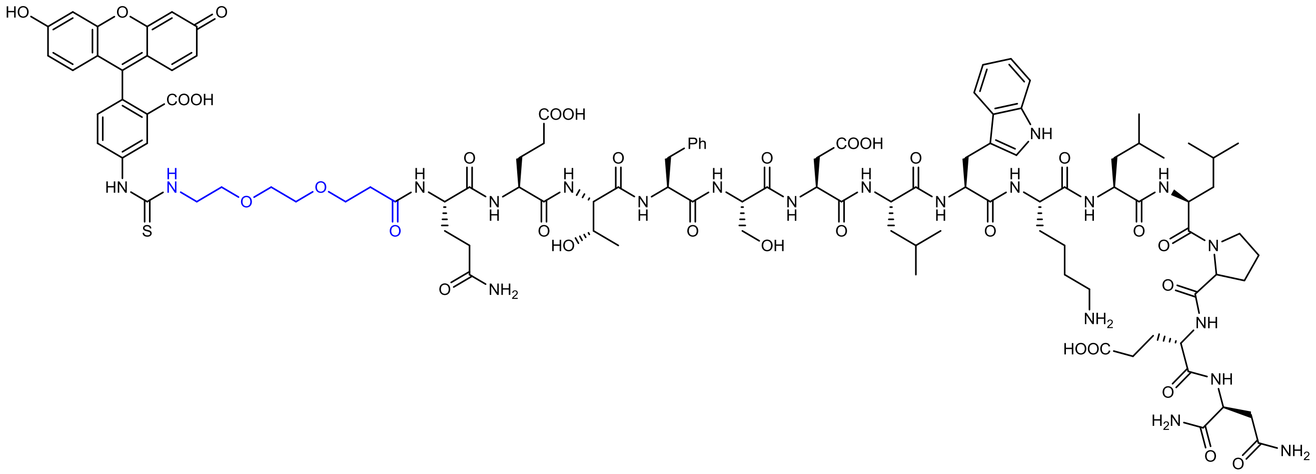

(3S,6S,9S,12S,15S)-3-(((4S,7S,10S,13S,16S)-13-((1H-indol-3-yl)methyl)-10-(4-aminobutyl)-4-(2-(((S)-4-carboxy-1-(((S)-1,4-diamino-1,4-dioxobutan-2-yl)amino)-1-oxobutan-2-yl)carbamoyl)pyrrolidine-1-carbonyl)-7-isobutyl-2,18-dimethyl-6,9,12,15-tetraoxo-5,8,11,14-tetraazanonadecan-16-yl)carbamoyl)-15-((S)-13-(3-amino-3-oxopropyl)-1-((3-carboxy-4-(6-hydroxy-3-oxo-3H-xanthen-9-yl)phenyl)amino)-11-oxo-1-thioxo-5,8-dioxa-2,12-diazatetradecan-14-amido)-9-benzyl-12-((S)-1-hydroxyethyl)-6-(hydroxymethyl)-5,8,11,14-tetraoxo-4,7,10,13-tetraazaoctadecanedioic acid (PS-P53-10J). General procedure B. Yellow powder, 32 mg, 21.9% yield. HRMS (ESI) ($[M+H]^+$) Calcd. for $C_{107}H_{143}N_{21}O_{32}S$: 2265.9929, found: 756.6712$[M+3H]^{3+}$, 1134.5021$[M+2H]^{2+}$. HPLC purity: 99.6 %; Retention time: 20.602 min.

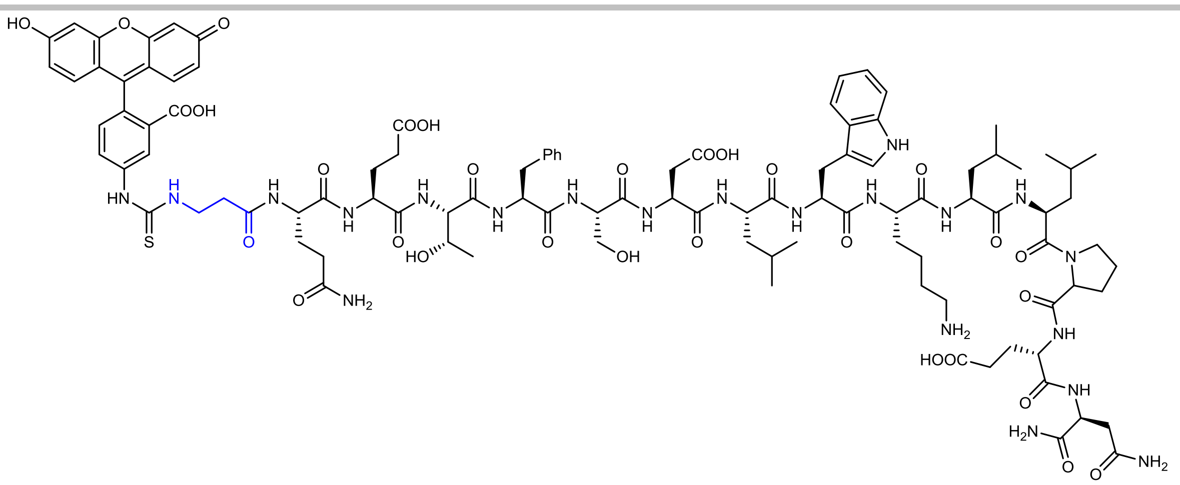

(3S,6S,9S,12S,15S)-3-(((4S,7S,10S,13S,16S)-13-((1H-indol-3-yl)methyl)-10-(4-aminobutyl)-4-(2-(((S)-4-carboxy-1-(((S)-1,4-diamino-1,4-dioxobutan-2-yl)amino)-1-oxobutan-2-yl)carbamoyl)pyrrolidine-1-carbonyl)-7-isobutyl-2,18-dimethyl-6,9,12,15-tetraoxo-5,8,11,14-tetraazanonadecan-16-yl)carbamoyl)-15-((S)-5-amino-2-(3-(3-(3-carboxy-4-(6-hydroxy-3-oxo-3H-xanthen-9-yl)phenyl)thioureido)propanamido)-5-oxopentanamido)-9-benzyl-12-((S)-1-hydroxyethyl)-6-(hydroxymethyl)-5,8,11,14-tetraoxo-4,7,10,13-tetraazaoctadecanedioic acid (PS-P53-11K). General procedure B. Yellow powder, 38 mg, 27.0% yield. HRMS (ESI) ([M+H]$^+$) Calcd. for $C_{103}H_{135}N_{21}O_{30}S$: 2177.9404, found: 545.4022 [M+4H]$^{4+}$, 727.3192[M+3H]$^{3+}$, 1090.4747[M+2H]$^{2+}$. HPLC purity: 100.0 %; Retention time: 20.050 min.

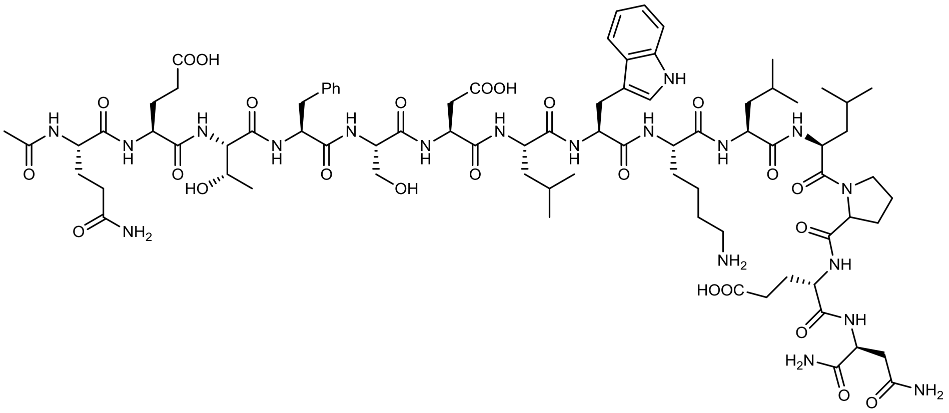

(3S,6S,9S,12S,15S)-3-(((4S,7S,10S,13S,16S)-13-((1H-indol-3-yl)methyl)-10-(4-aminobutyl)-4-(2-(((S)-4-carboxy-1-(((S)-1,4-diamino-1,4-dioxobutan-2-yl)amino)-1-oxobutan-2-yl)carbamoyl)pyrrolidine-1-carbonyl)-7-isobutyl-2,18-dimethyl-6,9,12,15-tetraoxo-5,8,11,14-tetraazanonadecan-16-yl)carbamoyl)-15-((S)-2-acetamido-5-amino-5-oxopentanamido)-9-benzyl-12-((S)-1-hydroxyethyl)-6-(hydroxymethyl)-5,8,11,14-tetraoxo-4,7,10,13-tetraazaoctadecanedioic acid (P53). General procedure B. White powder, 35 mg, 30.7% yield. HRMS (ESI) ([M+H]$^+$) Calcd. for $C_{81}H_{121}N_{19}O_{25}$: 1759.8781, found: 587.6354[M+3H]$^{3+}$, 881.4484[M+2H]$^{2+}$. HPLC purity: 100.0 %; Retention time: 18.503 min.

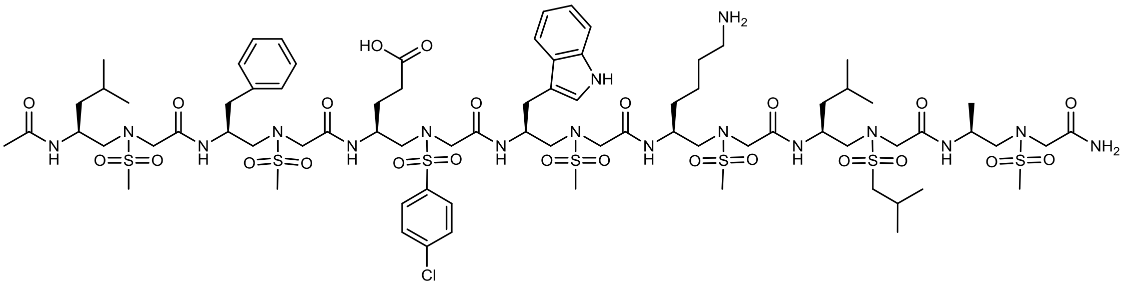

(5S,11S,17S,23S,29S)-23-((1H-indol-3-yl)methyl)-1-amino-17-(4-aminobutyl)-29-((5S,11S)-5-benzyl-11-isobutyl-3,9-bis(methyls -ulfonyl)-7,13-dioxo-3,6,9,12-tetraazatetradecanamido)-27-((4-chlorophenyl)sulfonyl)-11-isobutyl-9-(isobutylsulfonyl)-5-meth-

yl-3,15,21-tris(methylsulfonyl)-1,7,13,19,25-pentaoxo-3,6,9,12,15,18,21,24,27-nonaazadotriacontan-32-oic acid (PS5). General procedure B. White powder, 29 mg, 23.2% yield. HRMS (ESI) ($[M+H]^+$) Calcd. for $C_{77}H_{126}ClN_{17}O_{24}S_7$: 1931.6895, found: 645.5686$[M+3H]^{3+}$, 967.8493$[M+2H]^{2+}$. HPLC purity: 99.0 %; Retention time: 24.385 min.

2. Molecular modeling and molecular dynamics

The particular binding structures of tracers 1, 4, and 5 in Supplementary Fig. 1 are generated by molecular modeling and molecular dynamics (MD) simulations as follows: (1) constructed three initial tracer–APC complexes by mutating residue and adding FITC motif into the “GGGGEQLAINELISDGS” segment from Asef based on the crystal structure (PDB ID: 3NMZ) resolved in our previous work [1], followed by energy minimization of the systems; (2) created the topology file and coordinate file of the three systems for the simulations, then built the water box with a cube with sides of 10 Å for each system and added counter ions (Na^+ or Cl^-) to keep the systems in electric neutrality; (3) performed the MD simulations based on the workflow in our previous work[2]. (4) analyzed the trajectories from each simulation by Pymol and selected a representative conformation of the best energy cluster as the particular structure shown in Supplementary Fig. 1.

In the later research, we also performed an MD simulation for the MDM2-P53 system. The initial structure of the simulation system is the structure published by previous work of others, with the PDB ID of 1YCR[3]. The preparation of FITC-modified P53 is similar to the previous flow of tracer 1-FITC, we firstly mutated the Asparagine of C-Terminal to Lysine, and added the FITC moiety to the Lysine by the Discovery Studio software. After adding blocking terminals of modified P53, we carried the energy minimization for the P53-FITC – MDM2 complex by the software and generated the forcefield for the P53-FITC using Amber. Then we performed the MD simulation followed the preceding workflow of simulation.

3. Fluorescence Polarization Competition Assays of p53-MDM2

The binding affinity (K_d) of peptides was obtained by fluorescence polarization (FP). GST-MDM2-3-150 containing human MDM2 were expressed in *E. coli* as previously described by us. The fluorescence polarization assay was conducted by incubating MDM2 (0 to 1 μM) with 25 nM FITC-labeled peptide in 1× PBS with 0.1% Pluronic F-68. Dissociation constants (K_d) were determined by plotting the fluorescence anisotropy values as a function of protein concentration, and the plots were fitted to the following equation. The L_{st} is the concentration of the peptides, and the x stands for the concentration of the protein. The experiments were performed in triplicates and repeated for three times. (Supplementary Equation (1))

The IC_{50} of peptides was obtained by incubating sulfono-γ-AA peptides PS5 and P53 from 0.01 μM to 60 μM with 25 nM competitor PS-P53-11K or PS-P53-9I and 250 nM GST-MDM2-3-150. The experiments were performed in triplicates and repeated for three times. (Supplementary Equation (2))

Supplementary Equation (1)

$$Y = \mathrm{FP_{min}} + (\mathrm{FP_{max}} - \mathrm{FP_{min}})\frac{(K_d + L_{st} + x) - \sqrt{(K_d + L_{st} + x)^2 - 4L_{st}x}}{2L_{st}}$$

Supplementary Equation (2)

$$Y = \mathrm{FP_{min}} + \frac{\mathrm{FP_{max}} - \mathrm{FP_{min}}}{1 + 10^{x-\log \mathrm{IC_{50}}}}$$

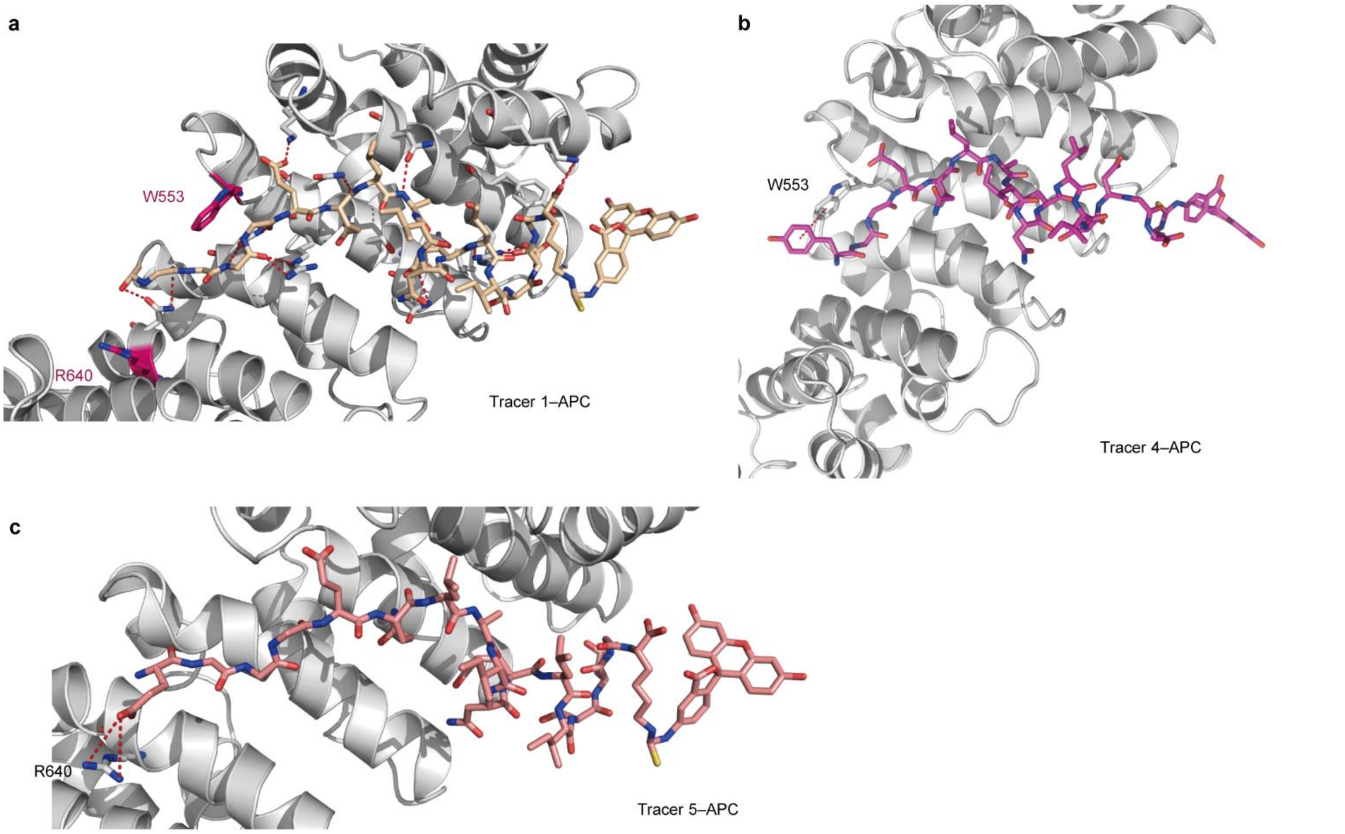

Supplementary Fig. 1. Interaction analysis of tracer1, tracer 4 and tracer 5 complexed with APC. The binding modes of tracer1 (a), tracer 4 (b) and tracer 5 (c) are predicted by molecular dynamics simulations. The initial and final coordinates of the MD simulations are provided in Source Data files.

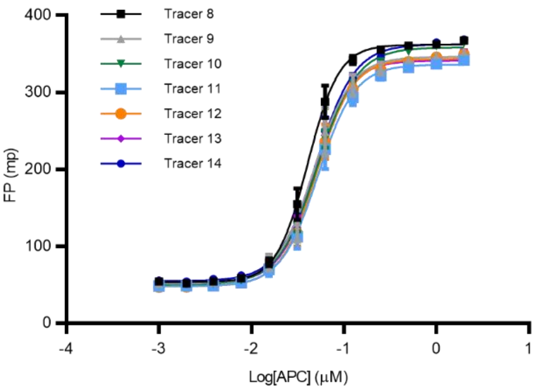

Supplementary Fig. 2. Binding curves for tracers to APC (303-739) determined by FP assay. Data are presented as the mean ± SD (n = 2 independent experiments). The experiments were performed in triplicates and repeated twice. Source data are provided as a Source Data file.

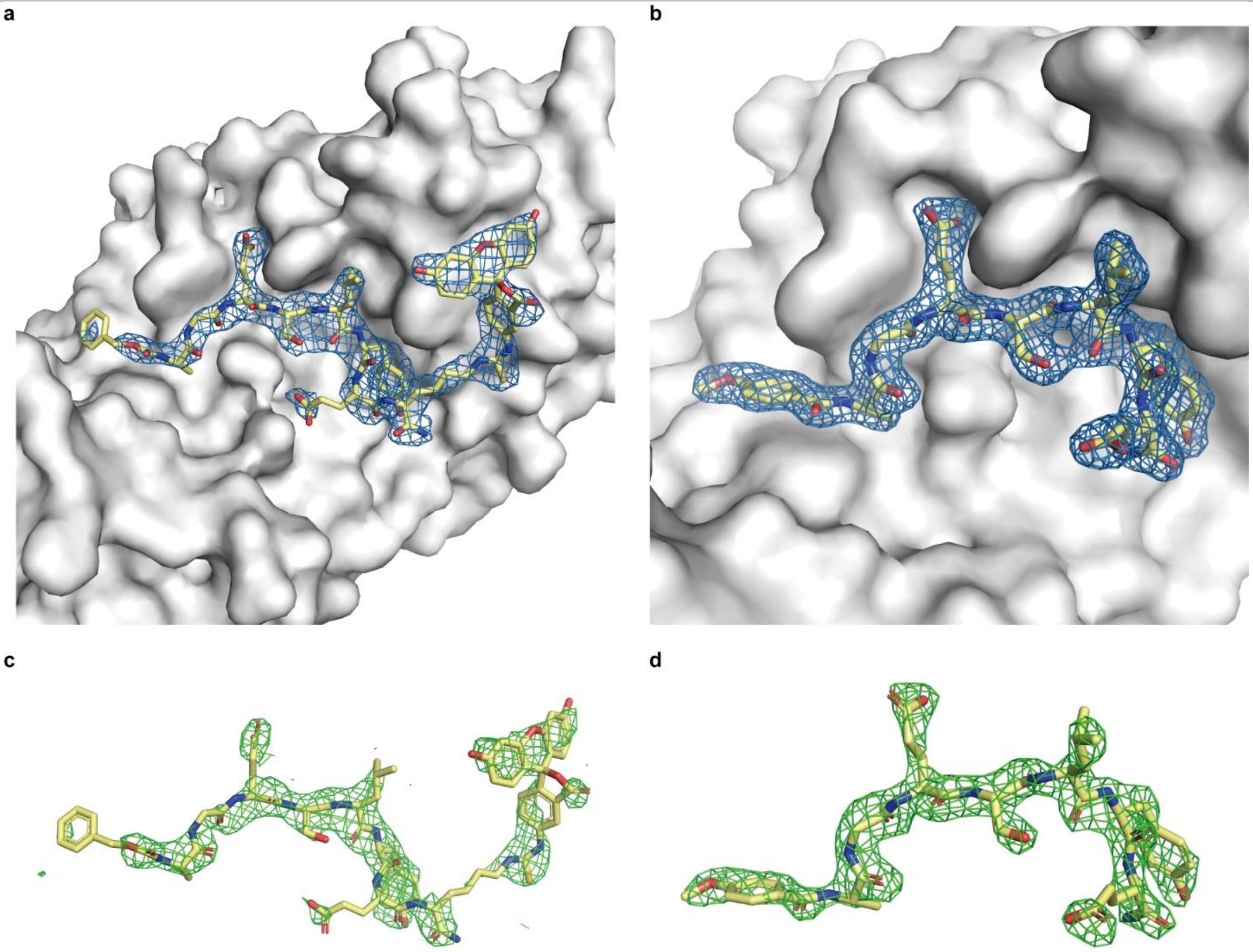

Supplementary Fig. 3. Electron density maps of tracer 7 and MAI-516. a, b) 2Fo-Fc electron density maps for tracer 7 (a) and MAI-516 (b) contoured at 1.0 σ. c, d) Fo-Fc omit maps (green) of tracer 7 (c) and MAI-516 (d) contoured at 3.0 σ.

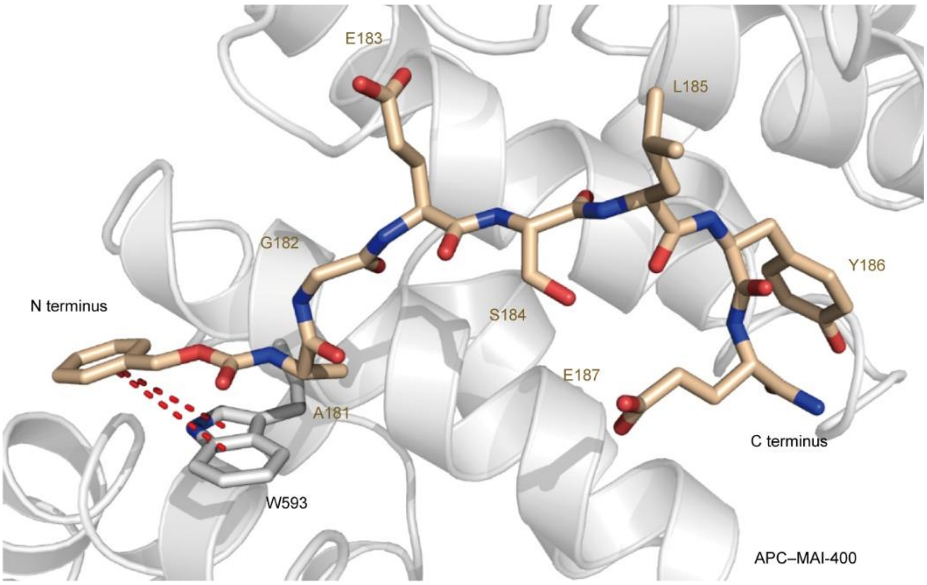

Supplementary Fig. 4. Binding modes of MAI-400 complexed with APC.

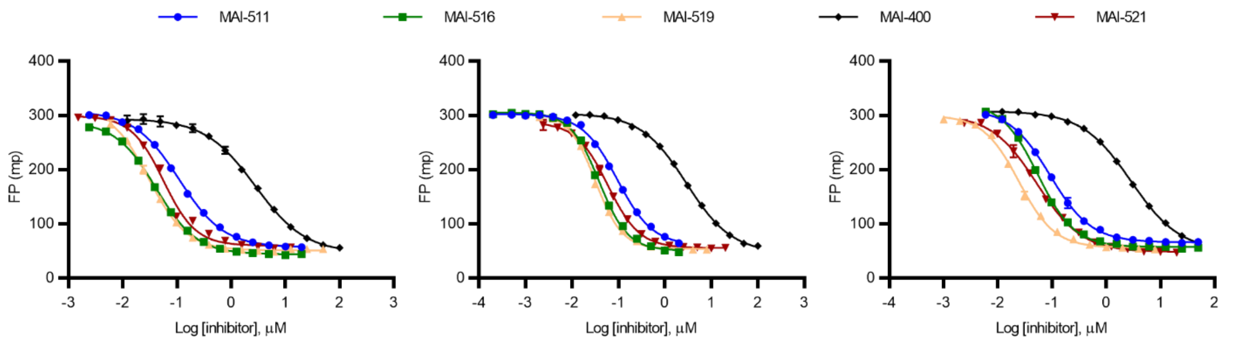

Supplementary Fig. 5. Original plot of FP against concentration for Fig. 3b from three individual experiments.

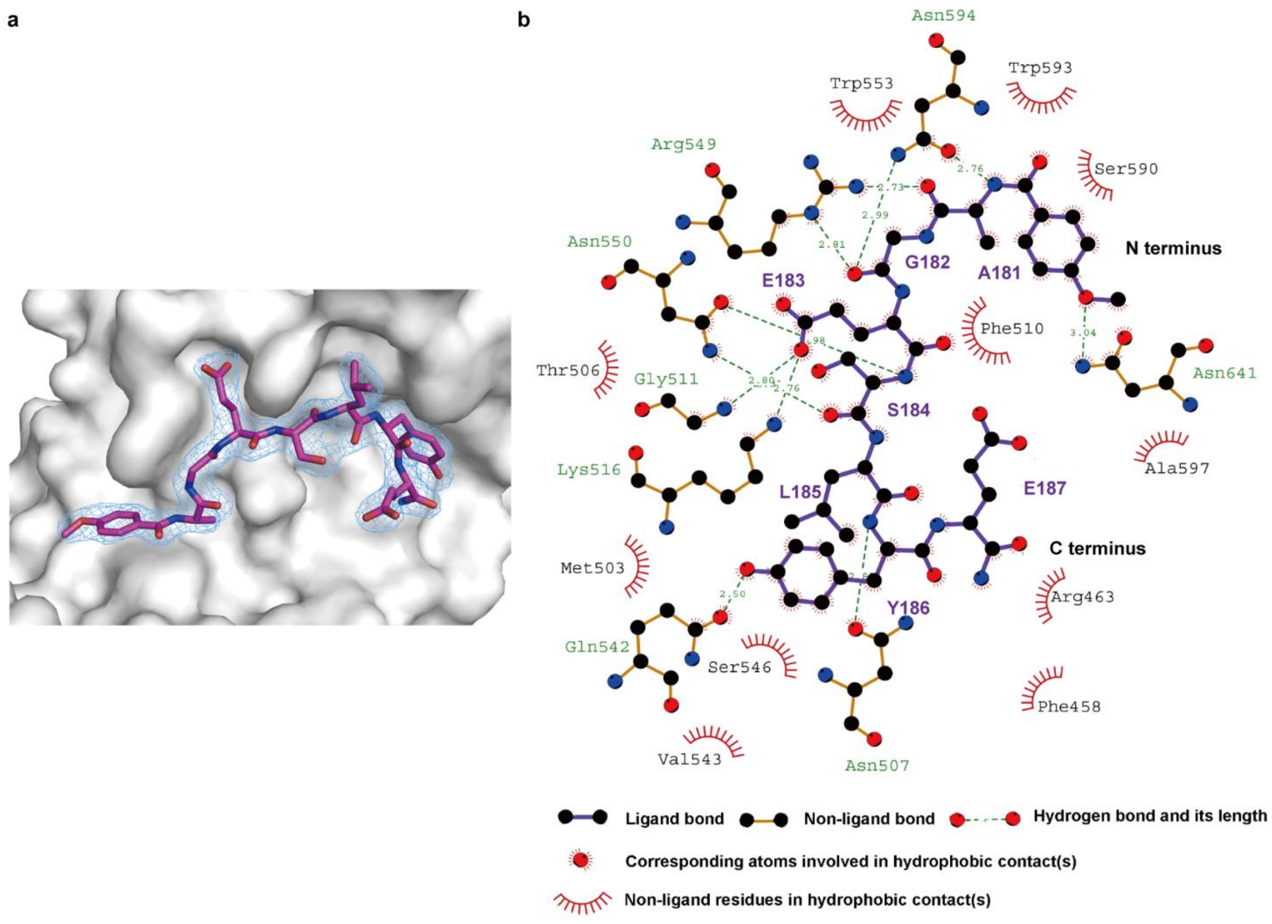

Supplementary Fig. 6. Binding modes of MAI-516 complexed with APC. a) 2Fo-Fc electron density maps for MAI-516 (blue) contoured at 1.0 σ. b) Extensive residue-residue interactions on the interface of the APC–MAI-516 complex structure. The residues belonging to APC and MAI-516 are labeled in yellow and purple, respectively. A distance between donor and acceptor of less than 3.4 Å indicates a hydrogen bond, and a 4.1 Å distance between two hydrophobic atoms indicates a hydrophobic interaction.

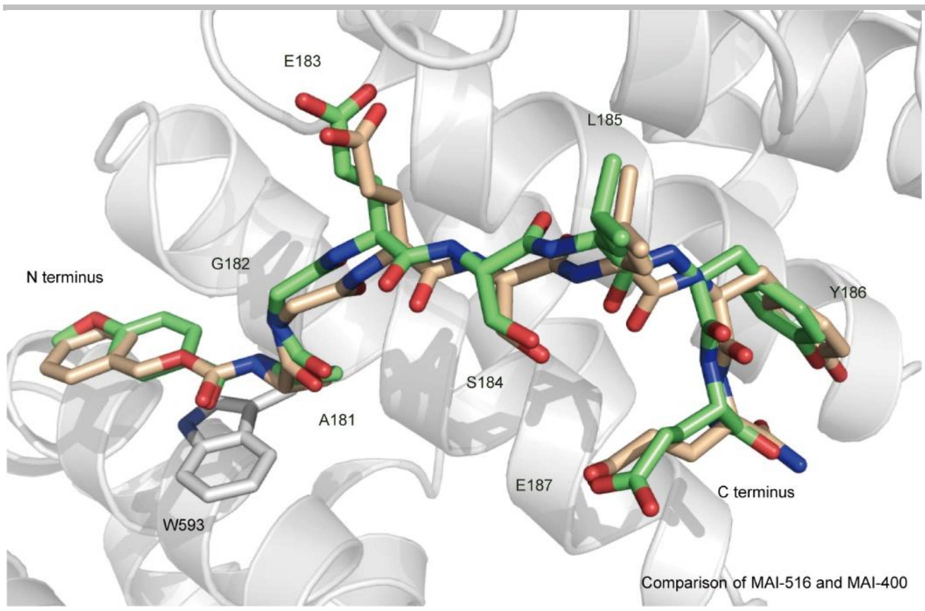

Supplementary Fig. 7. Overlay cocrystal structure of MAI-516 complexed with APC and MAI-400 complexed with APC (PDB code 5Z8H).

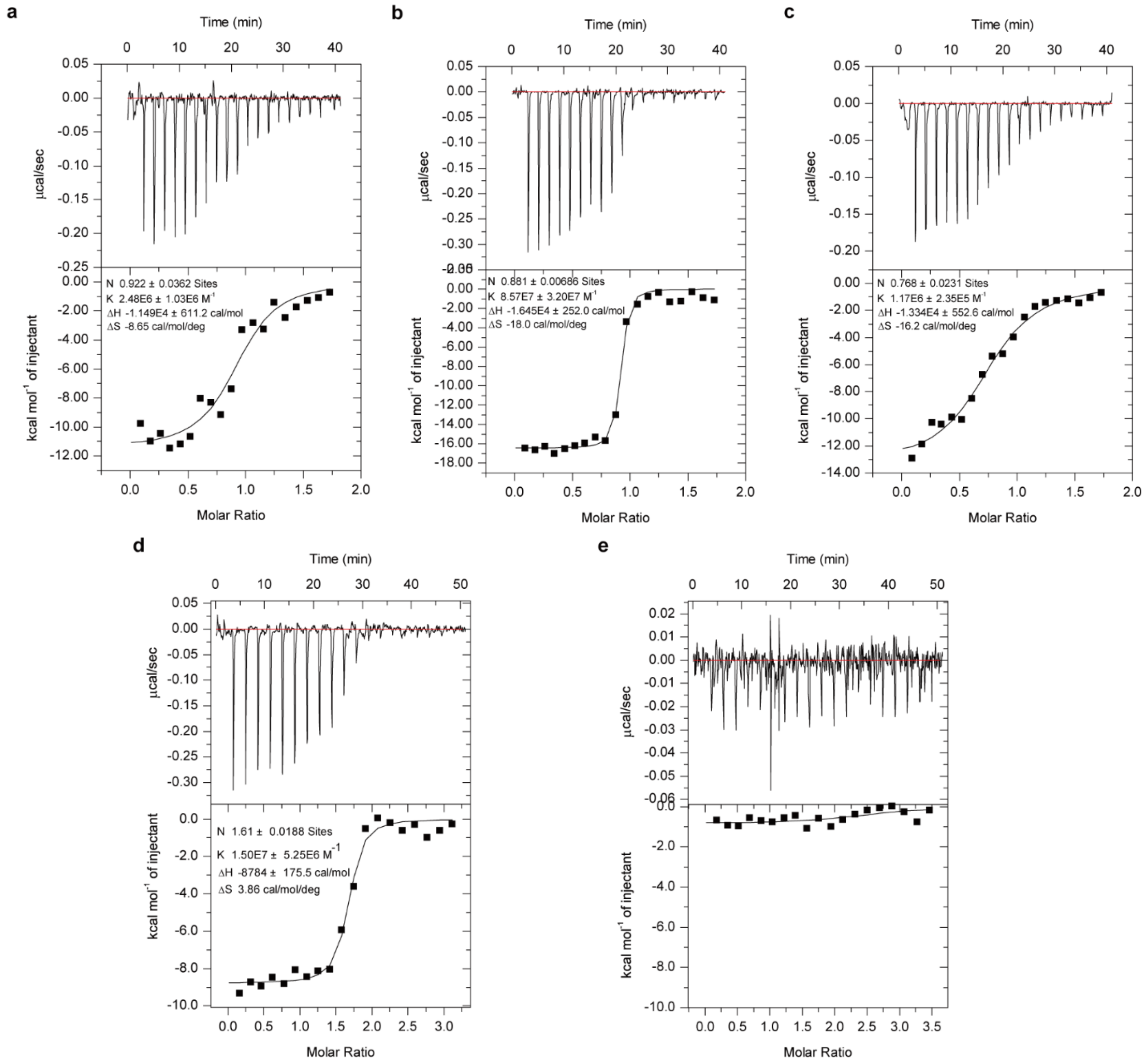

Supplementary Fig. 8. Representative isothermal titration calorimetry (ITC) data. a-c) Representative ITC data for the binding of MAI-516 to the W593A (a), N641A (b) and W593A, N641A (c) mutant of APC (303-739). d) Representative ITC data for the binding of MAIT-516 to APC (303-739). e) Representative ITC data for the binding of TAT+linker to APC (303-739). All experiments were repeated three times with similar results.

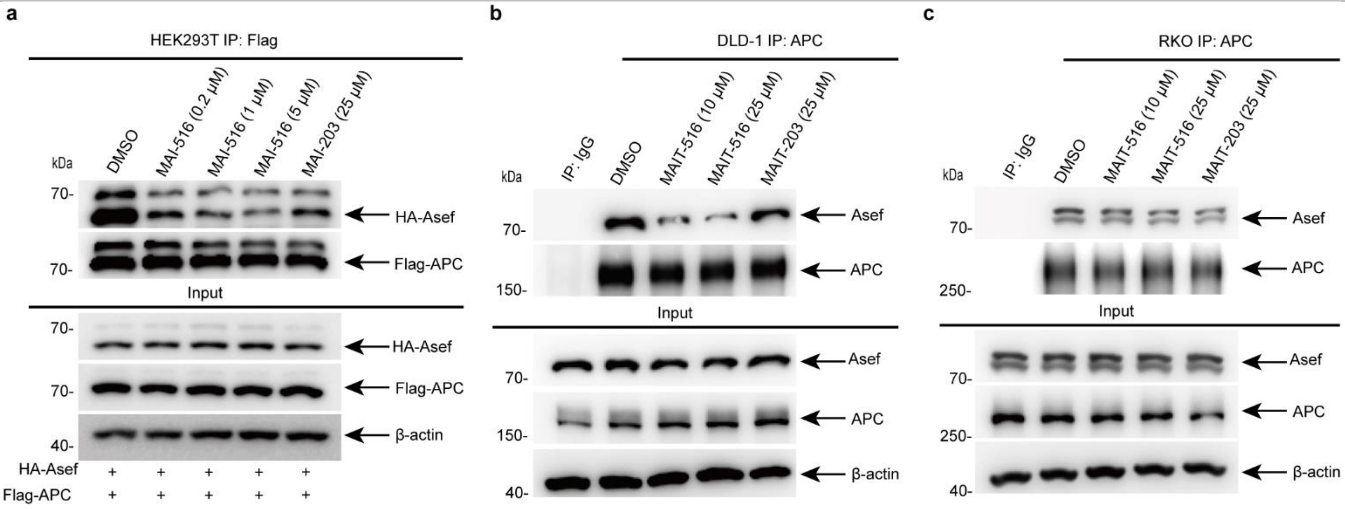

Supplementary Fig. 9. Effect of MAI-516 and MAIT-516 on APC-Asef interaction. a) HEK293T cells were transfected with Flag−APC (303−876) and HA−Asef (170−632). The cell lysate was treated with DMSO, MAI-516 (0.2 μM, 1 μM and 5 μM), and MAI-203 (25 μM) for 2 h. Western blots from a single experiment were developed with anti-HA or anti-Flag antibody. b, c) Western blot analysis for co-IP with anti-APC antibody in DLD-1 (b) and RKO (c) cells treated with 10 μM or 25 μM MAIT-516. β-actin was used as a loading control. All experiments were repeated three times with similar results (n = 3 independent experiments). Full blots are provided in Source Data files.

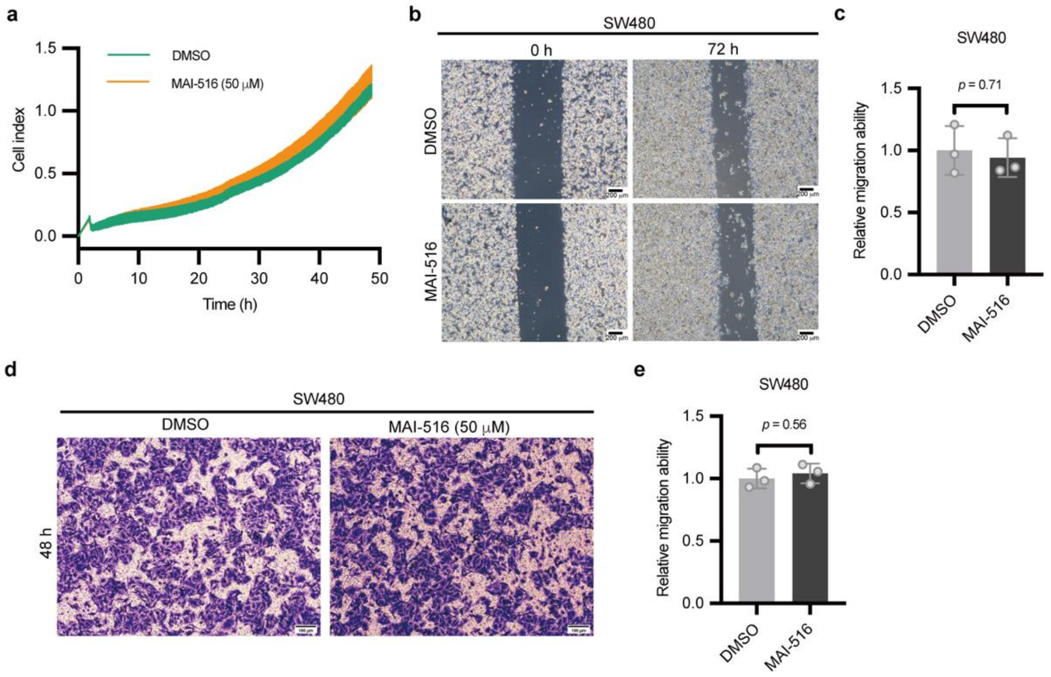

Supplementary Fig. 10. MAI-516 dose not inhibit the migration of SW480 cells. a) Kinetic curves of the migration of SW480 cells after treatment with DMSO or MAI-516 as assessed by xCELLigence RTCA-DP. Data are shown as the mean ± SD. The experiments were performed in 4 replicates and repeated three times (n = 4). b, c) Representative images (b) from wound healing assay of cell cultures treated with MAI-516 (50 μM) for indicated time. Quantitative data of cell migration are expressed relative to the migration ability of DMSO-treated group (c). Scale bar = 200 μm (n = 3 independent experiments). d, e) Representative images (d) of migrated cells stained with crystal violet are displayed. Cells were placed in the upper chamber of transwell and treated with MAI-516 (50 μM) for 48 h. Quantitative data of cell migration are expressed relative to the migration ability of DMSO-treated group (e). Data are represented as the mean ± SD (n = 3 independent experiments). *p* values were calculated by two-tailed unpaired Student's t test. Scale bar = 100 μm. Source data are provided as a Source Data file.

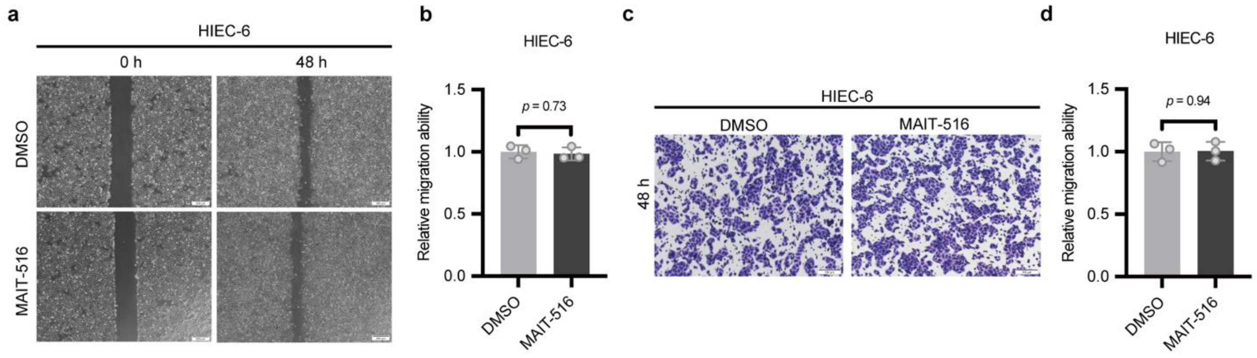

Supplementary Fig. 11. Effect of MAIT-516 on cell migration of normal intestinal epithelial cell HIEC-6 evaluated by wound healing and transwell migration assay. a, b) Representative images (a) from wound healing assay of cell cultures treated with MAIT-516 (25 μM) for indicated time. Quantitative data of cell migration are expressed as relative to the migration ability of DMSO-treated group (b). Scale bar = 200 μm (n = 3 independent experiments). c, d) Representative images (c) of migrated cells stained with crystal violet are displayed. Cells were placed in the upper chamber of transwell inserts and treated with MAIT-516 (25 μM) for 48 h. Quantitative data of cell migration are expressed as relative to the migration ability of DMSO-treated group (d). Data are represented as the mean ± SD (n = 3 independent experiments). Scale bar = 100 μm. *p* values were calculated by two-tailed unpaired Student's t test. Source data are provided as a Source Data file.

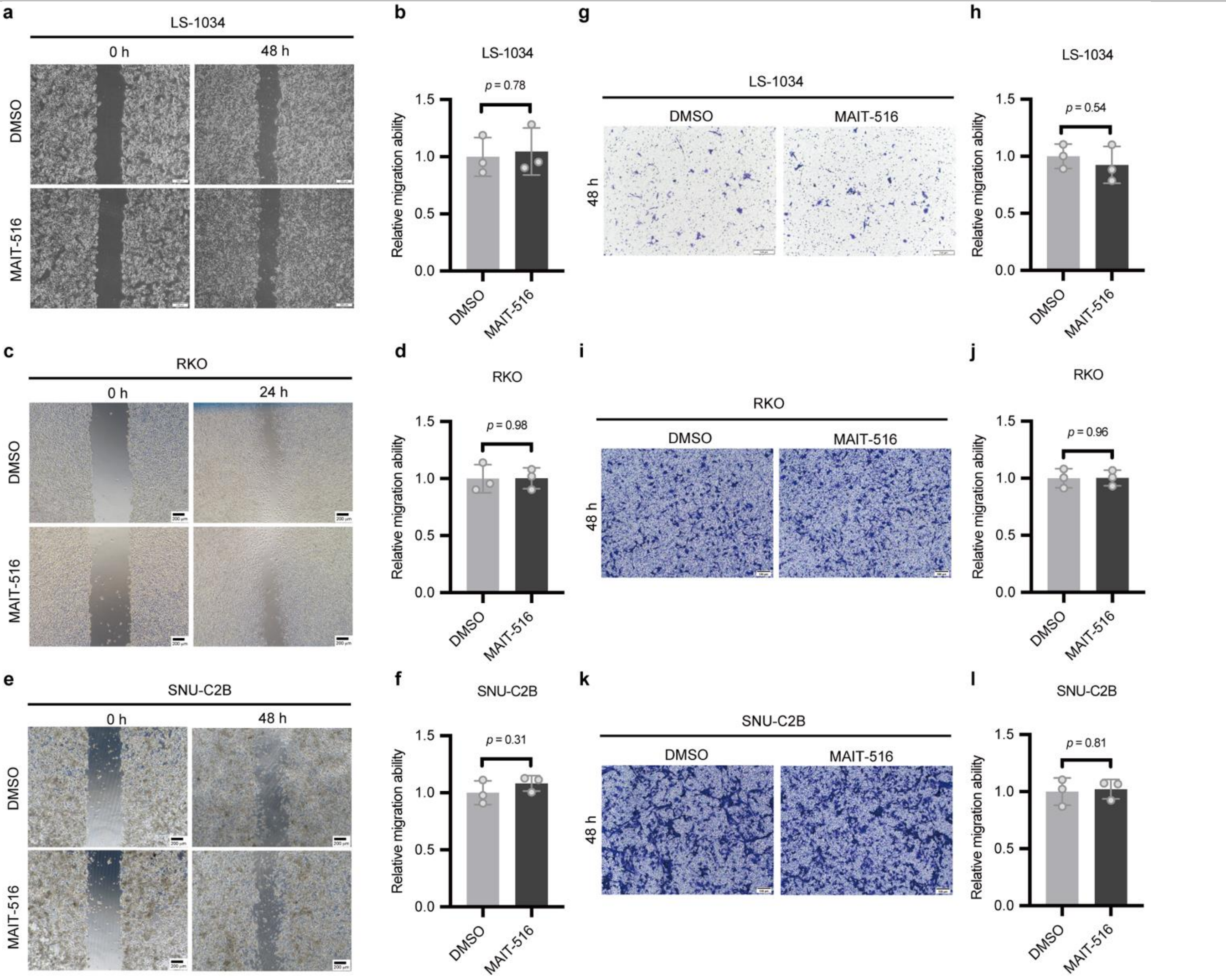

Supplementary Fig. 12. Effect of MAIT-516 on cell migration of colorectal cancer cells expressed full-length APC evaluated by wound healing and transwell migration assay. a-f) Representative images from wound healing assay of LS-1034 (a), RKO (c) and SNU-C2B (e) treated with MAIT-516 (25 μM) for indicated time. Quantitative data of cell migration are expressed relative to the migration ability of DMSO-treated group (b, d, f). Scale bar = 200 μm. g-l) Representative images of migrated LS-1034 (g), RKO (i) and SNU-C2B (k) cells stained with crystal violet are displayed. Cells were placed in the upper chamber of transwell inserts and treated with MAIT-516 (25 μM) for 48 h. Quantitative data of cell migration are expressed as relative to the migration ability of DMSO-treated group (h, j, l). Data are represented as the mean ± SD (n = 3 independent experiments). Scale bar = 100 μm. *p* values were calculated by two-tailed unpaired Student's t test. Source data are provided as a Source Data file.

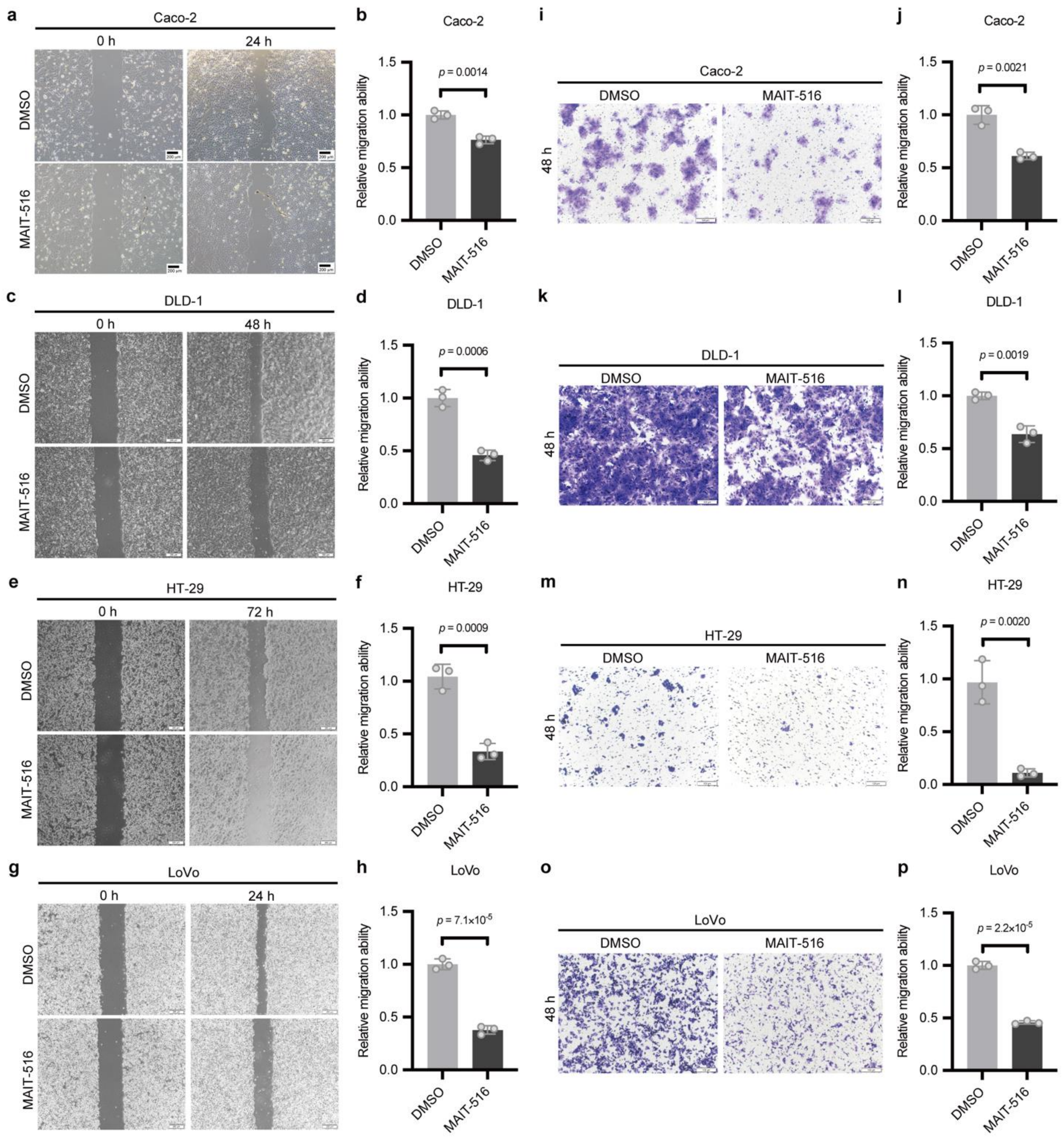

Supplementary Fig. 13. Effect of MAIT-516 on cell migration of colorectal cancer cells expressed truncated APC evaluated by wound healing and transwell migration assay. a-h) Representative images from wound healing assay of Caco-2 (a), DLD-1 (c), HT-29 (e) and LoVo (g) treated with MAIT-516 (25 μM) for indicated time. Quantitative data of cell migration are expressed relative to the migration ability of DMSO-treated group (b, d, f, h). Scale bar = 200 μm. i-p) Representative images of migrated Caco-2 (i), DLD-1 (k), HT-29 (m) and LoVo (o) cells stained with crystal violet are displayed. Cells were placed in the upper chamber of transwell inserts and treated with MAIT-516 (25 μM) for 48 h. Quantitative data of cell migration are expressed as relative to the migration ability of DMSO-treated group (j, l, n, p). Data are represented as the mean ± SD (n = 3 independent experiments). Scale bar = 100 μm. *p* values were calculated by two-tailed unpaired Student‘s t test. Source data are provided as a Source Data file.

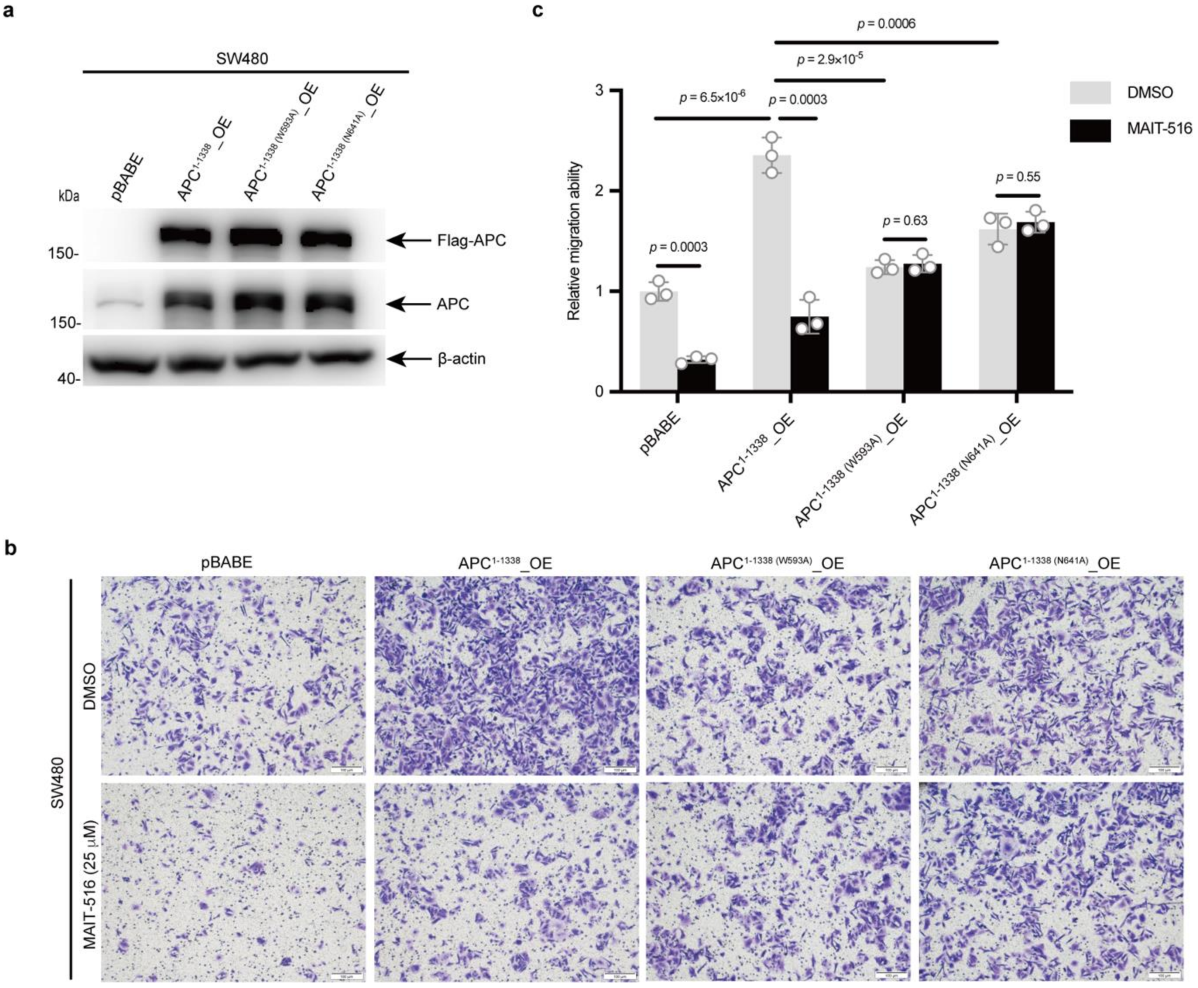

Supplementary Fig. 14. Effect of MAIT-516 on transwell migration of SW480 stable cells expressing FLAG-tagged APC 1-1338 and W593A and N641 mutants. a) SW480 cells were stably transfected with FLAG-tag expressing vector pBABE puro (pBABE), APC 1-1338 (APC^{1-1338}_OE), W593A ($APC^{1-1338(W593A)}$_OE) and N641A ($APC^{1-1338(N641A)}$_OE) and confirmed by western blot analysis with anti-FLAG and anti-APC antibody. Full blots are provided in Source Data files. The experiments were repeated three times with similar results (n = 3 independent experiments). b) Representative images of migrated cells stained with crystal violet are displayed. Cells were placed in the upper chamber of transwell inserts and treated with MAIT-516 (25 μM) for 48 h. The experiments were repeated three times with similar results (n = 3 independent experiments). c) Quantitative data of cell migration are expressed relative to the migration ability of DMSO-treated group. Data are represented as the mean ± SD (n = 3 independent experiments). *p* values were calculated by two-tailed unpaired Student's t test when comparing the migration ability between DMSO and MAIT-516 at the same APC status, or by one-way ANOVA with Tukey's multiple comparisons test when comparing the migration ability among different APC statuses. Scale bar = 100 μm. Source data are provided as a Source Data file.

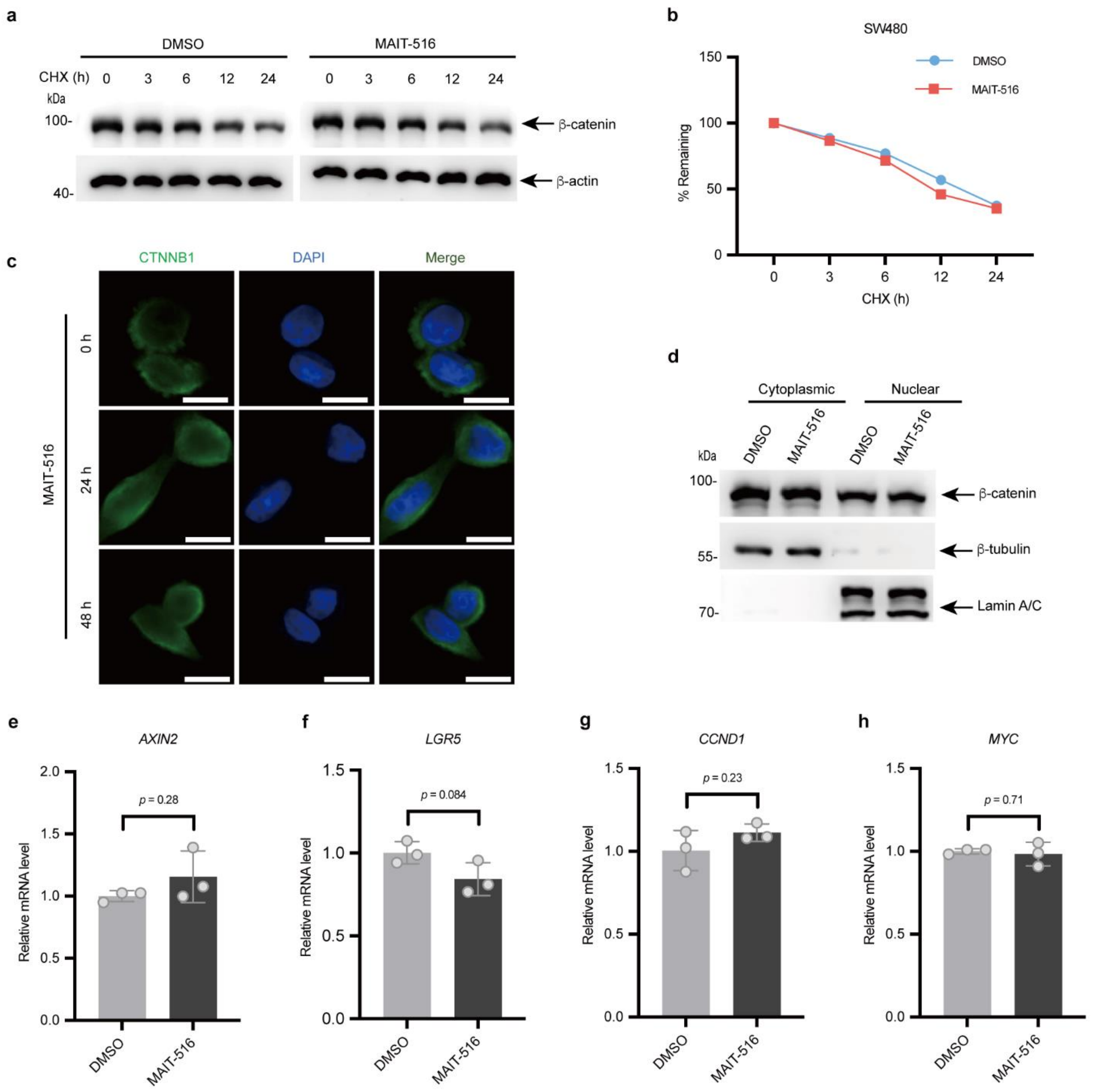

Supplementary Fig. 15. MAIT-516 does not affect the degradation, localization, nuclear translocation and transcriptional activity of β-catenin in SW480 cells. a, b) MAIT-516 does not affect the degradation of β-catenin. SW480 cells were pretreated with DMSO or 25 μM MAIT-516 for 24 h, and then treated with 10 mM cycloheximide (CHX) at indicated times. The expression of β-catenin were analyzed by Western blot (a). The intensity of β-catenin protein was quantified using Gel-Pro analyzer (b). c) MAIT-516 does not affect the localization of β-catenin in SW480 cells. Representative immunofluorescence images of the localization of β-catenin in SW480 cells treated with 25 μM MAIT-516 for 24 or 48 h. Scale bar, 10 μm. The experiments were repeated three times with similar results (n = 3 independent experiments). d) MAIT-516 does not affect the nuclear translocation of β-catenin in SW480 cells. Representative western blot analysis of β-catenin in subcellular fractionations of SW480 cells treated with 25 μM MAIT-516 for 48 h. The experiments were repeated three times with similar results (n = 3 independent experiments). e-h) MAIT-516 does not affect the transcriptional activity of β-catenin in SW480 cells. The mRNA level of *AXIN2*, *LGR5*, *CCND1* and *MYC* was analyzed by qPCR in SW480 cells treated with 25 μM MAIT-516 for 48 h, relative to the mRNA level of the DMSO control. Data are represented as the mean ± SD (n = 3 independent experiments). *p* values were calculated by two-tailed unpaired Student's t test. Full blots are provided in Source Data files.

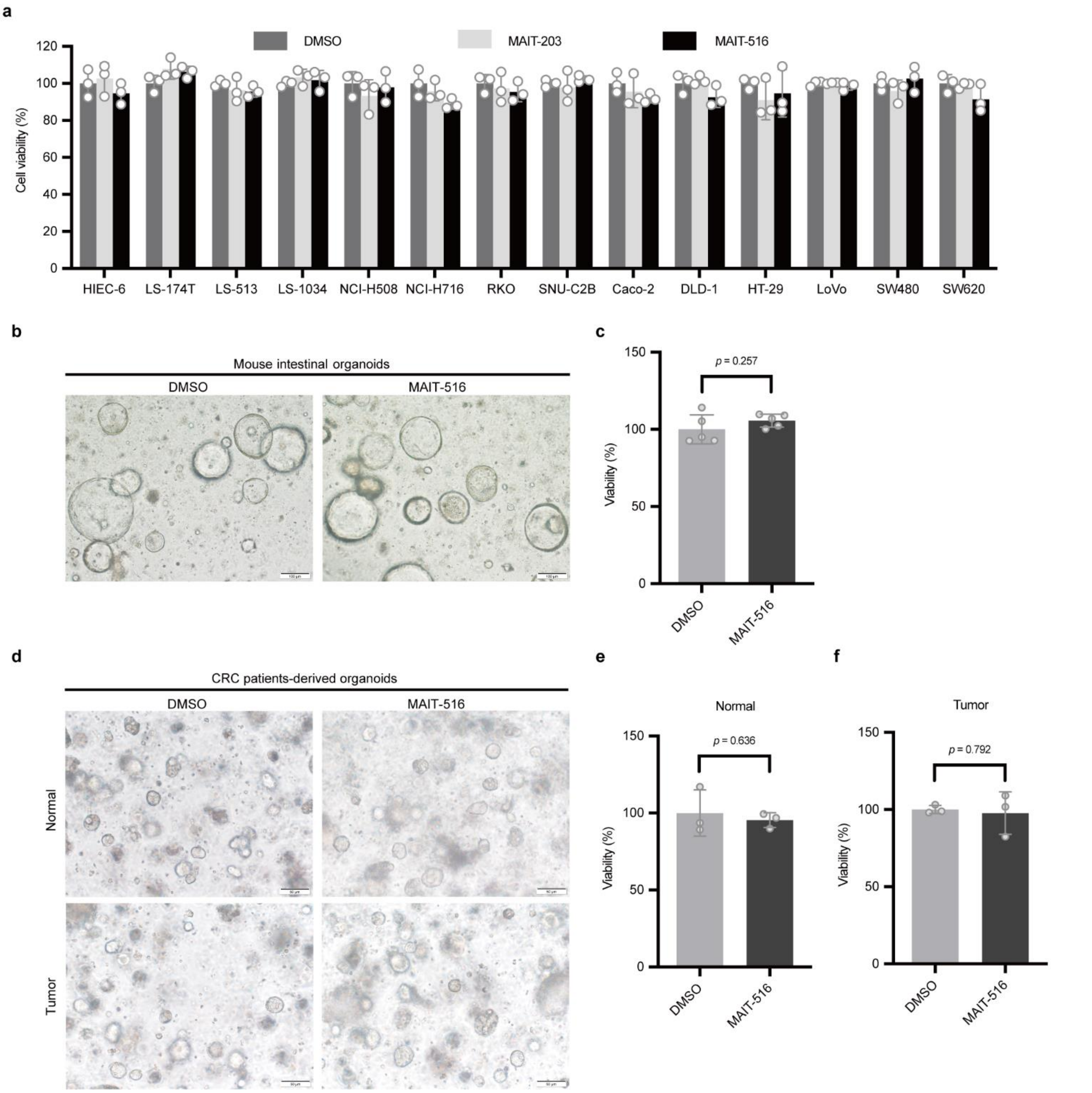

Supplementary Fig. 16. MAIT-516 does not affect cell viability. a) Cell viability of normal intestinal epithelial cell and colorectal cancer cells was determined by CCK-8 assays after treatment with DMSO, MAIT-203 (50 μM) or MAIT-516 (50 μM) for 48 h. Data are represented as the mean ± SD (n = 3 independent experiments). b,c) Representative photomicrographs (b) of mouse intestinal organoids treated with MAIT-516 (25 μM) for 48 h. Scale bar = 100 μm. Cell viability was measured by CellTiter-Glo assay. The relative cell viability was normalized to the DMSO control (c). Data are represented as the mean ± SD (n = 5 biologically independent samples). d-f) Representative photomicrographs (d) of CRC patients-derived organoids treated with MAIT-516 (25 μM) for 48 h. Scale bar = 50 μm. Cell viability was measured by CellTiter-Glo assay. The relative cell viability was normalized to the DMSO control (e, f). Data are represented as the mean ± SD (n = 3 independent experiments). *p* values were calculated by two-tailed unpaired Student's t test. The experiment was repeated twice. Source data are provided as a Source Data file.

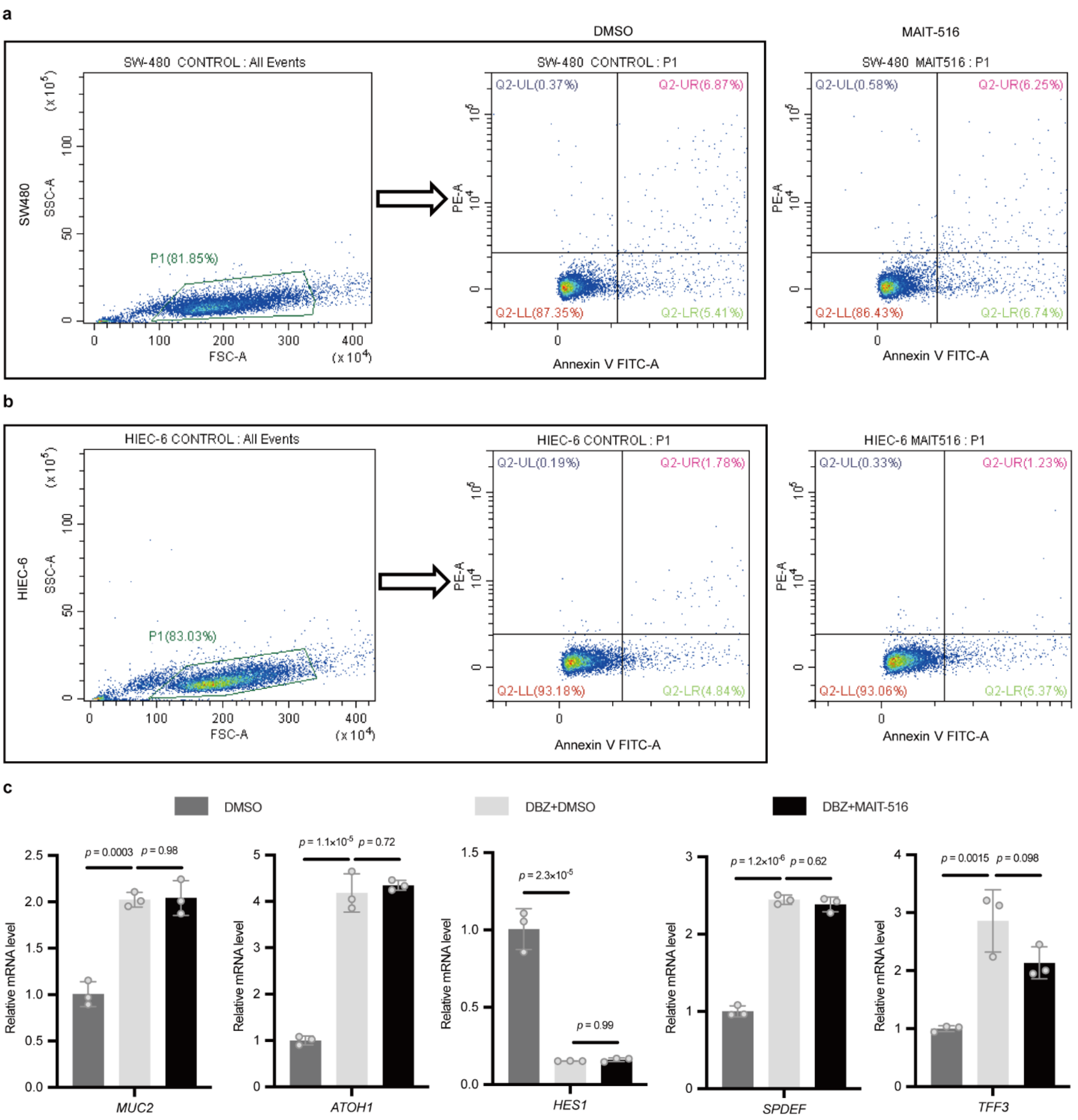

Supplementary Fig. 17. Effect of MAIT-516 on apoptosis and cell differentiation. a,b) Flow cytometry apoptosis analysis of SW480 (a) and HIEC-6 (b) cells treated with 25 μM MAIT-516 for 48 h, the gating strategy is shown for control sample. c) RT-PCR analysis of genes involved in intestinal cell differentiation (*MUC2*, *ATOH1*, *HES1*, *SPDEF* and *TFF3*) in LS-174T cells. LS-174T cells were treated with 1 μM DBZ for 1 day and followed by co-treatment with DMSO or 25 μM MAIT-516 for another 2 days. Data are represented as the mean ± SD (n = 3 independent experiments). *p* values were calculated by one-way ANOVA with Tukey's multiple comparisons test. Source data are provided as a Source Data file.

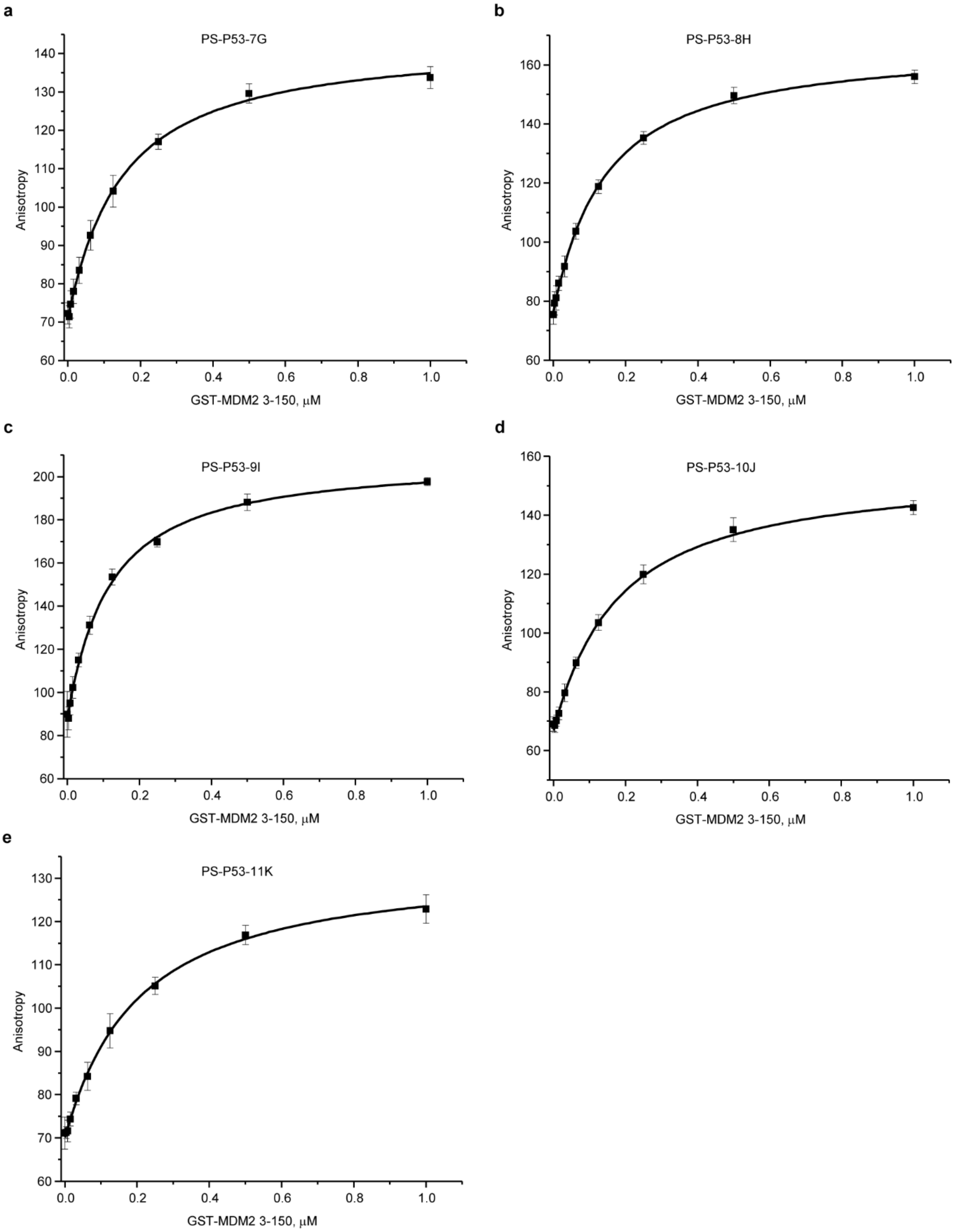

Supplementary Fig. 18. The binding curves of five tracers to GST-MDM2 3-150. a-e) The direct binding curves of tracers PS-P53-7G (a), PS-P53-8H (b), PS-P53-9I (c), PS-P53-10J (d), and PS-P53-11K (e). Data are represented as the mean ± SD. The experiments were performed in triplicates and repeated for three times (n = 3 independent experiments).

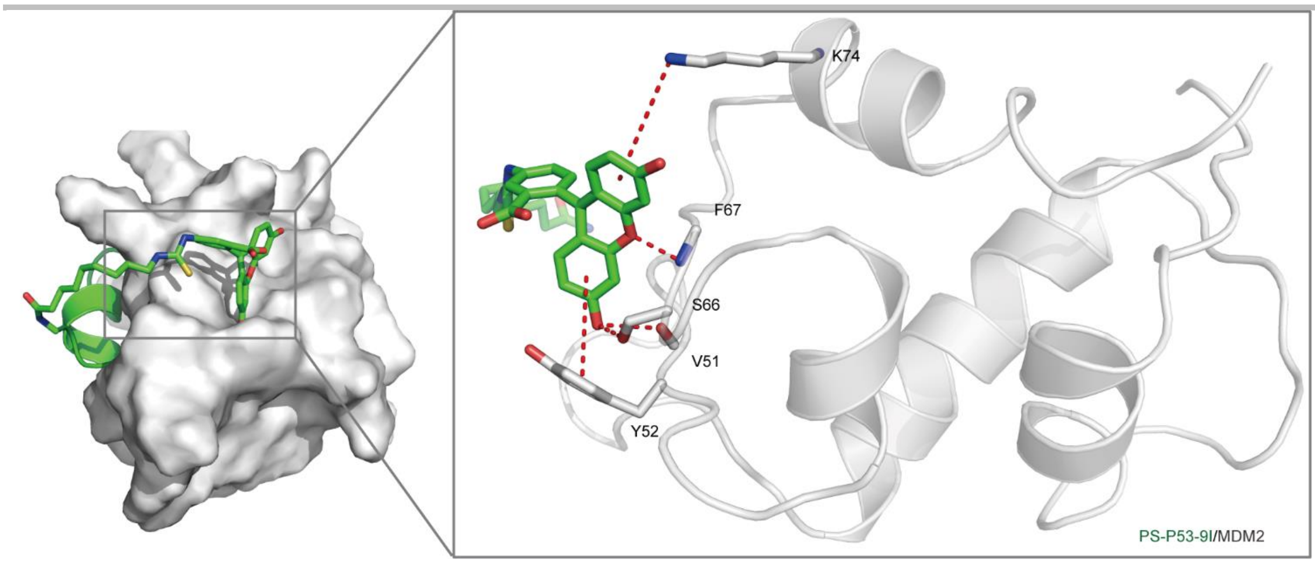

Supplementary Fig. 19. The significant conformation of PS-P53-9I to MDM2 by molecular dynamics simulations. MDM2 is shown as a solvent-accessible surface (gray), and PS-P53-9I is depicted by sticks (green carbon atoms). The red dashed lines represent the interactions between PS-P53-9I (green carbon atoms) and MDM2 (gray carbon atoms).

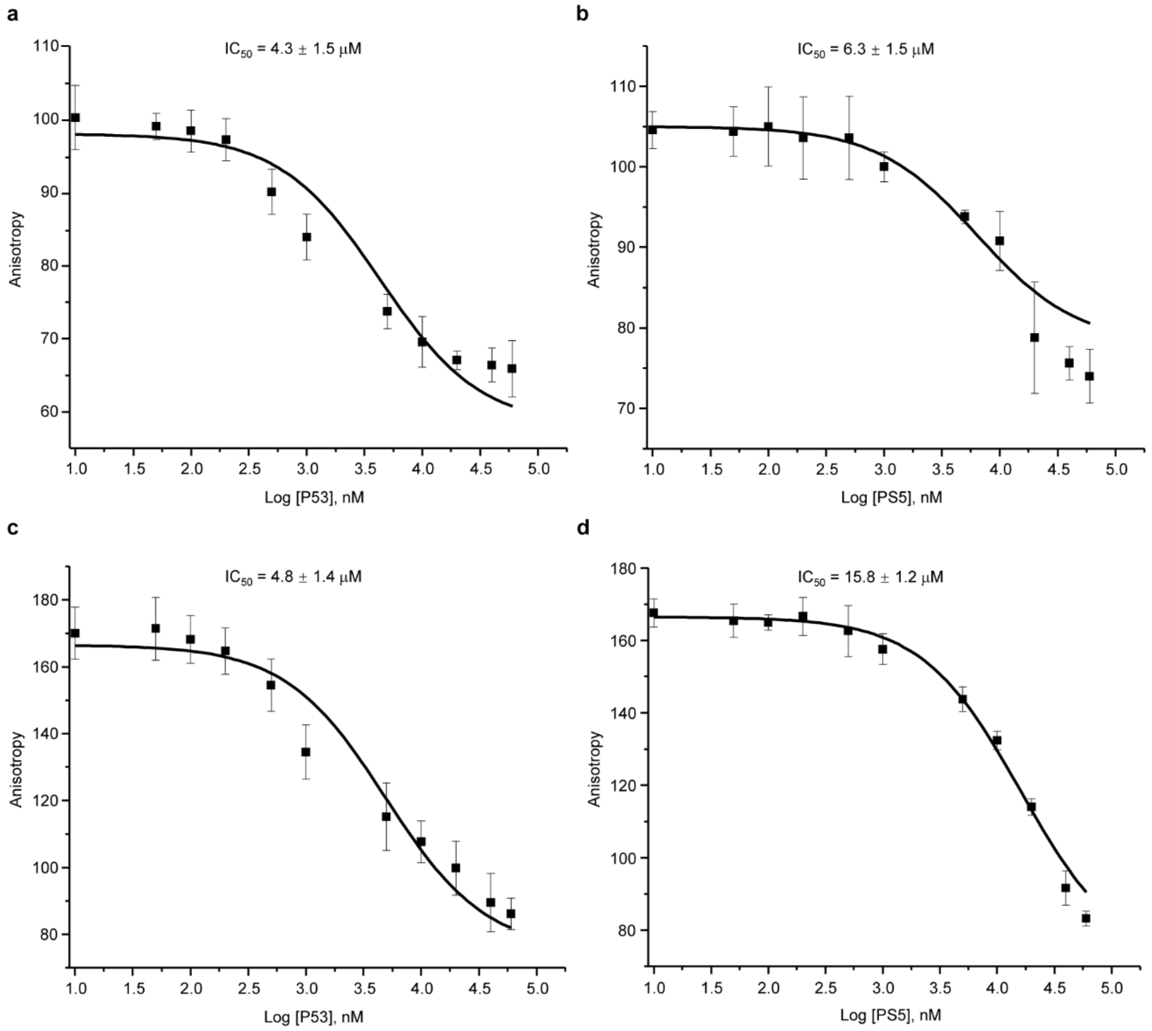

Supplementary Fig. 20. Tracer PS-P53-9I is more sensitive in differentiating peptides P53 and PS5 in FP assay. a, b) The dose-response curves of peptides P53 (a) and PS5 (b) were measured by tracer PS-P53-11K in FP assay. c, d) The dose-response curves of peptides P53 (c) and PS5 (d) were measured by tracer PS-P53-9I in FP assay. Data are represented as the mean ± SD. The experiments were performed in triplicates and repeated for three times (n = 3 independent experiments). Source data are provided as a Source Data file.

Supplementary Table 1. The binding affinity and dynamic range for tracer 8–14.

Tracer	Sequence	EC_{50} [nM][a]	Dynamic Range [Δmp]
8	Z-AGESLYENELISK[FITC]-NH_2	41 ± 0.7	309 ± 2.7
9	Z-AGESLYENELK[FITC]-NH_2	47 ± 1.5	295 ± 4.2
10	Z-AGESLYENK[FITC]-NH_2	52 ± 0.7	308 ± 2.0
11	Z-AGESLYEGGGGGGGK[FITC]-NH_2	53 ± 1.3	288 ± 3.3
12	Z-AGESLYEGGGGGK[FITC]-NH_2	51 ± 1.1	296 ± 3.0
13	Z-AGESLYEGGGK[FITC]-NH_2	48 ± 1.1	289 ± 3.0
14	Z-AGESLYEGK[FITC]-NH_2	50 ± 0.9	308 ± 2.5

[a] The effects of the tracers in the fluorescence-polarization (FP) competition assay were assessed as described in the Experimental Section. Data are presented as the mean ± s.e.m. (n = 2 independent experiments). The experiments were performed in triplicates and repeated twice. Source data are provided as a Source Data file.

Supplementary Table 2. Thermodynamic data for the binding of tracer 1 and tracer 7 to APC.

Compd.	Protein	L/P ratio	Kd (μM)	ΔG (kJ mol^{-1})	ΔH (kJ mol^{-1})	-TΔS (kJ mol^{-1})*
tracer 1	APC WT	1.2	0.58	-36.2	-55.9	19.7
tracer 7	APC WT	1.6	0.078	-41.2	-46.4	5.2

[a] All experiments were performed at 30 °C. The L/P ratio indicates the number of sites per APC. *ΔH*: change in enthalpy; -*TΔS*: change in entropy; K_d: equilibrium dissociation constant determined by ITC.

Supplementary Table 3. Data collection and refinement statistics for co-crystals of APC–tracer 7 and APC–MAI-516.

	APC–MAI-516	APC–tracer 7
**Data collection**		
Space group	C 1 2 1	P 21 21 21
Cell dimensions		
a, b, c (Å)	87.75, 82.34, 49.74	57.23, 71.62, 83.87
α, β, γ (°)	90.00, 94.69, 90.00	90.00, 90.00, 90.00
Resolution (Å)	50-2.40 (2.44-2.40)	50-2.80 (2.85-2.80)
R_{sym} or R_{merge}	0.167 (0.496)	0.127 (0.641)
$I / \sigma I$	9.8 (2.4)	3.4 (2.2)
Completeness (%)	99.2 (99.9)	99.4 (96.6)
Redundancy	7.0 (7.0)	5.8 (5.9)
**Refinement**		
Resolution (Å)	2.40	2.80
No. reflections	96055	51944
R_{work} / R_{free}	0.208 / 0.256	0.285 / 0.328
No. atoms	2861	2647
Protein	2646	2525
Ligand	64	101
Solvent	54	/
Water	97	21
B-factors	38.918	48.175
Protein	39.047	48.825
Ligand	27.242	36.378
Solvent	51.805	/
Water	35.934	26.813
R.m.s. deviations		
Bond lengths (Å)	0.007	0.007
Bond angles (°)	1.049	0.951
Ramachandran plot statistics (%)		
Favoured	92.8	93.1
Allowed	7.2	6.9
Disallowed	0.0	0.0
PDB ID	7F6M	7F7O

*Highest-resolution shell is shown in parentheses. Data were collected from one crystal for each structure.

Supplementary Table 4. The IC_{50} values of peptides with different tracers in FP assay.

Compounds	IC_{50} (μM)[a]	
	Tracer 1	Tracer 7
MAI-426	0.84 ± 0.19	1.2 ± 0.06
MAI-451	0.39 ± 0.12	0.27 ±0.005
MAI-452	0.44 ± 0.15	0.17 ± 0.010
MAI-453	0.46 ± 0.14	0.26 ± 0.010
MAI-454	0.48 ± 0.15	0.28 ± 0.010
MAI-455	0.53 ± 0.12	0.86 ± 0.03
MAI-456	0.57 ± 0.12	0.99 ± 0.06
MAI-457	0.59 ± 0.2	0.092 ± 0.003
MAI-458	0.59 ± 0.14	0.90 ± 0.03
MAI-459	0.60 ± 0.12	0.84 ± 0.03
MAI-460	0.62 ± 0.19	0.55 ± 0.009
MAI-461	0.63 ± 0.2	0.080 ± 0.003
MAI-462	0.73 ± 0.14	0.31 ±0.010
MAI-463	0.73 ± 0.15	1.3 ± 0.05
MAI-464	0.74 ± 0.16	0.22 ± 0.01
MAI-465	0.75 ± 0.15	1.0 ± 0.03
MAI-466	0.81 ± 0.2	0.94 ± 0.03
MAI-467	0.82 ± 0.3	0.32 ± 0.007
MAI-468	0.86 ± 0.19	1.4 ± 0.05
MAI-469	0.88 ± 0.2	0.83 ± 0.02
MAI-470	0.94 ± 0.16	2.0 ± 0.09
MAI-471	0.95 ± 0.2	1.7 ± 0.13
MAI-472	0.96 ± 0.16	1.8 ± 0.08
MAI-473	0.97 ± 0.14	1.8 ± 0.04
MAI-505	0.44 ± 0.12	1.6 ± 0.06
MAI-507	0.34 ± 0.12	0.17 ± 0.007
MAI-508	0.48 ± 0.16	0.22 ± 0.005
MAI-509	0.64 ± 0.17	0.59 ± 0.03
MAI-510	0.49 ± 0.13	0.37 ± 0.006
MAI-512	0.75 ± 0.18	1.0 ± 0.03
MAI-203	0.55 ± 0.06	2.0 ± 0.08

Source data are provided as a Source Data file.

Supplementary Table 5. The structure, HPLC, and MS data of compounds MAI-451–MAI-473.

Compounds	Structure	MS	HPLC
MAI-451	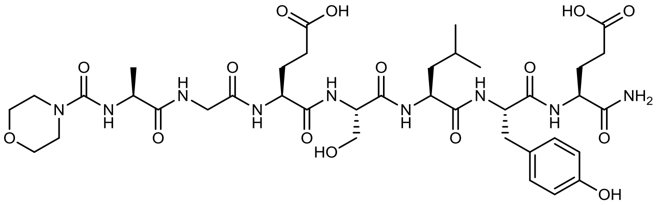	878.3 [M-H]$^-$	98.2%
MAI-452	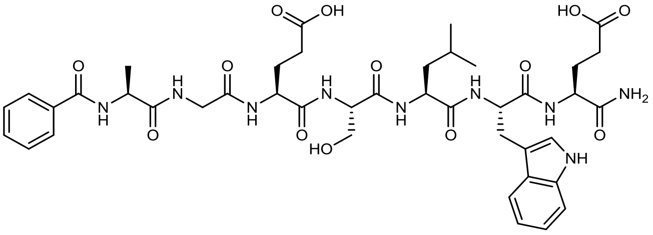	894.4 [M+H]$^+$	98.5%
MAI-453	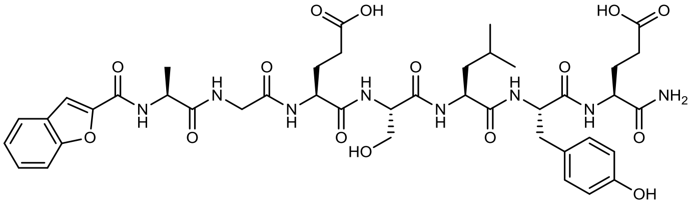	909.3 [M-H]$^-$	98.2%
MAI-454	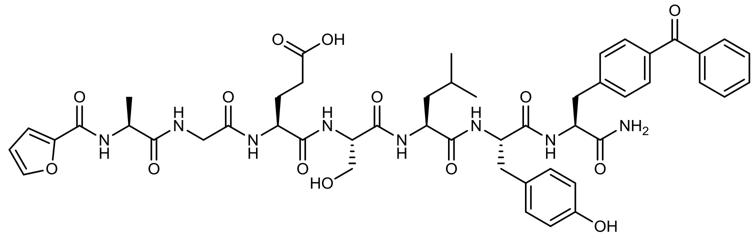	981.3 [M-H]$^-$	98.2%
MAI-455	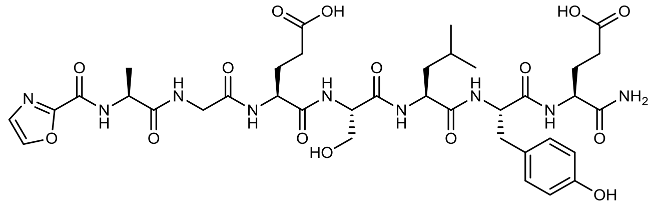	860.3 [M-H]$^-$	98.8%
MAI-456	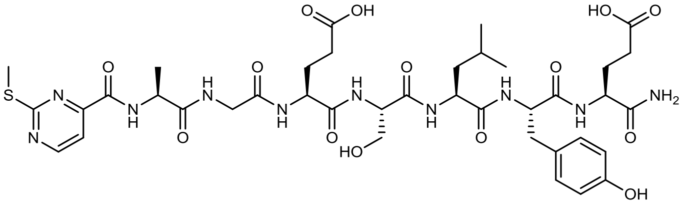	917.3 [M-H]$^-$	98.2%
MAI-457	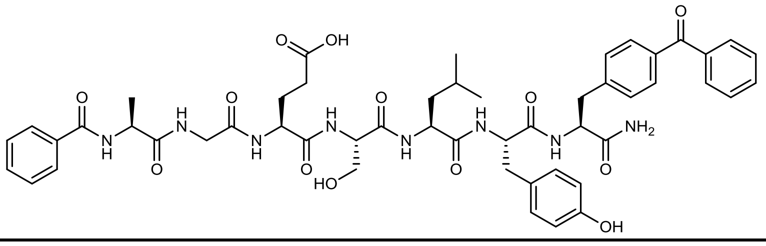	991.4 [M-H]$^-$	98.6%

MAI-458	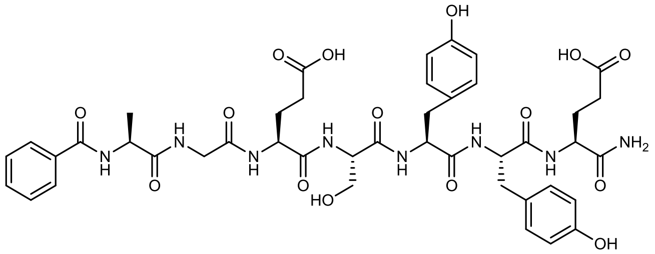	919.3 [M-H]$^{-}$	98.7%
MAI-459	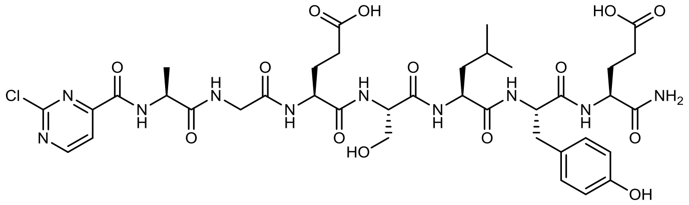	452.3 [M-2H]$^{2-}$	98.5%
MAI-460	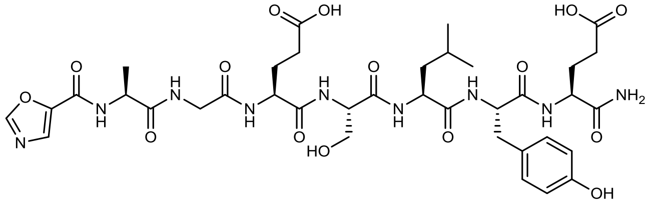	860.2 [M-H]$^{-}$	98.6%
MAI-461	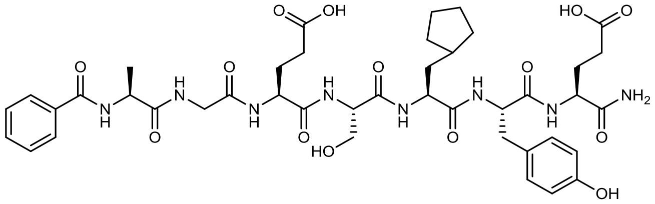	895.3 [M-H]$^{-}$	98.3%
MAI-462	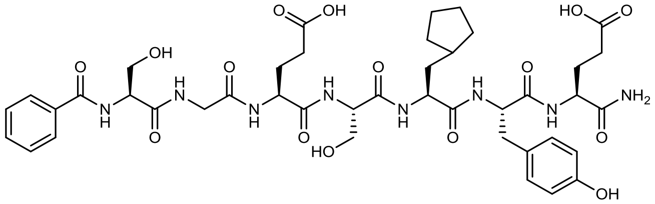	885.35 [M-H]$^{-}$	98.4%
MAI-463	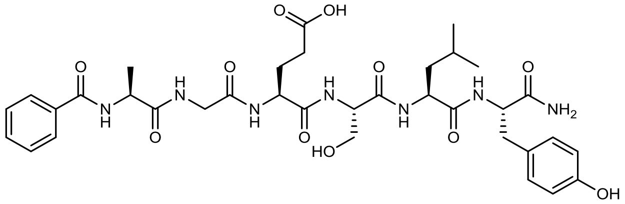	740.3 [M-H]$^{-}$	98.1%
MAI-464	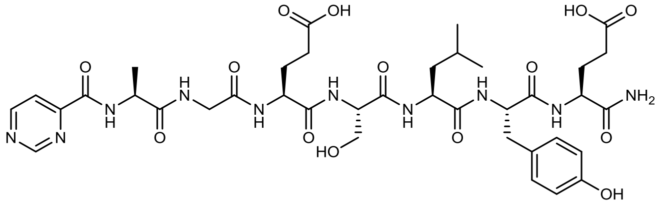	873.35 [M+H]$^{+}$	98.4%
MAI-465	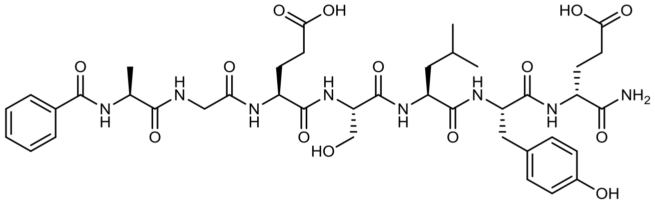	869.3 [M-H]$^{-}$	98.7%

MAI-466	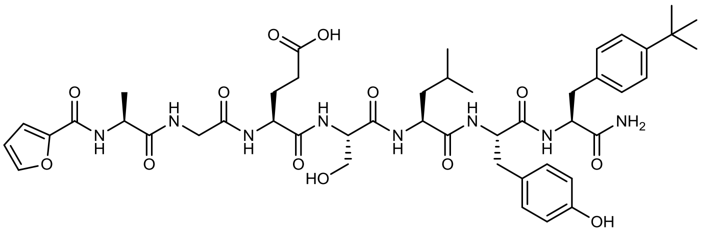	933.4 [M-H]⁻	98.2%
MAI-467	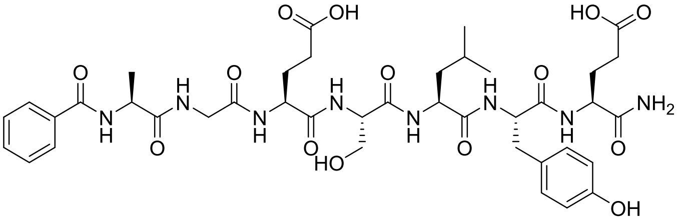	943.4 [M-H]⁻	98.2%
MAI-426	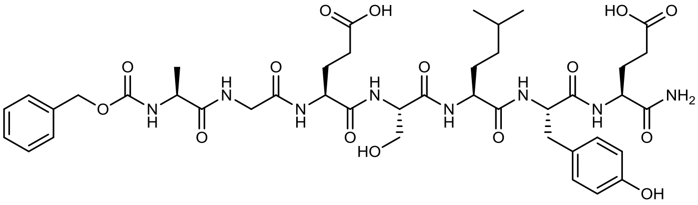	913.3 [M-H]⁻	98.4%
MAI-468	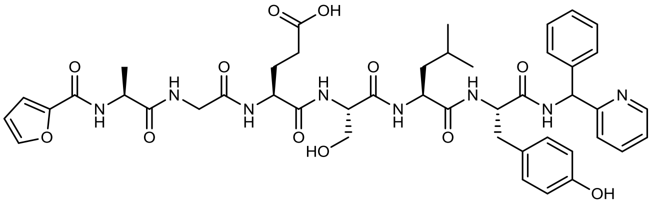	897.2 [M-H]⁻	98.6%
MAI-469	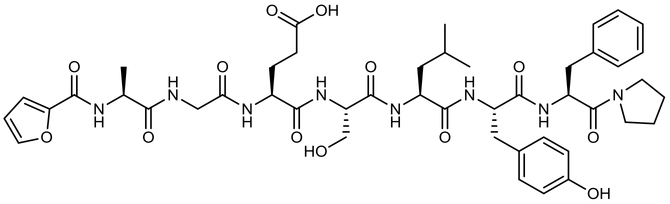	931.4 [M-H]⁻	98.2%
MAI-470	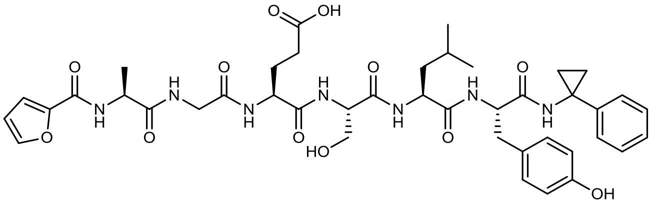	846.2 [M-H]⁻	98.3%
MAI-471	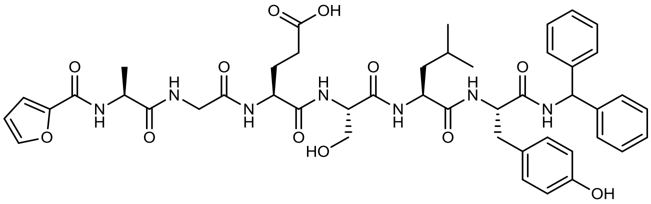	896.3 [M-H]⁻	98.4
MAI-472	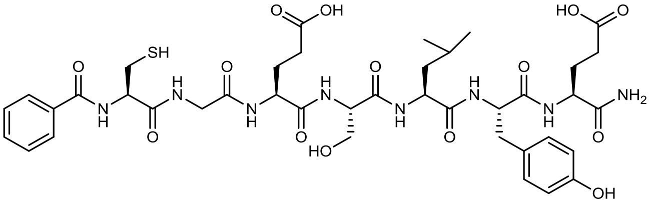	901.2 [M-H]⁻	98.6%

MAI-473	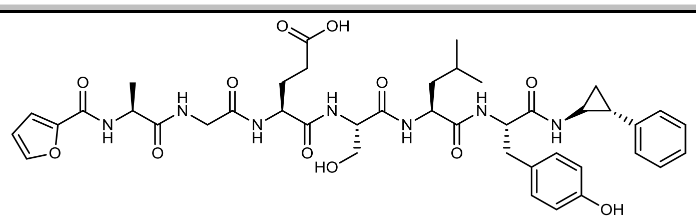	846.2 [M-H]$^-$	98.4%

Supplementary Table 6. The structure and binding affinity for tracers of MDM2.

Tracer	Structure	K_d (μM)[a]
PS-P53-7G	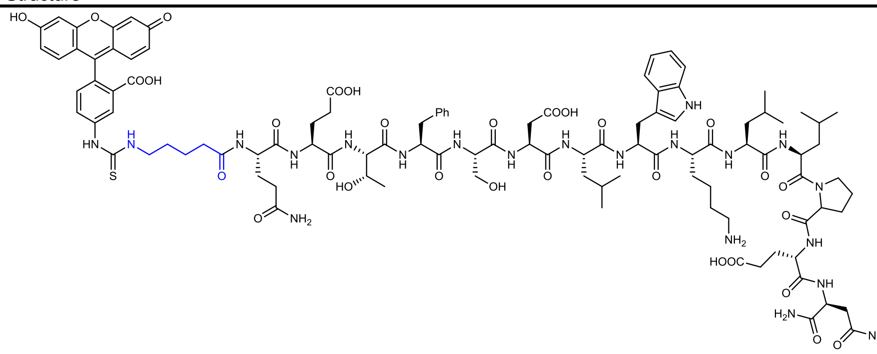	0.13 ± 0.011
PS-P53-8H	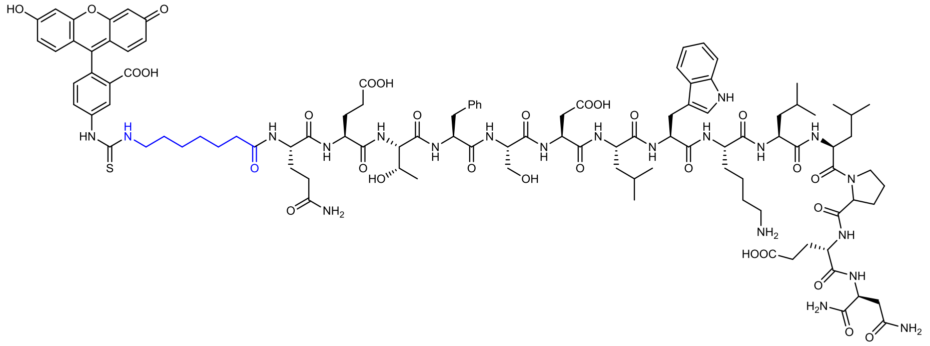	0.13 ± 0.006
PS-P53-9I	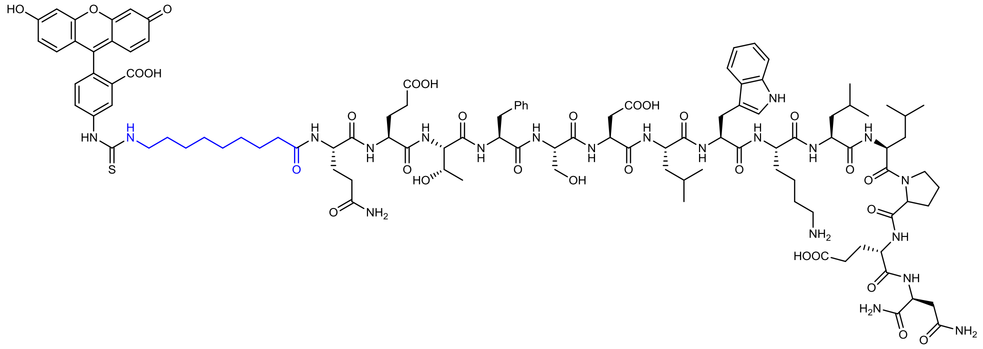	0.10 ± 0.008
PS-P53-10J	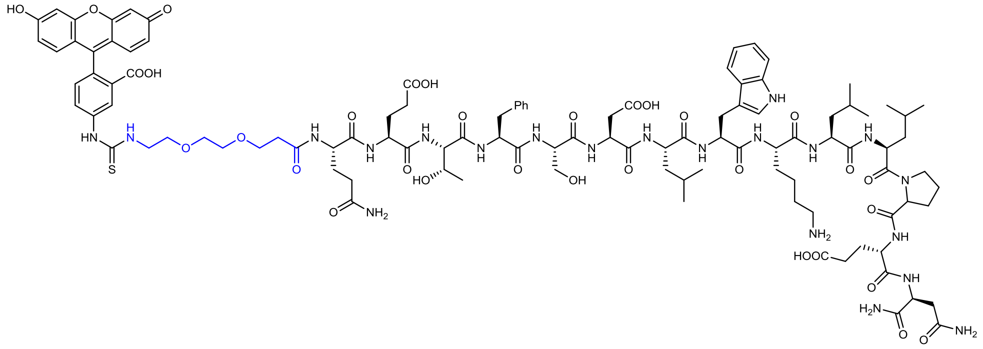	0.16 ± 0.010
PS-P53-11K	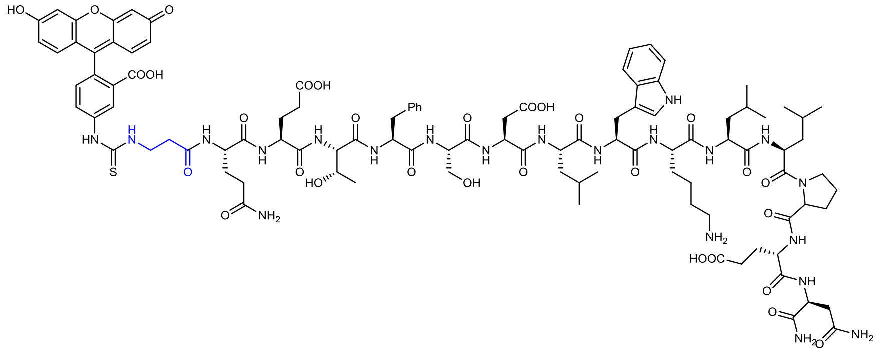	0.18 ± 0.014

[a] The binding affinity (K_d) of peptides was obtained by fluorescence polarization (FP). The experiments were performed in triplicates and repeated for three times (n = 3 independent experiments). Source data are provided as a Source Data file.

Supplementary Table 7. The IC_{50} values of peptides to MDM2 with different tracers in FP assay.

Tracer	IC_{50} (μM)[a]	
	P53	PS5
PS-P53-11K	4.3 ± 1.5	6.3 ± 1.5
PS-P53-9I	4.8 ± 1.4	16 ± 1.2

[a] The IC_{50} of peptides was obtained by incubating sulfono-γ-AA peptides PS5 and P53 from 0.01 μM to 60 μM with 25 nM competitor PS-P53-11K or PS-P53-9I and 250 nM GST-MDM2-3-150. The experiments were performed in triplicates and repeated for three times (n = 3 independent experiments). Source data are provided as a Source Data file.

Supplementary Table 8. Sequence of site directed mutagenesis and real-time PCR primers used in the study.

Gene	Forward Sequence 5' to 3'	Reverse Sequence 5' to 3'
APC 303-739 W593A	AGCGTATTGAGTGCCTTAGCGAATTTGTCAGCACATTG	CAATGTGCTGACAAATTCGCTAAGGCACTCAATACGCT
APC 303-739 N641A	GTGGGATATTACGG**GC**TGTGTCCAGCTTGATAGCT	GGACACAGCCCGTAATATCCCACCTCCACTTTCAA
h-β-ACTIN	GACCTGTACGCCAACACAG	CTCAGGAGGAGCAATGATC
h-AXIN2	CTGGCTTTGGTGAACTGTTG	AGTTGCTCACAGCCAAGACA
h-LGR5	CTCTTCCTCAAACCGTCTGC	GATCGGAGGCTAAGCAACTG
h-CCND1	GCTGCGAAGTGGAAACCATC	CCTCCTTCTGCACACATTTGAA
h-MYC	CTTCTCTGAAAGGCTCTCCTTGC	CGAGGTCATAGTTCCTGTTGGTG
h-MUC2	GACACCATCTACCTCACCCG	TGTAGGCATCGCTCTTCTCA
h-ATOH1	GCAGGAGGAAAACAGCAAAA	ACTTGCCTCATCCGAGTCAC
h-HES1	AGCACACTTGGGTCTGTGC	TGAAGAAAGATAGCTCGCGG
h-SPDEF	GCACTGCAGCAGACAGCTC	GGGGATACGCTGCTCAGAC
h-TFF3	CTCCAGCTCTGCTGAGGAGT	CAGGGATCCTGGAGTCAAAG

Supplementary References

1. Zhang, Z.Y., *et al.* Structural basis for the recognition of Asef by adenomatous polyposis coli. *Cell Res* **22**, 372-386 (2012).

2. Ni, D., *et al.* Discovery of cryptic allosteric sites using reversed allosteric communication by a combined computational and experimental strategy. *Chem Sci* **12**, 464-476 (2020).

3. Kussie, P.H., *et al.* Structure of the MDM2 oncoprotein bound to the p53 tumor suppressor transactivation domain. *Science* **274**, 948-953 (1996).
