# Supplementary material for: Rational design of a sensitivity-enhanced tracer for discovering efficient APC–Asef inhibitors
Source: Nat Commun. 2022 Aug 24;13:4961. doi: 10.1038/s41467-022-32612-6 (PMC9402538; doi:10.1038/s41467-022-32612-6)
Supplement: Supplementary file 3 — Reporting summary [file 41467_2022_32612_MOESM3_ESM.pdf]

nature portfolio

Corresponding author(s): Jian Zhang, Xiuyan Yang and Chen-ying Liu

Last updated by author(s): Jul 28, 2022

Reporting Summary

Nature Portfolio wishes to improve the reproducibility of the work that we publish. This form provides structure for consistency and transparency in reporting. For further information on Nature Portfolio policies, see our Editorial Policies and the Editorial Policy Checklist.

Statistics

For all statistical analyses, confirm that the following items are present in the figure legend, table legend, main text, or Methods section.

n/a	Confirmed	
☐	☒	The exact sample size (*n*) for each experimental group/condition, given as a discrete number and unit of measurement
☐	☒	A statement on whether measurements were taken from distinct samples or whether the same sample was measured repeatedly
☐	☒	The statistical test(s) used AND whether they are one- or two-sided<br>*Only common tests should be described solely by name; describe more complex techniques in the Methods section.*
☒	☐	A description of all covariates tested
☒	☐	A description of any assumptions or corrections, such as tests of normality and adjustment for multiple comparisons
☐	☒	A full description of the statistical parameters including central tendency (e.g. means) or other basic estimates (e.g. regression coefficient) AND variation (e.g. standard deviation) or associated estimates of uncertainty (e.g. confidence intervals)
☐	☒	For null hypothesis testing, the test statistic (e.g. *F*, *t*, *r*) with confidence intervals, effect sizes, degrees of freedom and *P* value noted<br>*Give P values as exact values whenever suitable.*
☒	☐	For Bayesian analysis, information on the choice of priors and Markov chain Monte Carlo settings
☒	☐	For hierarchical and complex designs, identification of the appropriate level for tests and full reporting of outcomes
☒	☐	Estimates of effect sizes (e.g. Cohen's *d*, Pearson's *r*), indicating how they were calculated

Our web collection on statistics for biologists contains articles on many of the points above.

Software and code

Policy information about availability of computer code

Data collection	Data was collected using the softwares provided by the respective instrument vendors which were specified in the Methods section.
Data analysis	Data was analysed with GraphPad Prism 7.0 (GraphPad Software Inc.), Biacore Insight Evaluation Software version 2.0.15.12933 (Cytiva Inc.), MicroCal Analysis Software (Malvern Panalytical Inc.), RTCA software 2.3 (Agilent Inc.), IBM SPSS Statistics 24 (IBM Inc.), ImageJ 1.53e(NIH), Adobe Illustrator CC 2018 (Adobe Inc.), Microsoft excel 2019 (Microsoft Inc.), HKL-2000 (HKL Research Inc.), PyMOL 2.4.1 (Schrodinger), Discovery Studio 2.5 (Dassault Systemes), Beckman CytExpert 1.2 (Beckman Coulter Inc.) and FlowJo 10 (Becton Dickinson Company).

For manuscripts utilizing custom algorithms or software that are central to the research but not yet described in published literature, software must be made available to editors and reviewers. We strongly encourage code deposition in a community repository (e.g. GitHub). See the Nature Portfolio guidelines for submitting code & software for further information.

Data

Policy information about availability of data

All manuscripts must include a data availability statement. This statement should provide the following information, where applicable:

- Accession codes, unique identifiers, or web links for publicly available datasets
- A description of any restrictions on data availability
- For clinical datasets or third party data, please ensure that the statement adheres to our policy

The structural data that support the findings in this study have been deposited in the Protein Data Bank with the coordinate accession numbers 7F6M (http://doi.org/10.2210/pdb7F6M/pdb) and 7F7O (http://doi.org/10.2210/pdb7F7O/pdb). The previously published structural data used in this study are available in the

Protein Data Bank under accession code 3NMZ (http://doi.org/10.2210/pdb3NMZ/pdb), 1YCR (http://doi.org/10.2210/pdb1ycr/pdb), and 5Z8H (http://doi.org/10.2210/pdb5Z8H/pdb). Uncropped blots and gels are provided in Source Data files. All other data generated or analyzed in this study are included in this article and its Supplementary Information file. Source data are provided with this paper.

Human research participants

Policy information about studies involving human research participants and Sex and Gender in Research.

Reporting on sex and gender	Our study did not involve human participants.
Population characteristics	Not relevant to our study.
Recruitment	Not relevant to our study.
Ethics oversight	Not relevant to our study.

Note that full information on the approval of the study protocol must also be provided in the manuscript.

Field-specific reporting

Please select the one below that is the best fit for your research. If you are not sure, read the appropriate sections before making your selection.

- [x] Life sciences
- [ ] Behavioural & social sciences
- [ ] Ecological, evolutionary & environmental sciences

For a reference copy of the document with all sections, see nature.com/documents/nr-reporting-summary-flat.pdf

Life sciences study design

All studies must disclose on these points even when the disclosure is negative.

Sample size	No statistical methods were used to predetermined sample size. Sample sizes were determined from similar studies(Jiang et al. Nat Chem Biol, 2017; Yang et al. J Med Chem, 2018). Three independent experiments were performed for all cellular assays and dose-response assays of APC-Asef PPI inhibitors.
Data exclusions	No data was excluded.
Replication	Each experiment was repeated at least two times. Replication times are detailed in each table and figure legend. All attempts of replications of individual experiment were successful.
Randomization	Mice were randomly divided into experimental groups. All other samples collected were used for this study without any discrimination.
Blinding	Blinding was not performed in most of the experiments of this study, as experimental observations would be consistent irrespective of blinding. Investigators who performed animal experiments were not blinded because they needed to prepare the drug. But the Investigators were blinded during the sample collection and data analysis.

Reporting for specific materials, systems and methods

We require information from authors about some types of materials, experimental systems and methods used in many studies. Here, indicate whether each material, system or method listed is relevant to your study. If you are not sure if a list item applies to your research, read the appropriate section before selecting a response.

Materials & experimental systems

n/a	Involved in the study	
[ ]	[x]	Antibodies
[ ]	[x]	Eukaryotic cell lines
[x]	[ ]	Palaeontology and archaeology
[ ]	[x]	Animals and other organisms
[x]	[ ]	Clinical data
[x]	[ ]	Dual use research of concern

Methods

n/a	Involved in the study	
[x]	[ ]	ChIP-seq
[ ]	[x]	Flow cytometry
[x]	[ ]	MRI-based neuroimaging

Antibodies

Antibodies used	Anti-FLAG M2 affinity gel, from Sigma, cat. no. A2220, lot no. SLBW1929

	Anti-HA monoclonal antibody produced in mouse, clone HA-7, from Sigma, cat. no. H9658, dil 1/20,000.<br>Anti-FLAG monoclonal antibody produced in mouse, clone M2, from Sigma, cat. no. F1804, dil 1/1,000.<br>Anti-Asef polyclonal antibody produced in Rabbit, from Proteintech, cat. no. 55213-1-AP, dil 1/1,000.<br>Anti-APC monoclonal antibody produced in mouse, clone Ali12.28, from Merck, cat. no. MAB3785, dil 1/500.<br>Anti-β-catenin monoclonal antibody produced in Rabbit, from CST, cat. no.D10A8, dil 1/1,000.<br>Anti-β-Tubulin monoclonal antibody produced in Rabbit, from CST, cat.n.2128, dil 1/1,000.<br>Anti-LaminA/C monoclonal antibody produced in Rabbit, from CST, cat.n.4777, dil 1/1,000.<br>HRP-conjugated Anti-Beta Actin monoclonal antibody, clone 7D2C10, from Proteintech Group Inc., cat. no. HRP-60008, dil 1/5,000.<br>Goat anti-Mouse IgG secondary antibody HRP conjugated, from Signalway Antibody LLC, cat. no. L3032, lot no. 6229, dil 1/50,000.<br>Goat anti-Rabbit IgG (H+L) Cross-Adsorbed Secondary Antibody, Alexa Fluor™ 488 (Invitrogen A11008,1:300)
Validation	The commercial antibodies employed in this study were validated by the manufacturers and used according to the manufacturers' instructions.Their validation data are available on the manufacturers websites, as listed below:<br>HA monoclonal antibody produced in mouse, clone HA-7, from Sigma, cat. no. H9658.<br>https://www.sigmaaldrich.cn/CN/zh/product/sigma/h9658<br>FLAG monoclonal antibody produced in mouse, clone M2, from Sigma, cat. no. F1804.<br>https://www.sigmaaldrich.cn/CN/zh/product/sigma/f1804<br>Asef polyclonal antibody produced in Rabbit, from Proteintech, cat. no. 55213-1-AP.<br>https://www.ptgcn.com/products/ARHGEF4-Antibody-55213-1-AP.htm<br>APC monoclonal antibody produced in mouse, clone Ali12.28, from Merck, cat. no. MAB3785.<br>https://www.sigmaaldrich.cn/CN/zh/product/mm/mab3785<br>β-catenin monoclonal antibody produced in Rabbit, from CST, cat. no.D10A8.<br>https://www.cellsignal.cn/products/primary-antibodies/b-catenin-d10a8-xp-rabbit-mab/8480?site-search-type=Products&N=4294956287&Ntt=d10a8&fromPage=plp<br>LaminA/C monoclonal antibody produced in Rabbit, from CST, cat.n.4777.<br>https://www.cellsignal.cn/products/primary-antibodies/lamin-a-c-4c11-mouse-mab/4777?site-search-type=Products&N=4294956287&Ntt=4777&fromPage=plp&_requestid=6872255

Eukaryotic cell lines

Policy information about cell lines and Sex and Gender in Research

Cell line source(s)	The HEK293T, HIEC-6, SW480, SW620, RKO, DLD-1, HT-29, LoVo, Caco-2 and LS-174T cell lines were originally purchased from the American Type Culture Collection (ATCC). The LS-513, LS-1034, NCI-H508, NCI-H716 and SNU-C2B cell lines were kindly provided by Prof. Meiyu Geng from the Shanghai Institute of Materia Medica and Prof. Lei Chen from the Eastern Hepatobiliary Surgery Institute. The original commercial source of LS-513, LS-1034, NCI-H508, NCI-H716 and SNU-C2B cell lines were the American Type Culture Collection (ATCC).
Authentication	Cell lines were authenticated using STR testing by Genetic Testing Biotechnology.
Mycoplasma contamination	All cell lines were negative for mycoplasma contamination.
Commonly misidentified lines (See ICLAC register)	No commonly misidentified cell lines were used.

Animals and other research organisms

Policy information about studies involving animals; ARRIVE guidelines recommended for reporting animal research, and Sex and Gender in Research

Laboratory animals	All the mice used in this study were four-week-old C57BL/6. Mice were in housed pathogen-free and ventilated cages with free access to food and water ad libitum under a 12:12 h light-dark cycle at room temperature of 21±2°C and humidity between 45% and 65%.
Wild animals	No wild animals were used in this study.
Reporting on sex	Sex was not considered in experimental design.
Field-collected samples	No field-collected samples were used in this study.
Ethics oversight	All the animal operations were following the laboratory animal guidelines and were approved by the Animal experimentatins Ethics Committee of Xinhua Hospital Affiliated to Shanghai Jiaotong University School of Medicine.

Note that full information on the approval of the study protocol must also be provided in the manuscript.

Flow Cytometry

Plots

Confirm that:

☒ The axis labels state the marker and fluorochrome used (e.g. CD4-FITC).

☒ The axis scales are clearly visible. Include numbers along axes only for bottom left plot of group (a 'group' is an analysis of identical markers).

☒ All plots are contour plots with outliers or pseudocolor plots.

☒ A numerical value for number of cells or percentage (with statistics) is provided.

Methodology

Sample preparation	SW480/ HIEC-6 cells were cultured in 6-well plates to 70–80% confluence. The cells were treated with DMSO or MAIT-516 for 48 h. Then, the cells were collected by trypsinization and washed with ice cold phosphate buffered saline (PBS). PI/ Annexin V-FITC assay was measured by PharmingenTM Annexin V Apoptosis Detection Kit (BD Biosciences) according to the manufacturer's instructions. Briefly, the harvested cells were incubated in 300 μL of 1× binding buffer containing 5 μL Annexin V and 5 μL PI for 30 min at room temperature in dark. Apoptosis of cells was measured and analyzed by flow cytometry.
Instrument	Beckman CytoFlex S
Software	Beckman CytExpert (version 1.2) and FlowJo (version 10)
Cell population abundance	After the cell population is divided, the software will provide the specific number of cells in each population
Gating strategy	The main cell population is selected according to FSC/SSC parameters, and then the positive dye cell population is selected according to the negative control which does not be stained with the dye.

☒ Tick this box to confirm that a figure exemplifying the gating strategy is provided in the Supplementary Information.
