# Supplementary material for: Rational design of a sensitivity-enhanced tracer for discovering efficient APC–Asef inhibitors
Source: Nat Commun. 2022 Aug 24;13:4961. doi: 10.1038/s41467-022-32612-6 (PMC9402538; doi:10.1038/s41467-022-32612-6)
Supplement: Supplementary file 4 — Source Data [file 41467_2022_32612_MOESM4_ESM.zip › Source Data/7F6M-validation_reports/D_1300022977_val-report-annotate_P1.pdf]

wwPDB X-ray Structure Validation Summary Report (i)

Jul 1, 2021 – 10:45 PM JST

PDB ID	:	7F6M
Title	:	Crystal structure of APC complexed with a peptide inhibitor MAI-516
Deposited on	:	2021-06-25
Resolution	:	2.40 Å(reported)

This is a wwPDB X-ray Structure Validation Summary Report.

This report is produced by the wwPDB biocuration pipeline after annotation of the structure.

We welcome your comments at *validation@mail.wwpdb.org*
A user guide is available at
https://www.wwpdb.org/validation/2017/XrayValidationReportHelp
with specific help available everywhere you see the (i) symbol.

The following versions of software and data (see references (i)) were used in the production of this report:

MolProbity	:	4.02b-467
Mogul	:	1.8.5 (274361), CSD as541be (2020)
Xtriage (Phenix)	:	1.13
EDS	:	2.22
Percentile statistics	:	20191225.v01 (using entries in the PDB archive December 25th 2019)
Refmac	:	5.8.0158
CCP4	:	7.0.044 (Gargrove)
Ideal geometry (proteins)	:	Engh & Huber (2001)
Ideal geometry (DNA, RNA)	:	Parkinson et al. (1996)
Validation Pipeline (wwPDB-VP)	:	2.22

1 Overall quality at a glance (i)

The following experimental techniques were used to determine the structure:
X-RAY DIFFRACTION

The reported resolution of this entry is 2.40 Å.

Percentile scores (ranging between 0-100) for global validation metrics of the entry are shown in the following graphic. The table shows the number of entries on which the scores are based.

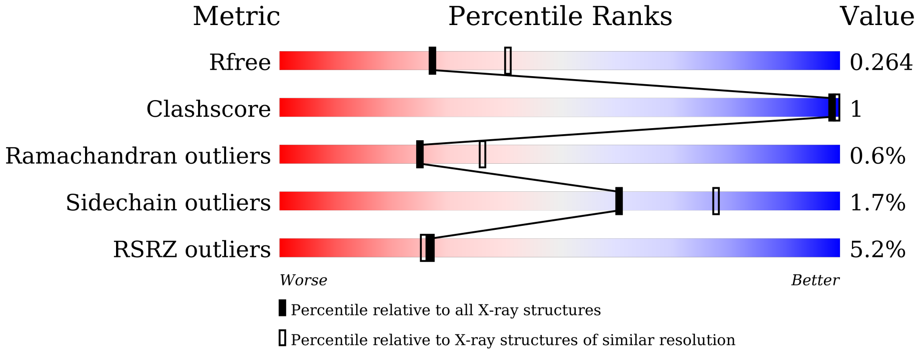

Metric	Whole archive (#Entries)	Similar resolution (#Entries, resolution range(Å))
R_{free}	130704	3907 (2.40-2.40)
Clashscore	141614	4398 (2.40-2.40)
Ramachandran outliers	138981	4318 (2.40-2.40)
Sidechain outliers	138945	4319 (2.40-2.40)
RSRZ outliers	127900	3811 (2.40-2.40)

The table below summarises the geometric issues observed across the polymeric chains and their fit to the electron density. The red, orange, yellow and green segments of the lower bar indicate the fraction of residues that contain outliers for >=3, 2, 1 and 0 types of geometric quality criteria respectively. A grey segment represents the fraction of residues that are not modelled. The numeric value for each fraction is indicated below the corresponding segment, with a dot representing fractions <=5% The upper red bar (where present) indicates the fraction of residues that have poor fit to the electron density. The numeric value is given above the bar.

Mol	Chain	Length	Quality of chain
1	A	353	5% 93% • •
2	B	9	100%

2 Entry composition (i)

There are 6 unique types of molecules in this entry. The entry contains 2861 atoms, of which 0 are hydrogens and 0 are deuteriums.

In the tables below, the ZeroOcc column contains the number of atoms modelled with zero occupancy, the AltConf column contains the number of residues with at least one atom in alternate conformation and the Trace column contains the number of residues modelled with at most 2 atoms.

- Molecule 1 is a protein called Adenomatous polyposis coli protein.

Mol	Chain	Residues	Atoms					ZeroOcc	AltConf	Trace
			Total	C	N	O	S			
1	A	341	2646	1644	481	494	27	0	0	0

There are 8 discrepancies between the modelled and reference sequences:

Chain	Residue	Modelled	Actual	Comment	Reference
A	399	GLY	-	expression tag	UNP P25054
A	400	HIS	-	expression tag	UNP P25054
A	401	HIS	-	expression tag	UNP P25054
A	402	HIS	-	expression tag	UNP P25054
A	403	HIS	-	expression tag	UNP P25054
A	404	HIS	-	expression tag	UNP P25054
A	405	HIS	-	expression tag	UNP P25054
A	406	MET	-	expression tag	UNP P25054

- Molecule 2 is a protein called MAI-516 inhibitor.

Mol	Chain	Residues	Atoms				ZeroOcc	AltConf	Trace
			Total	C	N	O			
2	B	9	64	41	8	15	0	0	1

- Molecule 3 is GLYCEROL (three-letter code: GOL) (formula: $C_3H_8O_3$).

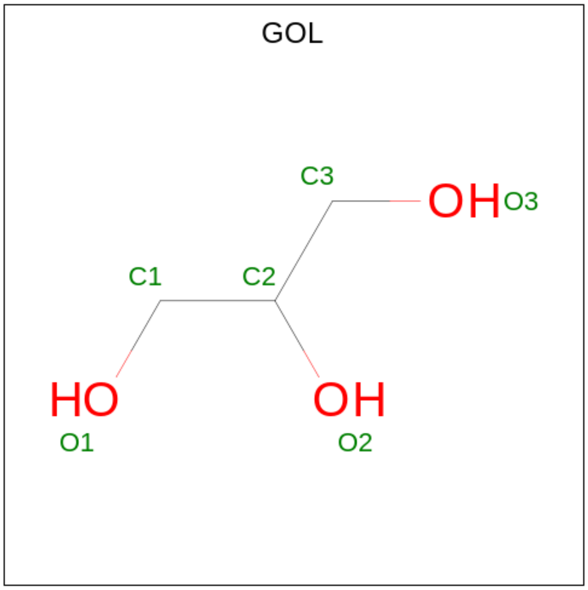

Mol	Chain	Residues	Atoms	ZeroOcc	AltConf
3	A	1	Total C O<br>6 3 3	0	0
3	A	1	Total C O<br>6 3 3	0	0
3	A	1	Total C O<br>6 3 3	0	0
3	B	1	Total C O<br>6 3 3	0	0
3	B	1	Total C O<br>6 3 3	0	0

- Molecule 4 is SULFATE ION (three-letter code: SO4) (formula: O_4S).

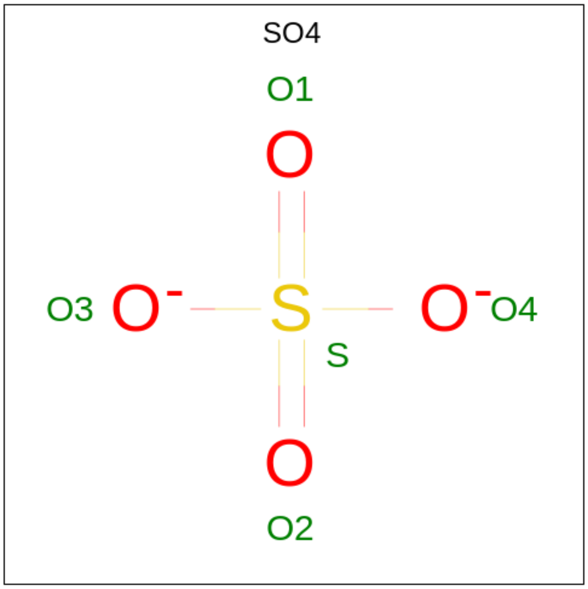

Mol	Chain	Residues	Atoms	ZeroOcc	AltConf
4	A	1	Total O S<br>5 4 1	0	0
4	A	1	Total O S<br>5 4 1	0	0

- Molecule 5 is DI(HYDROXYETHYL)ETHER (three-letter code: PEG) (formula: $C_4H_{10}O_3$).

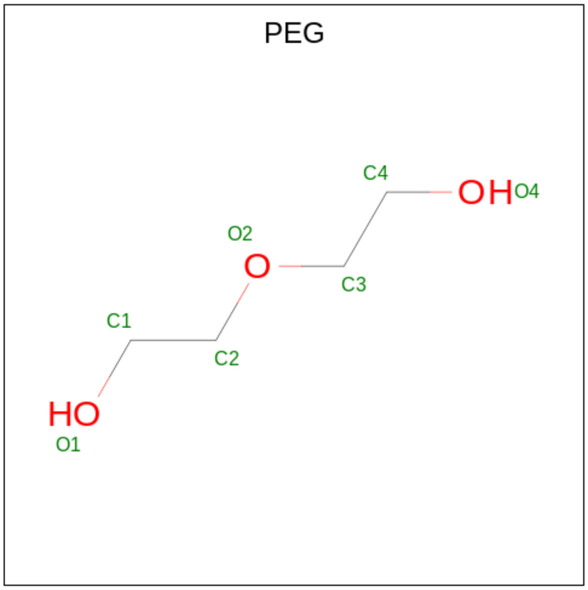

Mol	Chain	Residues	Atoms	ZeroOcc	AltConf
5	A	1	Total C O<br>7 4 3	0	0
5	B	1	Total C O<br>7 4 3	0	0

- Molecule 6 is water.

Mol	Chain	Residues	Atoms	ZeroOcc	AltConf
6	A	92	Total O<br>92 92	0	0
6	B	5	Total O<br>5 5	0	0

3 Residue-property plots (i)

These plots are drawn for all protein, RNA, DNA and oligosaccharide chains in the entry. The first graphic for a chain summarises the proportions of the various outlier classes displayed in the second graphic. The second graphic shows the sequence view annotated by issues in geometry and electron density. Residues are color-coded according to the number of geometric quality criteria for which they contain at least one outlier: green = 0, yellow = 1, orange = 2 and red = 3 or more. A red dot above a residue indicates a poor fit to the electron density (RSRZ > 2). Stretches of 2 or more consecutive residues without any outlier are shown as a green connector. Residues present in the sample, but not in the model, are shown in grey.

• Molecule 1: Adenomatous polyposis coli protein

Chain A:

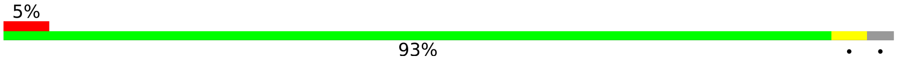

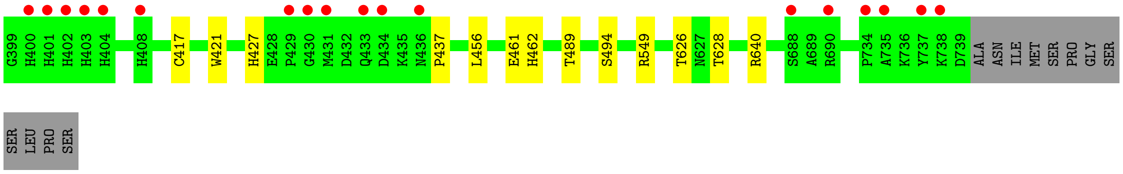

• Molecule 2: MAI-516 inhibitor

Chain B:

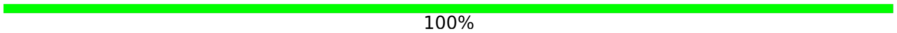

There are no outlier residues recorded for this chain.

4 Data and refinement statistics (i)

Property	Value	Source
Space group	C 1 2 1	Depositor
Cell constants<br>a, b, c, α, β, γ	87.75Å 82.34Å 49.74Å<br>90.00° 94.69° 90.00°	Depositor
Resolution (Å)	59.95 – 2.40<br>43.73 – 2.40	Depositor<br>EDS
% Data completeness<br>(in resolution range)	98.9 (59.95-2.40)<br>99.0 (43.73-2.40)	Depositor<br>EDS
R_{merge}	(Not available)	Depositor
R_{sym}	(Not available)	Depositor
$< I/\sigma(I) >$ [1]	3.44 (at 2.39Å)	Xtriage
Refinement program	REFMAC 5.8.0135	Depositor
R, R_{free}	0.208 , 0.256<br>0.217 , 0.264	Depositor<br>DCC
R_{free} test set	721 reflections (5.25%)	wwPDB-VP
Wilson B-factor (Å^2)	30.7	Xtriage
Anisotropy	0.125	Xtriage
Bulk solvent k_{sol}(e/Å^3), B_{sol}(Å^2)	0.38 , 40.7	EDS
L-test for twinning[2]	$< \|L\| > = 0.50$, $< L^2 > = 0.34$	Xtriage
Estimated twinning fraction	No twinning to report.	Xtriage
F_o,F_c correlation	0.92	EDS
Total number of atoms	2861	wwPDB-VP
Average B, all atoms (Å^2)	38.0	wwPDB-VP

Xtriage's analysis on translational NCS is as follows: *The largest off-origin peak in the Patterson function is 6.29% of the height of the origin peak. No significant pseudotranslation is detected.*

[1] Intensities estimated from amplitudes.

[2] Theoretical values of $< |L| >$, $< L^2 >$ for acentric reflections are 0.5, 0.333 respectively for untwinned datasets, and 0.375, 0.2 for perfectly twinned datasets.

5 Model quality (i)

5.1 Standard geometry (i)

Bond lengths and bond angles in the following residue types are not validated in this section: ANN, PEG, NH2, GOL, SO4

The Z score for a bond length (or angle) is the number of standard deviations the observed value is removed from the expected value. A bond length (or angle) with $|Z| > 5$ is considered an outlier worth inspection. RMSZ is the root-mean-square of all Z scores of the bond lengths (or angles).

Mol	Chain	Bond lengths		Bond angles	
		RMSZ	#$\|Z\|$ >5	RMSZ	#$\|Z\|$ >5
1	A	0.37	0/2691	0.56	0/3637
2	B	0.34	0/53	0.63	0/70
All	All	0.37	0/2744	0.56	0/3707

There are no bond length outliers.

There are no bond angle outliers.

There are no chirality outliers.

There are no planarity outliers.

5.2 Too-close contacts (i)

In the following table, the Non-H and H(model) columns list the number of non-hydrogen atoms and hydrogen atoms in the chain respectively. The H(added) column lists the number of hydrogen atoms added and optimized by MolProbity. The Clashes column lists the number of clashes within the asymmetric unit, whereas Symm-Clashes lists symmetry-related clashes.

Mol	Chain	Non-H	H(model)	H(added)	Clashes	Symm-Clashes
1	A	2646	0	2648	3	0
2	B	64	0	52	0	0
3	A	18	0	24	0	0
3	B	12	0	16	0	0
4	A	10	0	0	0	0
5	A	7	0	10	0	0
5	B	7	0	10	0	0
6	A	92	0	0	0	0
6	B	5	0	0	0	0
All	All	2861	0	2760	3	0

The all-atom clashscore is defined as the number of clashes found per 1000 atoms (including

hydrogen atoms). The all-atom clashscore for this structure is 1.

All (3) close contacts within the same asymmetric unit are listed below, sorted by their clash magnitude.

Atom-1	Atom-2	Interatomic distance (Å)	Clash overlap (Å)
1:A:626:THR:OG1	1:A:628:THR:HG22	2.20	0.41
1:A:417:CYS:HB3	1:A:421:TRP:CZ3	2.56	0.41
1:A:456:LEU:HD22	1:A:462:HIS:CD2	2.56	0.40

There are no symmetry-related clashes.

5.3 Torsion angles (i)

5.3.1 Protein backbone (i)

In the following table, the Percentiles column shows the percent Ramachandran outliers of the chain as a percentile score with respect to all X-ray entries followed by that with respect to entries of similar resolution.

The Analysed column shows the number of residues for which the backbone conformation was analysed, and the total number of residues.

Mol	Chain	Analysed	Favoured	Allowed	Outliers	Percentiles	
1	A	339/353 (96%)	330 (97%)	7 (2%)	2 (1%)	25	36
2	B	6/9 (67%)	5 (83%)	1 (17%)	0	100	100
All	All	345/362 (95%)	335 (97%)	8 (2%)	2 (1%)	25	36

All (2) Ramachandran outliers are listed below:

Mol	Chain	Res	Type
1	A	437	PRO
1	A	427	HIS

5.3.2 Protein sidechains (i)

In the following table, the Percentiles column shows the percent sidechain outliers of the chain as a percentile score with respect to all X-ray entries followed by that with respect to entries of similar resolution.

The Analysed column shows the number of residues for which the sidechain conformation was analysed, and the total number of residues.

Mol	Chain	Analysed	Rotameric	Outliers	Percentiles	
1	A	289/299 (97%)	284 (98%)	5 (2%)	60	78
2	B	5/5 (100%)	5 (100%)	0	100	100
All	All	294/304 (97%)	289 (98%)	5 (2%)	60	78

All (5) residues with a non-rotameric sidechain are listed below:

Mol	Chain	Res	Type
1	A	461	GLU
1	A	489	THR
1	A	494	SER
1	A	549	ARG
1	A	640	ARG

Sometimes sidechains can be flipped to improve hydrogen bonding and reduce clashes. All (1) such sidechains are listed below:

Mol	Chain	Res	Type
1	A	462	HIS

5.3.3 RNA (i)

There are no RNA molecules in this entry.

5.4 Non-standard residues in protein, DNA, RNA chains (i)

There are no non-standard protein/DNA/RNA residues in this entry.

5.5 Carbohydrates (i)

There are no monosaccharides in this entry.

5.6 Ligand geometry (i)

9 ligands are modelled in this entry.

In the following table, the Counts columns list the number of bonds (or angles) for which Mogul statistics could be retrieved, the number of bonds (or angles) that are observed in the model and the number of bonds (or angles) that are defined in the Chemical Component Dictionary. The Link column lists molecule types, if any, to which the group is linked. The Z score for a bond length (or angle) is the number of standard deviations the observed value is removed from the

expected value. A bond length (or angle) with $|Z| > 2$ is considered an outlier worth inspection. RMSZ is the root-mean-square of all Z scores of the bond lengths (or angles).

Mol	Type	Chain	Res	Link	Bond lengths			Bond angles		
					Counts	RMSZ	#|Z| > 2	Counts	RMSZ	#|Z| > 2
4	SO4	A	805	-	4,4,4	0.29	0	6,6,6	0.16	0
3	GOL	A	801	-	5,5,5	0.28	0	5,5,5	0.34	0
4	SO4	A	804	-	4,4,4	0.31	0	6,6,6	0.06	0
5	PEG	B	103	-	6,6,6	0.46	0	5,5,5	0.25	0
3	GOL	B	102	-	5,5,5	0.19	0	5,5,5	0.52	0
5	PEG	A	806	-	6,6,6	0.50	0	5,5,5	0.35	0
3	GOL	A	803	-	5,5,5	0.33	0	5,5,5	0.36	0
3	GOL	B	101	-	5,5,5	0.31	0	5,5,5	0.44	0
3	GOL	A	802	-	5,5,5	0.34	0	5,5,5	0.42	0

In the following table, the Chirals column lists the number of chiral outliers, the number of chiral centers analysed, the number of these observed in the model and the number defined in the Chemical Component Dictionary. Similar counts are reported in the Torsion and Rings columns. '-' means no outliers of that kind were identified.

Mol	Type	Chain	Res	Link	Chirals	Torsions	Rings
3	GOL	A	801	-	-	0/4/4/4	-
5	PEG	B	103	-	-	1/4/4/4	-
3	GOL	B	102	-	-	1/4/4/4	-
5	PEG	A	806	-	-	1/4/4/4	-
3	GOL	A	803	-	-	2/4/4/4	-
3	GOL	B	101	-	-	2/4/4/4	-
3	GOL	A	802	-	-	2/4/4/4	-

There are no bond length outliers.

There are no bond angle outliers.

There are no chirality outliers.

5 of 9 torsion outliers are listed below:

Mol	Chain	Res	Type	Atoms
3	A	802	GOL	O1-C1-C2-C3
3	A	803	GOL	C1-C2-C3-O3
3	B	101	GOL	O1-C1-C2-C3
3	A	802	GOL	O1-C1-C2-O2
5	A	806	PEG	O2-C3-C4-O4

There are no ring outliers.

No monomer is involved in short contacts.

5.7 Other polymers (i)

There are no such residues in this entry.

5.8 Polymer linkage issues (i)

There are no chain breaks in this entry.

6 Fit of model and data (i)

6.1 Protein, DNA and RNA chains (i)

In the following table, the column labelled '#RSRZ> 2' contains the number (and percentage) of RSRZ outliers, followed by percent RSRZ outliers for the chain as percentile scores relative to all X-ray entries and entries of similar resolution. The OWAB column contains the minimum, median, 95th percentile and maximum values of the occupancy-weighted average B-factor per residue. The column labelled 'Q< 0.9' lists the number of (and percentage) of residues with an average occupancy less than 0.9.

Mol	Chain	Analysed	<RSRZ>	#RSRZ>2			OWAB(Å^2)	Q<0.9
1	A	341/353 (96%)	0.14	18 (5%)	26	25	25, 35, 70, 107	4 (1%)
2	B	7/9 (77%)	-0.37	0	100	100	25, 26, 29, 30	0
All	All	348/362 (96%)	0.13	18 (5%)	27	26	25, 34, 70, 107	4 (1%)

The worst 5 of 18 RSRZ outliers are listed below:

Mol	Chain	Res	Type	RSRZ
1	A	434	ASP	7.5
1	A	433	GLN	4.7
1	A	400	HIS	4.4
1	A	738	LYS	4.3
1	A	735	ALA	3.9

6.2 Non-standard residues in protein, DNA, RNA chains (i)

There are no non-standard protein/DNA/RNA residues in this entry.

6.3 Carbohydrates (i)

There are no monosaccharides in this entry.

6.4 Ligands (i)

In the following table, the Atoms column lists the number of modelled atoms in the group and the number defined in the chemical component dictionary. The B-factors column lists the minimum, median, 95th percentile and maximum values of B factors of atoms in the group. The column labelled 'Q< 0.9' lists the number of atoms with occupancy less than 0.9.

Mol	Type	Chain	Res	Atoms	RSCC	RSR	B-factors(Å^2)	Q<0.9
5	PEG	B	103	7/7	0.65	0.37	63,64,64,65	0
3	GOL	A	801	6/6	0.66	0.26	46,47,48,50	0
3	GOL	A	802	6/6	0.77	0.33	58,59,60,61	0
5	PEG	A	806	7/7	0.81	0.18	50,51,52,52	0
3	GOL	B	102	6/6	0.82	0.29	52,52,52,53	0
3	GOL	A	803	6/6	0.84	0.24	48,49,50,50	0
4	SO4	A	804	5/5	0.93	0.13	67,69,71,71	0
3	GOL	B	101	6/6	0.96	0.19	36,36,36,36	0
4	SO4	A	805	5/5	0.99	0.10	30,30,31,31	5

6.5 Other polymers (i)

There are no such residues in this entry.
